# Warming up cool cooperators

**DOI:** 10.1038/s41562-023-01687-6

**Published:** 2023-09-14

**Authors:** Eamonn Ferguson, Claire Lawrence, Sarah Bowen, Carley N. Gemelli, Amy Rozsa, Konrad Niekrasz, Anne van Dongen, Lisa A. Williams, Amanda Thijsen, Nicola Guerin, Barbara Masser, Tanya E. Davison

**Affiliations:** 1 School of Psychology, University of Nottingham, Nottingham, UK; 2 National Institute for Health and Care Research Blood and Transplant Research Unit in Donor Health and Behaviour, Department of Public Health and Primary Care, University of Cambridge, Cambridge, UK; 3 Lawrence PsychAdvisory, Nottingham, UK; 4 School of Economics, University of Nottingham, Nottingham, UK; 5 Clinical Services and Research, Australian Red Cross Lifeblood, Melbourne, Victoria Australia; 6 Corporate Strategy and Transformation, Australian Red Cross Lifeblood, Melbourne, Victoria Australia; 7 Department of Psychology, Health, and Technology, University of Twente, Enschede, the Netherlands; 8 School of Psychology, University of New South Wales, Sydney, New South Wales Australia; 9 School of Psychology, The University of Queensland, Brisbane, Queensland Australia; 10 Monash Art, Design, and Architecture, Monash University, Melbourne, Victoria Australia

**Keywords:** Human behaviour, Economics, Human behaviour

## Abstract

Explaining why someone repeats high-cost cooperation towards non-reciprocating strangers is difficult. Warm glow offers an explanation. We argue that warm glow, as a mechanism to sustain long-term cooperation, cools off over time but can be warmed up with a simple intervention message. We tested our predictions in the context of repeat voluntary blood donation (high-cost helping of a non-reciprocating stranger) across 6 studies: a field-based experiment (*n* = 5,821) comparing warm-glow and impure-altruism messages; an implementation study comparing a 3-yr pre-implementation period among all first-time donors in Australia (*N* = 270,353) with a 2-yr post-implementation period (*N* = 170, 317); and 4 studies (*n* = 716, 1,124, 932, 1,592) exploring mechanisms. We show that there are relatively warm and cool cooperators, not cooling cooperators. Cooperation among cool cooperators is enhanced by a warm-glow-plus-identity message. Furthermore, the behavioural facilitation of future cooperation, by booking an appointment, is associated with being a warm cooperator. Societal implications are discussed.

## Abstract (web summary)

Ferguson et al. test the effectiveness of messages designed to increase rates of repeat blood donation and find that warm-glow feelings as a motivation for cooperation cool over time but can be reactivated.

## Main

Human cooperation presents the individual with the dilemma of deciding between what is good for them versus what is good for others^1^. This dilemma has been defined in the literature in a variety of ways ranging from paying a personal cost to benefit others^1^ to engaging in behaviours with mutual benefits^2–4^ that do not necessarily require coordinated action but may include reciprocal interaction^2,3,5^. Indeed, there is a clear clustering of cooperative behaviours, ranging from altruism, in terms of the simple division of resources without direct reciprocity (for example, dictator game), to more complex reciprocal interactions (for example, public good and trust games), that define the human cooperative phenotype^5^. Human society is characterized by a remarkably high degree of cooperation^1^ and for such high levels of cooperation to survive, definable benefits for the helper (and recipient) need to outweigh costs^1,4,6–8^. Indeed, several benefits (for example, reputation building) have been identified^1,7–21^. Here we examined one aspect of the cooperative phenotype—altruism—whereby the donor bears a cost to benefit a non-reciprocating stranger in the absence of any other mechanisms to support altruism (for example, costly punishment)^1,8,14,15,22^. Under such circumstances, we argue that warm glow sustains high-cost altruistic cooperation directly through reinforcement and indirectly by facilitating a behavioural commitment to cooperation. We tested these hypotheses using voluntary non-remunerated blood donation (VNRBD) as a real-world model of cooperation.

VNRBD follows the structure of the anonymous dictator and public goods games used to demonstrate warm glow in the lab^20,21,23–26^ and presents cooperation that incorporates both altruism^27–29^ and contributing to the public good^30,31^. Specifically, blood donors in a VNRBD system pay a cost to donate (for example, time, risk of fainting) to benefit a non-reciprocating stranger they will never meet (the recipient)^29^. Donation in a VNRBD system also contributes to the public good^30^, as donated blood is both non-excludable (freely available to anyone who needs it) and non-rivalry (there is sufficient blood, such that transfusing one person will not mean that another person cannot be transfused)^31^. This sufficiency of supply within a VNRBD system is maintained by transfusion services modelling and managing future supply and demand characteristics^32,33^.

Andreoni^11,20,21^ proposed that altruism can be motivated by one of the following: (1) warm glow, (2) pure altruism or (3) impure altruism. Warm glow refers to helping that is motivated solely by the positive feelings experienced as a result of helping and not by achieving a particular charitable outcome. In contrast, pure altruism refers to helping, not to feel good about helping, but solely to achieve a successful charitable outcome. Finally, impure altruism is a combination of warm glow and pure altruism: the giver experiences warm glow and wants to donate sufficient resources to ensure a successful outcome. As such, warm glow can be assessed either as experienced positive emotions arising from giving or behaviourally, in terms of individual contributions to a cause when these contributions result in no material benefit to that cause^23^ (Supplementary File 1 for details on assessing warm glow).

**Fig. 1:**
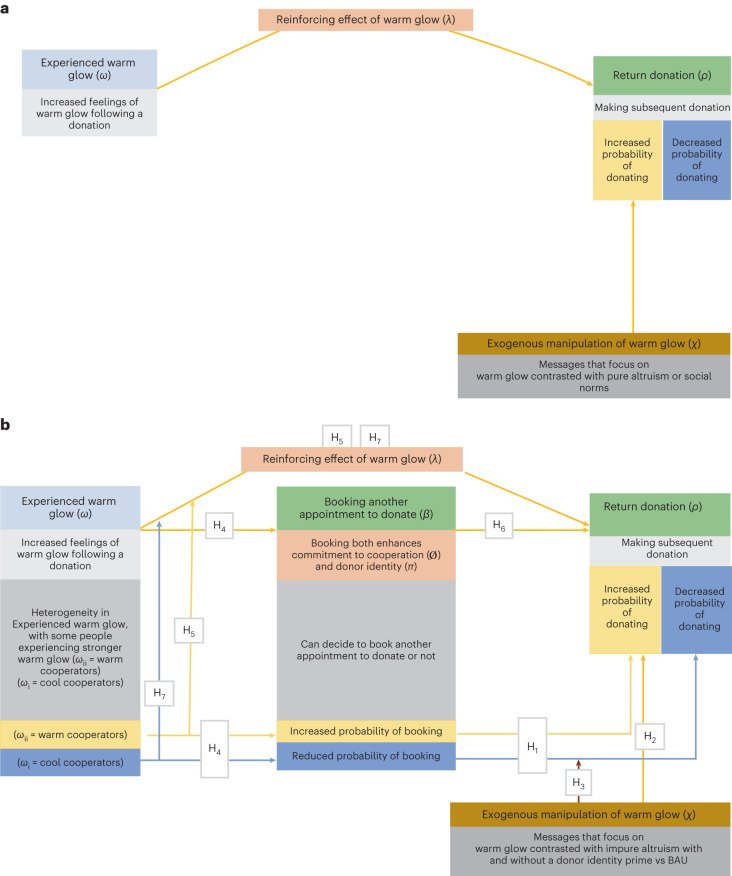
Theoretical models linking warm glow to cooperation. **a**, Current knowledge. Summary of the general findings supporting warm-glow theory. Specifically, people experience variation in warm glow from giving (*ω*) (see main text for factors that influence the extent of experienced warm glow) and this predicts future donations (*ρ*) based on the reinforcing effects of warm glow (*λ*) (see main text). Exogenous manipulations of warm glow (*χ*) predict repeat donations. **b**, Model to be tested. We extend **a** by suggesting an additional behavioural mechanism, booking another appointment to donate (*β*), which enhances commitment to cooperate (*Ø*) and identity as a donor (*π*). Overall increased feelings of experienced warm glow increase the probability of booking, with this being stronger for warm cooperators (those with higher levels of experienced warm glow) than cool cooperators (those with lower levels of experienced warm glow). Booking (*β*) predicts return donation (*ρ*), which is again stronger for warm cooperators. An exogenous manipulation of warm glow/impure altruism will increase the probability of making another donation if a donor identity prime is present. As cool cooperators are less likely to book and have their donor identity strengthened, the warm-glow/impure-altruism-plus-identity messages should be maximally effective for cool cooperators. Green, behavioural mechanism; peach, psychological mechanism; brown, exogenous manipulation; light blue, overall experienced warm-glow; yellow, effects linked to being a warm-cooperator; dark blue, effects linked to being a cool cooperator; greys, indications of associated behaviours or action.

The general hypotheses of warm-glow theory are supported by a large body of studies^23–26,34–48^ which is summarized in Fig. 1a. Specifically, evidence shows that people experience warm glow following helping^23,49–51^ (*ω*), with the degree of warm glow being proportional to the amount donated to charity^23,26^, with greater warm glow experienced when the donated resource was earned^45^. Furthermore, both the degree of experienced warm glow^37,49^ and exogenous manipulations of warm glow (*χ*)^52,53^ have been shown to predict future helping (*ρ*). A mechanism to account for why experienced warm glow predicts future helping is likely to be reinforcement arising from the expectation of experiencing warm glow again (*λ*)^23^. This reinforcing capacity of experienced warm glow is supported by evidence that warm glow is associated with: (1) activation of neural reward centres^36^, (2) increased effort to help charity^43^, (3) repeated acts of generosity^42^ and (4) the expectation of future reward^23^. Further, altruism is itself rewarding^51^. However, while exogenous manipulations of warm glow enhance cooperation, the mechanisms by which they achieve this are unknown^52,53^.

The current paper develops this understanding (Fig. 1b). We start from the premise that donors experience varying degrees of experienced warm glow (*ω*)^26–28,50^. Some experience stronger warm glow (*ω*_ii_: ‘warm cooperators’) than others (*ω*_i_: ‘cool cooperators’), specifically *ω*_ii_ > *ω*_i_. The function *ω*_ii_ > *ω*_i_ represents a relative relationship whereby cool cooperators still experience some warm glow but to a lesser extent than warm cooperators. Warm cooperators (*ω*_ii_) are predicted to have a greater probability of repeat cooperation via direct reinforcement effects of warm glow (*λ*). We also propose a second indirect behavioural mechanism, booking another appointment to donate during the donor’s current donation (*β*), through which stronger experienced warm glow increases the probability of repeat cooperation (*ρ*). Psychologically, booking will enhance a commitment to cooperate (*Ø*)^54^ and reinforce the donor’s self-identity as a donor (*π*)^54,55^, which in itself is a crucial predictor of prosociality^56,57^. A behavioural commitment to cooperate will also facilitate future cooperation through planning and scheduling (putting the date in the diary), which reduces the likelihood that a further donation will be derailed by competing commitments or forgetting^58^. Thus, booking an appointment will be a strong predictor of subsequent cooperation. Consistent with evidence that positive affect is associated with taking steps to facilitate future prosociality^59^, we predict that making a booking (commitment to cooperation) will be more likely for warm cooperators (*ω*_ii_) than for cool cooperators (*ω*_ii_).

For cool cooperators (*ω*_i_), the reinforcing effects of warm glow (*λ*) and the likelihood of booking another appointment (*β*) are both less likely. Therefore, we propose that an intervention based on warm glow (*χ*) will be more effective for these cool cooperators by eliciting feelings of warm glow. Further, as donor identity (*π*) strongly predicts repeat donation, a warm-glow message that also emphasizes donor identity will be more effective at encouraging cool cooperators to donate again. Therefore, a message that highlights both warm glow and donor identity should be especially effective for cool cooperators who can be identified behaviourally as those who do not book their next appointment to donate.

However, the reinforcing effect of warm glow (*λ*) may diminish over time, especially if the interval between cooperative acts is long, resulting in affective decay^60,61^ and unintentional free-riding^62,63^. Thus, we may have cooling cooperators. Research based on affective chronometry (the study of mood change over time) suggests that affect decays^60,61^. However, there is evidence that for positive affect augmenters, such as our warm cooperators, positive affect decays at a slower rate than for negative affect augmenters, such as cool cooperators^64^. Therefore, for cool cooperators (*ω*_i_), this decay may be more rapid than for warm cooperators (*ω*_ii_). Understanding the temporal dynamics of warm glow should help explain why warm-glow messages are more effective for cool cooperators, for example, if their warm glow decays more quickly^51^.

So far, two field-based experiments have exogenously manipulated warm-glow messages to test their impact on real-world cooperation^52,53^. Both experiments involved donating financial resources. The first study compared the effects of warm-glow messages (for example, “Please donate your refund – think of the good feeling in helping others.”) against norm-based messages (for example, “Many of our customers from this store regularly donate their refunds…”) displayed at supermarkets on the likelihood of donating bottle return refunds to charity^52^. Significantly higher donation rates were observed in supermarkets displaying warm-glow messages than supermarkets displaying norm-based messages^52^. However, as messages were randomly allocated by supermarket rather than by consumer, it cannot be ruled out that these results were due to differences in consumer populations between supermarkets^52^.

In the second field-based experiment, participants were randomly allocated to one of three message conditions: warm glow (‘… Warm your heart’), pure altruism (“… Make Alaska better for everyone”) and a no-treatment control (no message). The likelihood of participants donating to a chosen charity following a financial windfall and the amount donated were higher with exposure to the warm-glow than to the other messages^53^.

We further developed this work on the effectiveness of exogenous warm-glow messages. While previous research compared warm glow with pure altruism^53^, studies have consistently shown that blood donation is unlikely to be an example of pure altruism^27–29,50,65–67^. However, impure-altruism may motivate blood donors^27,65,66^. Therefore, we contrast warm-glow with impure-altruism messages; our warm-glow message focuses on the donor’s warm glow with no reference to benefits to recipients, whereas our impure-altruism message focuses both on the donor’s warm glow and benefits to recipients (Supplementary File 2 and Table 1). Thus, both warm-glow and impure-altruism messages feature warm glow as an active ingredient of the message. However, the impure-altruism message also includes pure altruism as an active ingredient (benefits to the recipient). Thus, we tested whether warm-glow or impure-altruism messages would enhance return donations more, especially for those who had not committed to a repeat donation (cool cooperators).

We further explored whether priming donor identity would enhance the effectiveness of warm-glow and impure-altruism messages. We predicted that (1) messages including a donor-identity prime would be more effective than those that did not prime identity and, (2) messages with a higher number of active elements would be more effective than those with fewer active elements. Therefore, we predicted that the rank order of message effectiveness would be: ‘impure-altruism-plus-identity’, ‘warm-glow-plus-identity’, ‘impure-altruism-only’ and finally ‘warm-glow-only’ (Supplementary File 2).

Overall, the aim of this paper is to extend the existing literature on warm glow and test key predictions outlined in Fig. 1b. Specifically: (1) the effectiveness of warm-glow /impure-altruism messages will be enhanced when presented in conjunction with a donor-identity prime, (2) warm-glow/impure-altruism messages with a donor-identity prime will be most effective for those who have not made a commitment to cooperation, (3) experienced warm glow will have a direct effect on future cooperation, (4) experienced warm glow will have an indirect effect on future cooperation through a commitment to cooperation and (5) experienced warm glow decays over time, with that decay being greater for cool vs warm cooperators. We addressed these aims by testing the hypotheses set out in Table 1 (hypotheses marked with a ~ were pre-registered) across six studies.

**Table 1:** Detailed hypotheses by study

Hypothesis	Study 1	Study 2	Study 3	Study 4	Study 5	Study 6
**Behavioural effects**						
H_1_: Commitment-to-cooperate effect: ‘Those who book another appointment will be more likely to return than those who do not book.’~	✓	✓		✓	✓	
H_2_: Effect of the warm-glow message: ‘Compared to a BAU control, the impure-altruism-plus-identity and warm-glow-plus-identity messages will have the strongest effects on donor return.’~	✓	✓				
H_3_: Message by donor commitment-to-cooperate effect: ‘A message that contains an identity component (warm-glow and/or impure-altruism) will have a significant effect on donor return only in those who did not book another appointment.’~	✓	✓				
**Behavioural mechanisms**						
H_4_: Experienced warm-glow commitment-to-cooperation effect: “Experienced warm glow will be higher in those who have booked another appointment compared to those who have not.”			✓	✓	✓	
H_5_: Direct effect of experienced warm glow: “Increased experienced warm glow predicts retuning to make a repeat donation.”				✓	✓	
H_6_: Indirect effect of experienced warm glow: “Experienced warm glow will be indirectly linked to return donor behaviour through making a commitment to cooperate (booking another appointment).”~				✓		
H_7_: Temporal dynamics of warm glow: “Warm glow will decay over time, especially for cool cooperators (those who have not booked another appointment).”					✓	
**Treatment messages: active ingredient**						
H_8_: Affordance of warm glow: “Both warm-glow and impure-altruism messages should afford more experienced warm glow, compared to BAU message.”						✓
H_9_: Identity enhances warm glow: “Experienced warm glow should be greater in the presence of an identity prime.”~						✓
H_10_: Warm-glow messages focus on the donor: “The warm-glow compared to the impure-altruism message should focus more on the donor and less on the recipient or both donor and recipient.”~						✓
H_11_: Impure-altruism messages focus on maintaining blood supply: “The impure-altruism compared to the warm-glow message should focus more on maintaining blood supply (the public good).”~						✓
H_12_: “Focus on the donor mediates the association between exposure to a warm-glow-plus-identity message and experienced warm glow.”~						✓

**Studies and design.** Study 1. Longitudinal field-based experiment: 5,821 first-time blood donors were randomly allocated to one of five warm-glow and impure-altruism conditions (with or without an identity prime) and a BAU control. Study 2. Pre-, post-implementation study comparing donor return rates in 270,353 first-time donors before the implementation of the warm-glow-plus-identity message with 170,317 donors post-implementation. Study 3. Survey of 716 first-time donors. Study 4. Survey of 1,124 donors, crossing donor status (first-time, novice) with donor type (plasma, blood). Study 5. Longitudinal study with 932 first-time donors by measuring warm glow at two timepoints (baseline). Study 6. Online between-subjects experiment: 1,592 members of the public were randomized to one of six conditions. ~Pre-registered prediction. All other behavioural effects and behavioural effects hypotheses were developed in the initial paper and tested formally in the subsequent revisions. Treatment-message hypotheses were developed in response to reviewers’ comments. Data from studies 3 to 5 were secondary data analyses to address components of the warm-glow model in the initial submission. Study 6 was designed to test hypotheses developed after the initial submission of the paper.

**Fig. 2:**
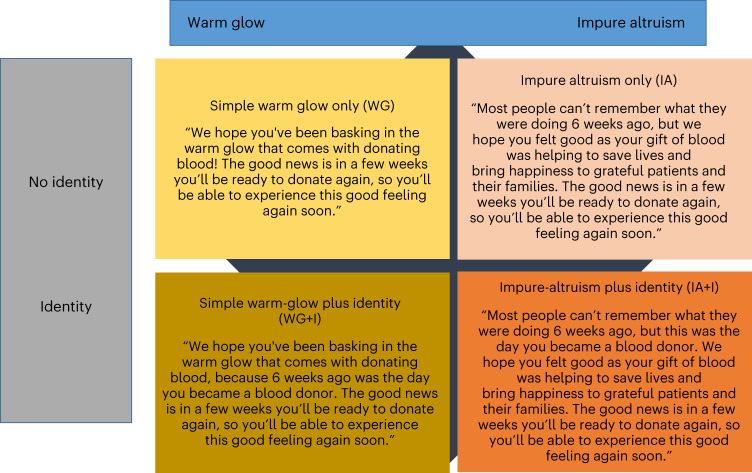
Treatment messages used in Studies 1 and 6. The messages formed when warm glow (warm glow, impure altruism) is crossed with identity (present, absent).

Studies 1 and 2 test behavioural hypotheses 1–3 and address the main question: “Compared to a business-as-usual (BAU) control, does a warm-glow/impure-altruism message plus an identity prime encourage repeat donation, specifically among those who have not booked an appointment for a future donation?” Study 1 is a field-based experiment that examined donor return behaviour as a function of booking status (booked, not booked) and four active message arms created by crossing message type (warm-glow, impure-altruism) with the donor identity prime (“… that’s when you became a blood donor …”: yes, no) (see Fig. 2 and Supplementary File 2 for details of message structure) compared to a BAU control. First-time blood donors (5,821) were randomly allocated to one of the five treatment conditions. Study 2 tested the most effective message from the field-based experiment (warm-glow-plus-identity) in an implementation trial. This trial compared return behaviour among all first-time Australian blood donors in three 1-yr time windows before the warm-glow-plus-identity message was implemented (total *n* = 270, 353: 54.6% had not booked) and two 1-yr time windows after implementation (total *n* = 170,317: 65.6% had not booked).

In studies 3 to 5, we explored the behavioural mechanisms that explain the effectiveness of the ‘warm-glow-plus-identity’ message (hypotheses 4–7). These address the main question “Does experienced warm glow predict future cooperation directly and indirectly via a commitment-to-cooperation?”. In Study 3 (survey of 716 first-time donors), we evaluated whether booking status (booked, not booked) was associated with warm glow or pure altruism. Study 4 (survey of 1,124 donors) compared four groups of donors by crossing donor status (first-time, novice) with donor type (plasma, whole blood) to explore the association between booking status and experienced warm glow and whether experienced warm glow and booking status predicted return behaviour. Study 5 investigated the temporal dynamics of experienced warm glow (does warm-glow decay and, if so, is the rate of decay higher among those who did not book another appointment) among *n* = 932 first-time donors by measuring experienced warm glow at two timepoints (baseline, 12-week follow-up). Study 5 also explored the association between experienced warm glow, booking status and return behaviour. Study 4 also explored the indirect effect of warm glow on return behaviour via a commitment to cooperate. Study 6, an online experiment (*n* = 1,592), explored the active ingredients of the ‘warm-glow-plus-identity’ message that make it the most effective (hypotheses 8–12).

## Results

We first present the results of tests of the behavioural effects (hypotheses 1–3; Studies 1–2), followed by tests of the behavioural mechanisms (hypotheses 4–7; Studies 3–5) and the active elements of the treatment messages (hypotheses 8–12; Study 6).

### Study 1 Field-based experiment

Table 2 shows the number of donors by treatment (warm-glow-only, warm-glow-plus-identity, impure-altruism-only, impure-altruism-plus-identity and no-message BAU control), their implied commitment to cooperate (booked or did not book a future appointment) and return rates. As expected, based on the randomization to treatments and the fact that donors were exposed to the treatment messages after their booking decisions, the likelihood of booking did not statistically significantly vary across treatments (*χ*^2^_(4)_ = 4.198, *P* = 0.380, *φ* = .027). Thus, any variability in return rates is due to the treatment messages and cannot be attributed to initial variation in booking status.

**Table 2:** Descriptive breakdown of the number (percentage) of first-time donors by treatment and whether they booked or did not book an appointment to donate again and then attended to make a subsequent donation for Study 1

	All first-time donors	First-time donors who booked		First-time donors who did not book		
Treatment (message received)	Number of first-time donors assigned to each treatment	Number (%) of first-time donors	Number (%) who donated	Number (%) of first-time donors	Number (%) who donated	Comparison of return rate of those who booked vs those who did not^a^
BAU	1,161	416 (35.8)	277 (66.6)	745 (64.2)	278 (37.3)	*χ*^2^_(1)_ = 91.660, *P* < 0.001, *φ* = 0.281
WG	1,156	428 (37.0)	292 (68.2)	728 (63.0)	269 (37.0)	*χ*^2^_(1)_ = 105.539, *P* < 0.001, *φ* = 0.302
WG + I	1,158	439 (37.9)	266 (60.6)	719 (60.1)	307 (42.7)	*χ*^2^_(1)_ = 34.915, *P* < 0.001, *φ* = 0.174
IA	1,166	464 (39.8)	286 (61.6)	702 (60.2)	279 (39.7)	*χ*^2^_(1)_ = 53.616, p *P* < 0.001, *φ* = 0.214
IA + I	1,160	441 (38.0)	269 (61.0)	719 (62.0)	285 (39.6)	*χ*^2^_(1)_ = 49.983, p *P* < 0.001, *φ* = 0.208
Comparison BAU vs Combined treatments on return rate			*χ*^2^_(1)_ = 2.073 *P* = 0.150, *φ* = 0.031		*χ*^2^_(1)_ = 1.496 *P* = 0.226, *φ* = 0.020	

All tests are two-tailed and no adjustment for multiple comparisons was applied. WG, warm-glow-only; WG + I, warm-glow-plus-identity; IA, impure-altruism-only; IA + I, impure-altruism-plus-identity. An overall comparison of the BAU treatment to the combined message treatments shows that there was no overall effect of simply being exposed to a message (*χ*^2^_(1)_ = 0.211, *P* = 0.646, *φ* = 0.006); results in the last row also show no effect of simply being exposed to a message for those who booked and those who did not book. Results in the last column show that booking enhances return rates across all treatments. ^a^Exact *P* values for the comparison of return rates of those who booked vs those who did not for (1) BAU, *P* = 1.0277 × 10^−21^; (2) WG, *P* = 9.3047 × 10^−25^; (3) WG + I, *P* = 3.4445 × 10^−9;^ (4) IA, *P* = 2.4376 × 10^−113^; and (5) IA + I, *P* = 1.5511 × 10^−12^.

#### Effect of behavioural commitment (H_1_)

The final column of Table 2 shows that for each treatment group, making a booking resulted in higher return rates.

#### Behavioural effects of warm glow (H_2_ and H_3_)

First, we compared whether exposure to an active message, regardless of the message content (combined across the four treatments) influenced return rates. The results comparing return rates for the no-message BAU control to those who were exposed to an active message, regardless of content, are presented in Table 2 (last row). The results show that return rates were not significantly different following exposure to any active message, both for donors who had booked and those who had not. Thus, we found no evidence that simple exposure to any message, regardless of content, influenced return rates.

Next, we tested whether exposure to a specific treatment message (for example, warm-glow-plus-identity or impure-altruism-plus-identity) increased return rates more than exposure to the BAU control among those who booked or those who did not book. The ‘warm-glow-plus-identity’ message was the only significant predictor of return rates among those who had not booked another appointment, and there were no significant effects among those who had booked (Supplementary File 2 and Table 2).

To consolidate the above univariate analyses, we conducted an intention-to-treat analysis with treatment, booking status and the treatment by booking-status interaction predicting returning to donate. These analyses controlled for other salient predictors of return to donate (age, gender and blood type). A logistic regression model (Table 3) indicated that the likelihood of returning increased with age and was higher among O− blood donors than O+ donors. Donors exposed to the ‘warm-glow-plus-identity’ message were 1.279 times more likely to return than the BAU control. No other treatment messages impacted return significantly relative to the BAU control. A significant main effect of booking status showed that, overall, donors who booked another appointment were 3.242 times more likely to return than those who had not booked.

**Table 3:** Logistic regression predicting return behaviour at 3 months in first-time donors

				95% CI		
	*B* (s.e.)	*P*	OR	Lower	Upper	Cohen’s *d*
Gender	−0.03 (0.06)	0.598	0.971	0.896	1.084	−0.016
Age	0.023 (0.002)	<0.001	1.023	1.019	1.028	0.012
Blood group		0.022				
A−	0.098 (0.115)	0.394	1.103	0.880	1.382	0.054
A+	−0.009(0.060)	0.838	0.991	0.880	1.116	−0.005
O−	0.266 (0.093)	0.004	1.304	1.087	1.565	0.146
Message treatment		0.120				
WG + I	0.246 (0.108)	0.023	1.279	1.035	1.580	0.136
WG	−0.010 (0.109)	0.928	0.990	0.800	1.226	−0.005
IA + I	0.105 (0.109)	0.334	1.111	0.898	1.374	0.058
IA	0.111 (0.109)	0.308	1.118	0.903	1.384	0.061
Booking status	1.176 (0.130)	<0.001	3.242	2.513	4.182	0.648
Interactions		0.004				
Booking status × WG + I	−0.506 (0.180)	0.005	0.603	0.424	0.859	−0.279
Booking status × WG	0.097 (0.184)	0.596	1.102	0.768	1.581	0.053
Booking status × IA + I	−0.335 (0.180)	0.064	0.715	0.502	1.019	−0.185
Booking status × IA	−0.297 (0.180)	0.098	0.743	0.523	1.057	−0.164
Intercept	−1.263 (0.119)	<0.001	0.283			
*R*^2^	0.10					

Gender (0 = men; 1 = women); blood group O+, reference category; booking status (0 = did not book another appointment to donate; 1 = did book another appointment to donate). The reference group for the message treatments is the BAU no message control. All tests are two-tailed and no adjustment for multiple comparisons was applied. *B* coefficients are unstandardized coefficients. Exact *P* values for (1) age, *P* = 9.5071 × 10^−23^; (2) booking status, *P* = 1.4149 × 10^−19^; and (3) constant, *P* = 9.6695 × 10^−27^.

The effect of booking status interacted significantly with the ‘warm-glow-plus-identity’ message (*B* = −0.506, s.e. = 0.180; *P* = 0.005; odds ratio (OR) = 0.603; 95% confidence interval (CI) = 0.424, 0859). Margins analysis was used to explore this interaction. Compared with the BAU control, those in the ‘warm-glow-plus-identity’ treatment who had not booked were significantly more likely to return (d*x*/d*y* = 0.058, s.e. = 0.025; *P* = 0.023; 95% CI = 0.008, 0.108; Cohen’s *d* = 0.133), but for those who had booked, there was no statistically significant variation (d*x*/d*y* = −0.060, s.e. = 0.033; *P* = 0.070; 95% CI = −0.124, 0.005; Cohen’s *d* = −0.143) (Supplementary File 2, and Tables 3 and 4).

### Study 2 Implementation analysis

The ‘warm-glow-plus-identity’ message was implemented and sent to all first-time donors in Australia, 6 weeks after their first donation (Supplementary File 3). We compared return rates among those who had booked another appointment with those who had not booked, across three time windows preceding the implementation and two time windows following the implementation. The number of first-time donors in each time window, the number who booked or not and the number who returned to donate can be found in Supplementary File 3, Fig. 1 and Table 5.

**Fig. 3:**
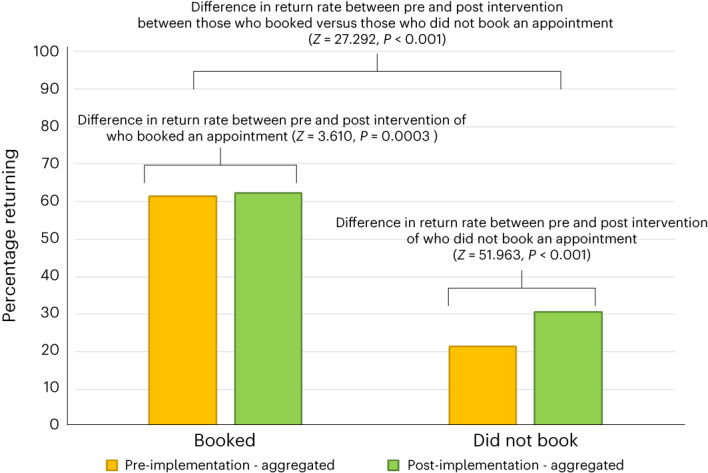
Aggregate return behaviour pre- and post-implementation of the ‘warm-glow-plus-identity’ message in new donors as a function of booking status (Study 2). For the pre-implementation, the aggregated point estimate for the percentage return rate for those who booked was 61.47 (95% CI = 61.20, 61.74; *n* = 75,414/122,681) and for those who did not book, 21.49 (95% CI = 21.28, 21.70; *n* = 31,738/147,672). For the post-implementation, the aggregated point estimate for the percentage return rate for those who booked was 62.35 (95% CI = 61.96, 62.74; *n* = 36,472/58,500) and for those who did not book, 30.55 (95% CI = 30.28, 30.82; *n* = 34,156/111,817). For those who had booked, there was a 0.88% (95% CI 0.402, 1.358) significant (*Z* = 3.610, *P* = 0.0003) increase in returning donors. For those who had not booked there was a 9.06% (95% CI = 8.718, 9.402) significant (*Z* = 51.963, *P* < 0.001) increase in returning donors. There was a significant interaction with the percentage increase in return rates for those who had not booked, significantly greater than those who had booked (*Z* = 27.292, *P* < 0.001), with a percentage difference of 8.18% (95% CI = 7.593, 8.767). Analyses were conducted using procedures for *Z* tests for proportions detailed in refs. ^98,99^ and implemented in ZumaStat 4.0. All analyses were two-tailed and no adjustments were made for multiple comparisons. Error bars are 95% CIs.

We aggregated these data over the three pre-implementation time windows (*n* = 270,353, of which 122,681 booked and 147,672 did not book) and two slightly overlapping post-implementation windows (*n* = 170,317, of which 58,500 booked and 111,817 did not book). Figure 3 plots the aggregate return rates pre- and post-implementation for those who booked and those who did not. For those who had booked, there was a significant increase (0.88%; 95% CI = 0.402, 1.358; *Z* = 3.610; *P* < 0.001) in returning donors, whereas, for those who had not booked, the percentage increase was 9.06% (95% CI = 8.718, 9.402), which was significant (*Z* = 51.963, *P* < 0.001). There was a significant interaction with the percentage increase in return rates for those who had not booked, significantly greater than those who had booked (*Z* = 27.292; *P* < 0.001; % increase = 8.18%; 95% CI = 7.593, 8.768). Thus, the ‘warm-glow-plus-identity’ message positively affected return behaviour for both those who had booked and those who had not, but the effect was significantly larger for those who had not booked. Due to the slight overlap in the two post-implementation time windows, we also compared the two post-implementation windows separately with the pre-implementation window immediately before the post-implementation period. The pattern of results is identical to the aggregated analyses (see Supplementary File 3 and Fig. 2). We also ran a sensitivity analysis to adjust these aggregate return rates for aggregate age and percentage of women (see Supplementary File 3). The hypothesized positive effect of the warm-glow-plus-identity message for those who had not booked remained.

### Studies 3 to 5 Behavioural mechanisms

#### Warm glow higher in those who book an appointment (H_4_)

Secondary analysis of data from Studies 3 to 5 allowed us to test the hypothesis that those who book will report higher experienced warm glow (Table 4, panels A–C, respectively).

**Table 4:** Regression models from Studies 3 to 5

	Panel A					
	*B* (s.e.)	*P*	OR	95% CI for OR		Cohen’s *d*
				Lower	Upper	
Age	0.024 (0.005)	<0.001	1.024	1.014	1.035	0.013
Gender	0.229 (0.169)	0.171	1.257	0.906	1.743	0.126
Anticipated negative affect	0.066 (0.072)	0.356	1.068	0.928	1.230	0.036
Anticipated calmness	0.014 (0.067)	0.839	1.014	0.889	1.155	0.077
Anticipated warm glow	0.079 (0.048)	0.097	1.082	0.986	1.188	0.044
Pure altruism	−0.091 (0.077)	0.236	0.913	0.785	1.061	−0.050
Constant	−2.590 (1.127)	0.022	0.075	0.008	0.685	−1.428
*R*^2^ *(n)*	0.049 (716)					
	**Panel B**					
Warm glow	0.066 (0.015)	<0.001	1.068	1.037	1.100	0.036
Gender	0.108 (0.134)	0.420	1.114	0.857	1.449	−0.060
Age	0.001(0.006)	0.809	1.001	0.990	1.013	0.001
Donor status	0.087 (0.245)	0.723	1.090	0.675	1.761	0.048
Donation type	−0.216 (0.186)	0.245	0.806	0.560	1.160	−0.112
Donor status × Donor type	0.273 (0.291)	0.349	1.314	0.742	2.325	0.151
Constant	−0.206 (0.309)	0.505	0.813	0.443	1.491	−0.114
*R*^2^ (*n*)	0.020 (1124)					
	**Panel C**					
Gender	0.679 (0.208)	0.001		0.270	1.088	0.375
Age	−0.005 (0.007)	0.435		−0.019	0.008	−0.003
Booking status	0.597 (0.284)	0.036		0.040	1.152	0.329
Wave	0.078 (0.332)	0.809		−0.554	0.710	0.043
Booking status × Wave	0. 216 (0.398)	0.587		−0.564	0.996	0.119
Constant	14.316 (0.365)	<0.001		13.596	15.035	0.375
*n* (observations)	932 (1,864)					

Panel A (Study 3): association between ‘anticipated warm glow’ and pure altruism with booking status (0 = did not book another appointment to donate; 1 = did book another appointment to donate) and gender (0 = men; 1 = women). Panel B (Study 4): donor status, donor type and warm glow to predict booking status. Gender (0 = men; 1 = women); donor status (0 = first time; 1 = novice); donor type (0 = plasma; 1 = whole blood); booking status (0 = did not book another appointment to donate; 1 = did book another appointment to donate). Panel C (Study 5): predicting the stability of warm glow over 12 weeks. Booking status (0 or 1), wave (0 = wave 1; 1 = wave 4), gender (0 = men; 1 = women). All tests are two-tailed and no adjustment for multiple comparisons was applied. *B* coefficients are unstandardized coefficients.

As Andreoni’s model^11,20,21^ specifically contrasts warm glow with pure altruism, Study 3 was used to explore the relative effects of warm glow and pure altruism with respect to booking status (see Supplementary File 4 and Table 6 for the derivation of the measure of ‘anticipated warm glow’). We regressed (logistic model) booking status (0, did not book, 1, booked) on ‘anticipated warm glow’ and ‘pure altruism. ‘Anticipated warm glow’ was significantly associated with booking status (*B* = 0.0847, s.e. = 0.0423; *P* = 0.045; 95% CI = 0.0018, 0.1677), such that higher levels of ‘anticipated warm glow’ were associated with booking another appointment, whereas for pure altruism there was no significant variation (*B* = −0.0648, s.e. = 0.07503; *P* = 0.338; 95% CI = −0.2118, 0.0822). We explored whether this association remains when other affective states associated with blood donation—‘anticipated feelings of calmness’ and ‘anticipated negative emotions’ (see Supplementary File 4 and Table 6 for the derivation of these measures)—are included in the model, along with controlling for donors’ age and gender. The resulting logistic regression is reported in Table 4a. The positive effect of anticipated warm glow on booking status remained but was not significant (*P* = 0.097). Anticipated negative emotions were not significantly associated with booking, as was pure altruism or anticipated calmness (a Heckman selection model (Supplementary File 4 and Table 7) showed no evidence of selection bias).

Study 4 provided a replication of Study 3 with a different measure of warm glow while controlling for donor status (first-time or novice) and donor type (whole blood or plasma). A logistic regression model was specified to test the effects of reported warm glow on booking another appointment, controlling for age, gender, donor status (first-time donors or novice donors) and donation type (whole blood or plasma). Warm glow was significantly and positively associated with booking status, but neither donor status nor donation type was significantly associated with booking (Table 4, panel B; for additional analyses predicting warm glow and robustness checks for sample clustering, see Supplementary File 4 and Tables 8–10).

Study 5 also shows that booking status predicts reported warm glow, with those who book reporting higher warm glow.

Across these analyses, there is clear evidence that making a commitment to cooperate (booking another appointment to donate) is associated with higher levels of warm glow. However, the effect size varies. Thus, using the mean scores for warm glow for those who had and had not booked across studies 3, 4 and 5, we report a simple random effects meta-analysis using Comprehensive Meta-Analysis v.2. The results show that there was an overall significant positive effect across the three studies (standard difference in means = 0.209, s.e. = 0.052; 95%CI = 0.106, 0.312; *P* < 0.001; Cohen’s *d* = 0.201; *r* = 0.100). Thus, there is a consistent positive association between reported warm glow and booking status (see Supplementary File 4 for analysis details and Supplementary Fig. 3 for Forest plot).

#### Warm glow (H_5_) and booking (H_1_) predict future donation

Results from Studies 4 and 5 (Supplementary File 5, and Tables 11 and 12) showed that both experienced warm glow (Study 4: *B* (s.e.) = 0.041 (0.016); *P* = 0.008; Cohen’s *d* = 0.024; OR = 1.042; 95% CI = 1.011, 1.074; Study 5: *B* (s.e.) = 0.048 (0.013); *P* < 0.001; Cohen’s *d* = 0.026; OR = 1.049; 95% CI = 1.022, 1.076) and booking status (Study 4: *B* (s.e.) = 1.408 (0.147); *P* < 0.001; Cohen’s *d* = 0.776; OR = 4.088; 95% CI = 3.068, 5.448; Study 5: *B* (s.e.) = 0.803 (0.111); *P* < 0.001; Cohen’s *d* = 0.443; OR = 2.232; 95% CI = 1.795, 2.775) positively predicted return behaviour.

#### Experienced warm glow predicts donations via booking (H_6_)

**Fig. 4:**
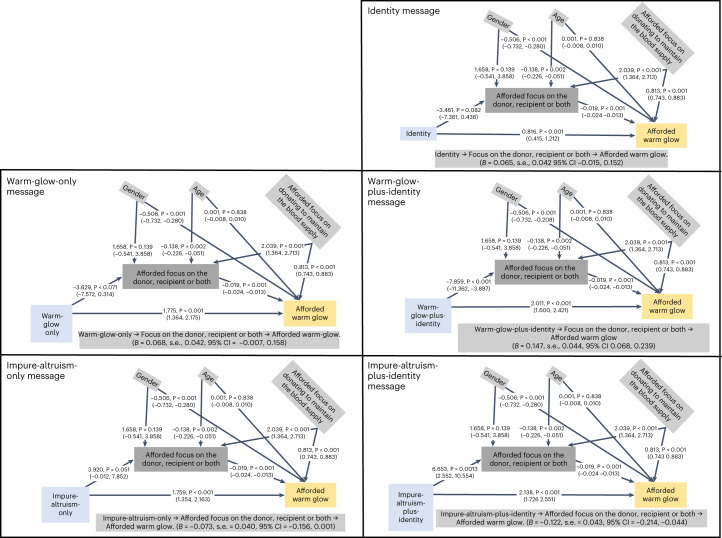
Mediation models specifying the effects of messages to predict ‘afforded warm glow’ directly and indirectly via a focus on the donor, recipient or both (Study 6). These models specify the effects of messages (*X*) to predict the outcome (*Y*) ‘afforded warm glow’ directly and indirectly via focus (‘afforded focus on the donor, recipient or both’ (the mediator, *M*)). These models were specified in PROCESS 4.0, with estimates based on 5,000 bootstraps with a multinominal *X* (messages) and BAU as the reference (*n* = 1,521). Age, gender and supply (‘afforded focus on donating to maintain blood supply’) were specified as confounders of *M* and *Y*. Predicting *M* from *X* has an *R*^2^ of 0.086, *P* < 0.001. Predicting *Y* from both *X* and *M* has an *R*^2^ of 0.333, *P* < 0.001. All analyses were two-tailed and no adjustments were made for multiple comparisons.

We proposed a path model (detailed in Supplementary File 5 and Fig. 4) that specified that experienced warm glow positively affected return behaviour via booking. The indirect effect of experienced warm glow on return via booking another appointment was significant (*B* = 0.022, s.e. = 0.006; *P* < 0.001; 95% CI = 0.013, 0.031).

#### Warm glow decays quicker in those not booking (H_7_)

We hypothesized that experienced warm glow cools over time and that this cooling would be quicker for those who did not book. We test this prediction using longitudinal data from Study 5, in which experienced warm glow was assessed 1 day (wave 1) and 12 weeks (wave 4) post-donation in those who had booked and those who had not. Table 4c shows the results of a generalized estimating equations model evaluating whether booking status predicted experienced warm glow across time. Results showed that those who booked reported higher average levels of experienced warm glow overall (*M* = 15.32, s.e. = 0.155) than those who did not (*M* = 14.68, s.e. = 0.215). There was no significant effect of time on experienced warm-glow levels or significant moderation effect of time by booking status. This indicates that the association of booking status on experienced warm glow was stable over time. Thus, rather than having cooling cooperators, we have cool cooperators (who do not book another appointment) and warm cooperators (who booked another appointment).

### Study 6 The active ingredients of warm-glow-plus-identity

It is important to identify the active ingredients of the ‘warm-glow-plus-identity’ message that prompts its effectiveness. To reflect theory, the warm-glow messages used across the studies focused solely on the donor, with no reference to recipient benefits, while the impure-altruism messages focused on both the donor and the recipient^11^. Thus, both warm-glow and impure-altruism messages should afford experienced warm glow more than a BAU control, as both contain active warm-glow ingredients (H_8_), with afforded warm glow greater when a person’s identity as a donor is primed (H_9_). Furthermore, warm-glow messages, compared with impure-altruism messages, should focus more on the donor than the recipient (H_10_) and less on donating to maintain the public good (blood supply) (H_11_). We predict that the warm-glow-plus-identity message is effective because the feelings of warm glow it generates are about the donor. Thus, exposure to a ‘warm-glow-plus-identity message’ should be indirectly linked to afforded warm glow through an enhanced focus on the donor relative to the recipient or both the donor and the recipient (H_12_).

We tested these predictions using data from Study 6 in which participants were exposed to one of the four messages used in the field-based experiment. We assessed each message’s ability to elicit: (1) ‘afforded experienced warm glow’ (possible range 2 to 14, where 14 is high warm glow), (2) a focus on the ‘donor, the recipient or both’ (−50 is focus on the blood donor, 0 is focus equally on both the donor and the recipient, and +50 is focus on the recipient) and (3) perceptions about donating to maintain blood supply (1, not at all; to 7, completely). We included two control conditions: a simple reminder BAU control condition and an identity-prime-only condition.

Supporting H_8_, the warm-glow and impure-altruism messages were judged to afford more warm glow than BAU (Supplementary File 6 and Fig. 5, and the planned contrasts in File 6). Warm-glow messages were judged to afford slightly lower warm glow than the impure-altruism messages (Supplementary Table 13). Supporting H_9_, exposure to an identity prime resulted in greater afforded warm glow (*F*_(1, 1,047)_ = 24.126; *P* < 0.001; *η*_*p*_^*2*^ = 0.023; *Mean*_identity_ = 11.714; 95% CI = 11.510, 11.917 vs *Mean*_no-identity_ = 11.418; 95% CI = 11.220, 11.615). Supporting H_10_, impure-altruism messages compared with warm-glow messages afforded higher ratings for donating to maintain blood supply (*F*_(1, 1,048)_ = 106.842; *P* < 0.001; *η*_*p*_^*2*^ = 0.093; *Mean*_impure-altruism_ = 4.988; 95% CI = 4.846, 5.131 vs *Mean*_warm glow_ = 3.917; 95% CI = 3.772, 4.062) (Supplementary Table 14). Supporting H_11_, warm-glow messages afforded greater relative donor focus than the impure-altruism messages (*F*_(1, 1,008)_ = 92.500; *P* < 0.001; *η*_*p*_^*2*^ = 0.084; *Mean*_warm glow_ = −29.299; 95% CI = −31.277, −27. 372 vs *Mean*_impure-altruism_ = −15.959; 95% CI = −17.881, −14.038) (Supplementary Table 15). Thus, warm-glow messages are more donor-focused and impure-altruism messages focus on both the donor and the recipient as well as on maintaining blood supply.

A series of mediation models with treatment message (*X*) predicting afforded warm glow (*Y*) directly and via an afforded focus on the donor, recipient or both (*M*) (Fig. 4) explored the hypothesis that the warm-glow-plus-identity message affords feelings of warm glow through a focus on the donor more than the recipient. All the messages, compared with the BAU control, predicted increased warm glow, as did a relative focus on the donor. There was a significant positive indirect effect of exposure to the ‘warm-glow-plus-identity’ message on levels of afforded warm glow via an afforded relative focus on the donor (*B* = 0.147, s.e. = 0.044; 95% CI = 0.068, 0.239). In contrast, the significant indirect effect of exposure to an ‘impure-altruism-plus-identity’ message was negative (*B* = −0.122, s.e. = 0.043; 95% CI = −0.214, −0.044). That is, exposure to an impure-altruism-plus-identity message is associated with an afforded greater recipient focus, linked to reduced afforded warm glow. There were no other significant indirect effects. Therefore, the effectiveness of the ‘warm-glow-plus-identity message’ is attributable to experienced warm glow being associated with an increased relative focus on donor identity.

## Discussion

We showed that warm glow is an important mechanism for sustaining high-cost cooperation directed towards a non-reciprocating stranger. We predicted that warm glow would decay and that exogenous warm-glow messages would act to remotivate cooling cooperators, especially among those who had not behaviourally committed to future cooperation. However, we found evidence that warm glow is stable over time. Therefore, we propose a revised model, whereby there are warm cooperators and cool (rather than cooling) cooperators, with a behavioural commitment to cooperate (booking another appointment) being associated with a warm cooperator, resulting in an increased likelihood of future cooperation.

Our observation that warm glow is stable is consistent with recent reports that warm glow, following blood donation, remains stable over several days^42,68^. However, further studies are needed to measure warm glow over time, both in the lab and in the field, to assess patterns of stability and decay for different types of helping and what predicts any variation in these patterns. Indeed, levels of experienced warm glow are proportional to the cost of cooperation^23,26^ and blood donation is a costly act of cooperation that is therefore likely to generate high levels of warm glow^69^. This may account for why experienced warm glow remains stable, even for cool cooperators. Less costly acts of cooperation may generate less warm glow that may decay^63^. Thus, we identified stable warm and cool cooperators of a high-cost cooperative act and reported evidence that warm glow among warm cooperators facilitated a smooth transition to repeat cooperation^51,59,70^. We show that this facilitation happens through two complementary mechanisms. First, a reinforcing route (*λ*)^36,49,51^ that signals a future reward^23^. Second, warm cooperators take behavioural steps through commitment to cooperate that facilitates the likelihood of returning to donate again^27,57^. In the context of blood donation, this is exemplified by booking the next donation, which minimizes derailment of future cooperation^58^ and potentially reinforces the donor’s self-image as a committed donor^55^. Thus, in the absence of an exogenous intervention, warm cooperators are more likely to return and cool cooperators drop out.

For any public health intervention to be effective, they have to contain active ingredients^67^. We tested predictions for the active ingredient of ‘warm-glow-plus-identity’ messaging and showed that all messaging afforded warm glow, which was increased most when this messaging was donor-focused rather than focused on benefit to the recipient. Thus, encouraging other repeat acts of cooperation at a societal level (for example, vaccination, social distancing, mask-wearing)^71–78^ may be achieved most effectively via warm-glow messages that focus not only on the feeling of warm glow but also link the warm glow directly to the person’s identity as a cooperator. For example, the altruism vaccination hypothesis suggests that people are motivated to get vaccinated to protect others^71–74^ (but see ref. ^75^). Emphasizing warm glow linked to the person’s identity as a good citizen may enhance the effectiveness of such messages (for example, “Be the person who makes a positive contribution to helping our society, get a vaccination to avoid spreading the flu and feel the warm glow from helping.”). Volunteer blood donation is sustained by 3–4% of the eligible population donating blood at any one time^29,67^. Many other cooperative acts aimed to help the wider society (for example, helping in a natural disaster or war zone) also rely on a few volunteers. Thus, our findings suggest that warm glow may be critical to ‘sustaining’ high-cost humanitarian aid in a small group of volunteers.

Improving conversion rates from first to repeat donations is a crucial objective for transfusion services worldwide, as repeat donors lead to savings on recruitment costs and improved donor safety^79–82^. Warm-glow messages offer a simple, low-cost and effective means to help achieve this.

Furthermore, behavioural commitment to cooperation by booking another appointment to donate was a very powerful predictor of future prosocial behaviour, therefore making it easier for people to book another appointment will be beneficial. Taking this further, facilitating commitments to cooperate in other prosocial areas (for example, signing up for a flu vaccination appointment, making a diary commitment for volunteering) would also be likely beneficial.

Although the effect sizes reported in this paper are small for experienced warm glow and exogeneous warm glow, a small effect size should be interpreted as important when the behaviour is hard to change (for example, making a repeated donation) but importantly, the intervention is simple and low cost, and the population effect is large^83^. Indeed, we showed that the ‘warm-glow-plus-identity’ message resulted in an extra 3,049 donations in the year following implementation (Supplementary File 3).

Our findings show that the effect of warm glow extends to cooperative behaviours beyond donating money to the donation of bodily tissue (blood). This is interesting as these acts differ in substantive ways. Many people donate money and this is often low cost in terms of time, effort and discomfort but can range from low to high cost in terms of finances^84–87^. However, few donate blood, which is generally high cost in terms of time, effort and potential discomfort^88^. Notably, those who donate only blood or only money differ on several demographic (blood donors are younger, more likely to be childless) and psychological characteristics (blood donors are less interested in politics and are more risk-taking)^87^. Furthermore, as levels of experienced warm glow are proportional to the cost of cooperation^23,26^, blood donation is likely to generate greater warm glow than less costly forms of cooperation^88^. It may be the case that a threshold exists for experienced warm glow, which once passed triggers sustained helping. Indeed, such a threshold model that becomes self-reinforcing has been suggested to sustain blood donor behaviour^67^.

Finally, we acknowledge a number of limitations in the reported studies. First, we used secondary data analyses in 3 studies (studies 3 to 5) to explore the association between experienced warm glow, booking status and behaviour. These studies were not designed to assess these associations specifically and, as such, replication of these findings are needed, especially using more widely ranging assessments of experienced warm glow and emotional reactions to blood donation^23^. Second, we assess the temporal dynamics of experienced warm glow at two timepoints, and it would be informative to assess warm glow across a greater number of timepoints post-donation and into subsequent donations to gain a more detailed understanding of the temporal dynamics of experienced warm glow as a function of booking status and time. Third, we did not assess exogenously manipulated warm glow and experienced warm glow together, hence we could not test directly whether the exogenous manipulation of warm glow enhances experienced warm glow in blood donors. However, the pattern of results across the studies indicates that the manipulation of warm glow, especially when donor identity is primed, does afford experienced warm glow and that this is associated with booking and return behaviour.

## Methods

### Ethics approvals and consent

Ethics approval for studies 1, 3, 4 and 5 was granted by the Australian Red Cross Lifeblood (formerly known as the Australian Red Cross Blood Service) Human Research Ethics Committee. Approvals were granted on 7 May 2018 for study 1 (Davison 04052018), 31 July 2020 for study 3 (Guerin 31072020), 18 September 2015 (2015#07) for study 4 and 15 November 2016 (2016#24) for study 5. Study 6 was approved by the University of Nottingham School of Psychology Ethics Committee (S1309). Informed consent was provided for studies 3 to 6. All donors attending to donate at the Australian Red Cross Lifeblood signed an individual general declaration consenting to assist blood donor research and as such, the Australian Red Cross Lifeblood Human Research Ethics Committee did not require additional consent for study 1 as the messages were sent out as part of the normal donor recruitment protocol and posed minimal risk. Study 2 drew on data aggregated at the population level, collected by the Australian Red Cross Lifeblood. As all donors signed a general declaration consenting to assist blood donor research when they attend to donate, no additional ethics approval was required for study 2.

### Study 1 Field-based experiment

#### Participant compensation

Participants did not receive compensation for this study.

#### Sampling and sample

A sample of 5,821 (Mean age = 31.94, s.d. = 11.96; % women = 60.7%) new whole-blood donors from across Australia with A−, A+, O− and O+ blood types (based on the Australian Red Cross Lifeblood donor segmentation policy) were recruited. However, A and O positive and negative groups are the most prevalent, accounting for 86% of Australian blood donors. Donors who had made their first whole-blood donation 6 weeks earlier and who had not donated previously in Australia were recruited. For each week of the field-based experiment, the entire eligible population was sampled, with all donors who met the eligibility criteria being selected and randomly allocated to conditions using simple randomization. This process continued until a priori target numbers per condition were achieved. Twenty donors were excluded: 12 due to email bounces and 8 due to being permanently deferred from donating blood after their initial donation, leaving a final sample of *n* = 5,801 (Mean age = 31.92, s.d. = 11.95; % women = 60.7%; see Supplementary File 7 for detail on sample bias and randomization checks, and Supplementary Fig. 9). This sample is generally representative when compared to the average age (32.5 yr) and percentage of women (56%) for all first-time whole-blood donors in the three pre-implementation windows in Study 2.

#### Procedure

New A−, A+, O− and O+ donors were recruited to the trial when they made their first donation between 16 April 2018 and 8 July 2018, with the first message issued on 22 May 2018. Donors were randomly allocated (using simple randomization) to one of four active arms and a BAU control arm. The control arm contained no message. All other communications from Lifeblood, including an SMS reminder that the donor could donate whole blood again sent 12 weeks post-donation, were identical across trial arms. Thus, the only difference between conditions was message type (warm-glow, impure-altruism) crossed with the donor identity prime (“… that’s when you became a blood donor …”: yes, no) (see Fig. 2 and Supplementary File 2 for details of message structure).

#### Measures

We measured donor characteristics including (1) age, (2) gender (0 = men; 1 = women), (3) blood group (ABO: A−, A+, O−, O+) and (4) whether they had booked to make their next donation immediately after their first donation (scored 1) or had not initially booked (scored 0).

#### Donor return behaviour

Donor return was measured as verified attendance at the donor centre to make a high-cost donation (whole blood or plasma) within 3 months after becoming eligible. These data were collected using eProgesa 5.03. The data analysts who extracted the attendance data were blind to experimental treatments.

#### Power analysis

Warm glow has a small effect on predicted blood donor attendance^53,55^. To achieve 80% power to detect a small effect with an alpha of 0.05 in a simple regression model with 8 predictors (for example, message type and identity prime conditions, age, gender, blood group, booking status and terms for the interaction of booking status with experimental conditions), 757 donors per arm are needed. Thus, we aimed for 1,000 new donors per arm to allow for ad hoc exploratory analyses.

#### Pre-registration

The trial was pre-registered on the Open Science Framework (OSF reference: https://osf.io/5m69k). Pre-registered predictions are detailed in Table 1.

### Study 2 Implementation study

#### Participant compensation

Participants did not receive compensation for this study.

#### Design

The most effective message of those tested in the field-based experiment was selected by Lifeblood to be rolled out nationally on 9 July 2018, with effectiveness measured by marketing click-through data (the highest proportion of click-to-open) from the original recruitment sample. Analysis of attendance data collected in the field-based experiment confirmed the click-rate message choice (see Supplementary File 8 and Fig. 10 for the timeline of the field-based experiment and implementation analysis). The implementation sample included all new donors across Australia who had donated whole blood, excluding donating for autologous and therapeutic reasons. We collated aggregate data on whether they had attended to make a second high-cost donation (whole blood or plasma) within 3 months and whether they had initially booked the next appointment in-centre. We compared the frequency of return in the 3 yr immediately before message roll-out with the frequency of return in two time windows after implementation. The 3-yr pre-implementation period covered three 12-month time windows: beginning on 16 April to 15 April for years (1) 2015–2016 (*n* = 90,317; Mean age = 32.0, s.d. = 13.6; % women = 55%, *n* = 49,778), (2) 2016–2017 (*n* = 93,430; Mean age = 32.5, s.d. = 13.4; % women = 56%, *n* = 52,539) and (3) 2017–2018 (*n* = 86,606; Mean age = 33.1, s.d. = 13.0; % women = 57%, *n* = 49,531). The post-implementation period covered two slightly overlapping time windows: (1) 9 July 2018 to 8 July 2019 (*n* = 81,766; Mean age = 34.9, s.d. = 13.5; % women = 57%, *n* = 46,689) and (2) 16 April 2019 to 15 April 2020 (*n* = 85,551; Mean age = 34.3, s.d. = 13.2; % women = 57%, *n* = 50,480). The first post-implementation window covers the 12 months from the moment the selected warm-glow message went live across Australia and the second window covers the April-to-April window consistent with the timing of the pre-implementation time windows.

#### Pre-registration

This study was not pre-registered. Rather, data were collated from the Australian Red Cross Lifeblood (via eProgesa 5.03) on a yearly aggregate level to test the prediction from the main finding of the field-based experiment that a warm-glow-plus-identity message would encourage more second-time donations in those who have not booked.

### Study 3 Booking, warm glow and pure altruism

#### Participant compensation

Five AU$100 gift vouchers were awarded after a random draw of participants.

#### Rationale

This study was designed primarily to examine blood donors’ concerns and attitudes towards blood donation during the coronavirus disease 2019 (COVID-19) pandemic. We used secondary data analysis to explore the association between anticipated warm glow (and other anticipated emotional states), pure altruism and booking status in first-time donors, as these were the focus of the field-based experiment.

#### Sample

Seven-hundred and sixteen first-time donors took part (Mean age = 41.20, s.d. = 14.85; % women = 65.5%, *n* = 469 (see Supplementary File 7 for sampling strategy)); the current sample was older and had a higher percentage of women.

#### Procedure

Donors were sent a de-identified online survey invitation between 12 August and 24 September 2020. A reminder was sent on 27 August 2020. Data were extracted on 24 September 2020. The survey was hosted on Qualtrics (https://www.qualtrics.com/). The survey was sent out after donors had made their donations and had the opportunity to book their next donation, but the time between booking and survey completion varied.

#### Measures

The following variables were used.

*‘*Anticipated warm glow’ was indexed using adjectives of the type that have been used to assess warm glow in previous research (for example, refs. ^24,44^). Specifically, donors were asked “What do you think donating blood in the future during the coronavirus pandemic would be like?”, to which they responded using 12 adjectives (Unrewarding, Pointless, Displeasing, Negative, Stressful, Unsatisfying, Rewarding, Worthwhile, Pleasing, Positive, Relaxing, Satisfying), each measured on a 4-point scale (1 = Not at all, 2 = Slightly, 3 = Moderately and 4 = Extremely). Of these adjectives, Rewarding, Worthwhile, Pleasing, Positive and Satisfying represent the type of adjective that have been used to assess warm glow in previous research (for example, ref. ^24^). A principle-axis-factor analysis with oblique rotation (Supplementary File 4 and Table 6) applied to these 12 adjectives indicated a 3-factor solution, with the ‘anticipated warm glow’ adjectives Rewarding, Worthwhile, Pleasing, Positive and Satisfying (*α* = 0.77) forming one factor^24^, the adjectives Relaxing and Stressful forming a factor whereby high scores equated to relaxing (termed ‘Anticipated calmness’; mean inter-item correlation = 0.34)^89^ and the adjectives Pointless, Negative, Unrewarding, Unsatisfying and Displeasing forming the final factor, which was termed ‘anticipated negative affect’ (*α* = 0.68)^89^.

‘Pure altruism’ was assessed with two items concerning the donor’s motivation for their last donation (“To help someone in need” and “It’s important for society”), each measured on a 5-point scale (1 = Not important at all, 2 = Slightly important, 3 = Moderately important, 4 = Very important, 5 = Extremely important).

#### Booking status

We extracted data about whether donors had booked another appointment after their last donation from participants’ linked but de-identified donor records (*n* = 294, 41.1% booked).

#### Additional selection model predictor

As the study took place during the COVID-19 pandemic, we included a question that assessed whether the donor had had a COVID-19 test (“Have you ever been tested for coronavirus?”: Yes, No).

#### Power analysis

There was no data on which to base a power calculation. However, if we assume a small effect size, then we would need 363 donors who booked and 363 who did not (*n* = 726) with a power of 0.80 and *α* = .05. In our sample, 294 booked and 422 did not. As such, the overall sample size was sufficient to detect a small effect.

#### Pre-registration

This study was not pre-registered.

### Study 4 Warm glow, booking status, donor status and type

#### Participant compensation

Participants did not receive compensation for this study.

#### Rationale

The study was designed primarily to examine how donors’ emotions change across a single donation. The study also included a measure of donors’ intrinsic motivation to donate blood^90^. Within the literature, warm glow has been conceptualized as intrinsic motivation^24,44,91–93^, and intrinsic regulation from self-determination theory has been linked to assessing warm glow^93^. Thus, warm glow was assessed using a subscale designed to measure intrinsic regulation for blood donation. In this study, we explored the association between booking and warm glow, controlling for donor status (first-time vs novice) and type (plasma vs whole blood).

#### Sample

In total, 1,124 donors (Mean age = 30.27, s.d. = 11.51; % women = 55.2%, *n* = 621 women) participated. Of these, 401 were first-time whole-blood donors (Mean age = 30.38, s.d. = 11.79; % women = 54.6%, *n* = 219), 208 were first-time plasma donors (Mean age = 31.07, s.d. = 11.50; % women = 51.0%, *n* = 106), 379 were novice whole-blood donors (Mean age = 29.61, s.d. = 11.49; % women = 60.4%, *n* = 229) and 136 were novice plasma donors (Mean age = 30.53, s.d. = 10.75; % women = 49.3%, *n* = 67) (see Supplementary File 7 for sampling strategy and details on sample bias). The sample was representative in terms of the percentage of women but slightly younger than the average age (32.5 yr) for first-time whole-blood donors in the three pre-implementation windows in Study 2.

#### Procedure

Participating donors were approached by a different researcher in each of three donation centres and prompted to complete questionnaires at several timepoints across their donation. The assessment of warm glow (that is, intrinsic regulation) was completed in the refreshment area after donation. Donors could book during the session. Data were collected between April 2016 and October 2017.

#### Measures

The following indices were assessed.

##### Warm glow (intrinsic motivation)

This was assessed using the intrinsic regulation subscale of the Blood Donor Identity Survey (BDIS)^90^, which comprises 3 items (“I enjoy donating blood”, “For me, being a blood donor means more than just donating blood” and “Blood donation is an important part of who I am”), with each item measured on a 7-point scale (1 = Not at all true, 7 = Very true). These items reflect the psychological concept of warm glow^24,44^ and were summed to yield a warm-glow score, with higher scores indicating higher subjective warm glow (Mean = 14.66, s.d. = 4.42, *α* = 0.80).

#### Booking status

We gained data about whether donors had booked another appointment during the donation session when they completed the survey from participants’ linked but de-identified donor records (781, 69.5% booked).

#### Donor return behaviour

Donor return was measured as verified attendance to donate within 6 months of the index donation, collected using eProgesa 5.03 (670 or 59.6% returned to donate).

#### Power analysis

There was no data on which to base a power calculation. However, if we assume a small effect size, then we would need 363 donors who booked and 363 who did not (*n* = 726) with a power of 0.80 and *α* = .05. In our sample, 781 booked and 343 did not. As such, the overall sample size was sufficient to detect a small effect.

#### Pre-registration

This study was not pre-registered.

### Study 5 Temporal stability of warm glow following booking

#### Participant compensation

Participants could opt-in to receive AU$5 compensation for every survey completed.

#### Rationale

The study was designed primarily to examine how donors’ emotions (reflective and anticipated) changed following their first donation. Warm glow was assessed at two of the four study timepoints: 1 day after donating blood (wave 1) and 12 weeks later (wave 4). We used these data for secondary data analysis to assess: (1) whether booking influenced warm glow, (2) the stability of warm glow over time and (3) whether the stability of warm glow was moderated by booking status.

#### Sample

Nine-hundred and thirty-two first-time donors who completed the survey provided warm-glow responses at waves 1 and 4 (Mean age = 36.36, s.d. = 14.02; % women = 70.3%, *n* = 655) (see Supplementary File 7 for sampling strategy and details on sample bias). Compared with the average age (32.5 yr) and percentage of women (56%) for first-time whole-blood donors in the three pre-implementation windows in Study 2, this sample was slightly older and had a higher percentage of women.

#### Procedure

In a longitudinal design, data were collected in four waves following donors’ first donation. The first (wave 1) assessments were completed approximately 1 day after the donor’s first donation. Participants who opted-in for follow-up were invited to complete assessments at three subsequent timepoints: 4 (wave 2), 8 (wave 3) and 12 (wave 4) weeks after their initial donation. Warm glow (intrinsic motivation) was measured in waves 1 and 4. All data were collected between January 2017 and September 2017.

#### Measures

The following measures were assessed.

##### Warm glow (intrinsic motivation)

This was assessed at waves 1 and 4 using the BDIS^88^ as in Study 4 (*Mean*_wave 1_ = 14.99, s.d._wave 1_ = 4.15, *α*_wave 1_ = 0.76; *Mean*_wave 4_ = 15.21, s.d._wave 4_ = 4.07, *α*_wave 4_ = 0.76).

#### Booking status

We gained data about whether donors had booked another appointment after their last donation from participants’ linked but de-identified donor records (*n* = 612, 65.7% booked).

#### Donor return behaviour

Return was measured as verified attendance to donate within 6 months of the index donation, collected using eProgesa 5.03 (*n* = 682, 73.2% returned to donate).

#### Power analysis

There was no data on which to base a power calculation. However, if we assume a small effect size, then we would need 363 donors who booked and 363 who did not (*n* = 726) with a power of 0.80 and *α* = .05. In our sample, 612 booked and 320 did not. As such, the overall sample size was sufficient to detect a small effect.

#### Pre-registration

This study was not pre-registered.

### Study 6 Validation of warm-glow and impure-altruism messages

#### Participant compensation

Based on Prolific’s rates, participants received £1.25 compensation for completing the study.

#### Experimental design

The main design was a 2 (warm glow: warm glow, impure altruism) by 2 (identity: present, absent) between-subjects design. This represents the four main messages used in the field-based experiment (Study 1) (warm-glow-only, warm-glow-plus-identity, impure-altruism-only and impure-altruism-plus-identity; Fig. 2). We added two additional conditions to compare warm-glow and impure-altruism messaging with (1) a no-message BAU control (“The good news is in a few weeks you’ll be ready to donate.”); and (2) an identity-prime-only condition (“Most people can’t remember what they were doing 6 weeks ago, but this was the day you became a blood donor. The good news is in a few weeks you’ll be ready to donate again.”). This was an online experiment hosted on Qualtrics (https://www.qualtrics.com/uk/), with participants randomly allocated using simple randomization to one of these six conditions. All data were collected on 24 March 2021.

#### Sample

A total of 1,592 participants were recruited through Prolific (https://www.prolific.co/) (Mean age = 36.47, s.d. = 13.00; % women = 50%, *n* = 795), with 34% (*n* = 538) indicating that they had donated blood previously and 12.1% (*n* = 194) being current donors (donated within the past 2 yr). There were 266 participants in the BAU/control, 273 in the ‘identity-prime-only’, 263 in the ‘warm-glow-only’, 256 in the ‘warm-glow-plus-identity’, 279 in the ‘impure-altruism-only’ and 255 in the ‘impure-altruism-plus-identity’ conditions (see Supplementary File 7 for sampling strategy and details on sample bias).

#### Power calculation

A Cohen’s *d* of 0.344 for positive ratings of warm-glow vs altruistic messages for blood donation was previously recorded^66^. Achieving 80% power to detect a small effect with *α* = 0.05 for a 2 (warm glow: yes, no) × 2 (identity: yes, no) design requires 265 participants per condition. For a series of pairwise comparisons of experimental groups, detecting a small effect with a Cohen’s *d* of 0.344 requires 135 participants per condition.

#### Outcome measures

The following measures were assessed.

##### Afforded donor vs recipient focus

This was calculated as the average of 2 items (“To what extent do you think that the message focuses on the blood donor, the recipient of blood or both?” and “To what extent do you think that the message focuses on the emotions of the blood donor, the recipient of blood or both?”) measured on a scale from −50 (primarily on the blood donor) to +50 (primarily on the recipient of blood) with a midpoint of 0 (equally on both the donor and recipient).

##### Afforded warm glow

This was calculated as the sum of 2 items (“The message makes me feel that donating blood is personally rewarding in itself.” and “The message makes me feel that donating blood would make me feel like a good person.”) measured on a 7-point scale (1 = not at all, to 7 = completely).

##### Afforded focus on maintaining blood supply

This was assessed with a single item (“The message makes me feel that donating blood would ensure that there is enough blood for all who need it.”) measured on a 7-point scale (1 = not at all, to 7 = completely).

#### Pre-registration

This experiment was pre-registered (https://osf.io/q4c6v/). Pre-registered predictions are listed in Table 1.

### Statistical analyses for Studies 1–6

All data were analysed using standard statistical packages (IBM SPSS v.26 and 27, ZumStat, Psychometrica, Stata 17, M*Plus* 8.4, PROCESS 4.0, Comprehensive Meta-Analysis 2). All tests were two-tailed. All effect sizes for all analyses are reported using Cohen’s *d*. Cohen’s *d* was derived for comparison across multiple-group means using the procedures described in ref. ^94^ and from *Z* scores using the procedure described in ref. ^95^, with both implemented in Psychometrica^96^. ORs were converted to Cohen’s *d* using procedures described in refs. ^96,97^. Comparisons of percentages between groups, including interactions, used procedure detailed in refs. ^98,99^. Mediation models were run in M*Plus* and PROCESS^100^. Meta-analysis was conducted using Comprehensive Meta-Analysis (v.2)^97^. Our main analyses had dichotomous outcomes and were analysed using logistic models and non-parametric tests. When continuous variables were outcomes, we present boxplots (for example, afforded warm glow) or Q-Q plots (experienced warm glow, Study 5; see Supplementary File 9 and Table 11a,b). In all cases, there was evidence of non-normality. We analysed these data using analysis of variance (ANOVA) and ordinary least squares (OLS) regression models as Monte-Carlo studies show that these techniques are robust to non-normality especially when sample sizes are large (greater than 300 for ANOVA and 1,000 for OLS)^101,102^. All results reported are two-tailed with no adjustments for multiple comparisons made.

### Reporting summary

Further information on research design is available in the Nature Portfolio Reporting Summary linked to this article.

### Supplementary information

Supplementary Information Supplementary Figs. 1–8 and Tables 1–17 to support the main paper conclusions, plus details of sampling strategies and biases, development of the study materials and a review of methods to assess warm glow.

Reporting Summary

## Data Availability

All data supporting the findings of Studies 1–6 are available (https://osf.io/ps5kb/). Source data are provided with this paper.
