# Supplementary material for: Warming up cool cooperators
Source: Nat Hum Behav. 2023 Sep 14;7(11):1917–32. doi: 10.1038/s41562-023-01687-6 (PMC10663147; doi:10.1038/s41562-023-01687-6)
Supplement: Supplementary file 1 — Supplementary Figs. 1–8 and Tables 1–17 to support the main paper conclusions, plus details of sampling strategies and biases, development of the study materials and a review of methods to assess warm glow. [file 41562_2023_1687_MOESM1_ESM.pdf]

nature human behaviour

Article

https://doi.org/10.1038/s41562-023-01687-6

Warming up cool cooperators

In the format provided by the authors and unedited

Table of Contents

Supplementary File S1: Types of Evidence Supporting Warm-Glow 3
Assessing Warm-Glow: Behavioural and Experienced Warm-Glow 3
Behavioural Warm-Glow: Experimental Approach 3
Behavioural Warm-Glow: Survey Approach 4
Experienced Warm-Glow 4
Supplementary File S2: Field-Based Experiment, Treatment Messages and Marginal Effects ... 6
Structure of the Treatment Messages 6
Active Ingredients of Messages and the Rank Order of Effectiveness 8
Field-Based Experiment: Messages 9
Univariate Comparisons for Study 1 13
Margins analysis for Study 1 15
Supplementary File S3: Implementation Analyses 17
Implementation analyses sensitivity analysis: Adjusted aggregate rates adjusted for aggregate age and sex. 19
Supplementary Files S4 for Studies 3 to 5: Experienced Warm-Glow and Booking Status 22
Study 3: Association between warm-glow and booking status 22
Study 3: Identifying markers of anticipated warm-glow 22
Study 3: Sample Selection Effects 23
Study 4: Associations between booking status from warm-glow 25
Meta-Analysis of the Association Between Warm-Glow and Booking Status Across Studies 3, 4 and 5 27
Supplementary Files S5: Predicting Blood Donor Behaviour Across Studies 4 and 5 28
Predicting Blood Donor Behaviour Study 4 28
Predicting Blood Donor Behaviour Study 5 29
Supplementary Files S6 for Study 6: Treatment Message Active Ingredients 31
Summary of Key Findings 31
Box-Plots for the Six Experimental Conditions 31
Warm-Glow By Identity Interactions 34
Moderation by Donor Status 37
Planned Contrasts 39
Supplementary Files S7: Sampling Strategies and Sample Bias 41
Sample Bias and Randomization Checks for Study 1: Field-Based Experiment 41
Sampling Strategy and Sample Bias Study 3: Booking Another Appointment, Warm-Glow and Pure Altruism 42
Sampling Strategy and Sample Bias Study 4: Warm-Glow, Booking Another Appointment and Donor Status and Type 42
Sampling Strategy and Sample Bias Study 5: Temporal Stability of Warm-Glow Following Booking Another Appointment 43

Sampling Strategy and Sample Bias for Study 6: Message Validation 44

Supplementary Files S8: Timeline for Field-Based Experiment (Study 1) and the Implementation Analysis (Study 2) **45**

Supplementary Files S9: Q-Q Plots for Study 5 .... **46**

References **47**

Supplementary File S1: Types of Evidence Supporting Warm-Glow

Assessing Warm-Glow: Behavioural and Experienced Warm-Glow

In this section, we briefly detail the types of methods (experiments and psychometrics) that have been used to assess warm-glow. We split these into behavioural warm-glow and experienced warm-glow. Ferguson and Flynn (2016) distinguished between warm glow as behavioural preference observed in economic games and experienced warm-glow, defined as the positive affect associated with giving, and assessed via psychometric self-reports.

Behavioural Warm-Glow: Experimental Approach

The theory says that pure-altruists are motivated entirely by the outcome while warm-glow giving is independent of the outcome. Therefore, motivation based on pure-altruism can be crowded-out by a source of external funding. Take, for example, a charity. Suppose a government decides to pledge a certain amount of financial resources to a charity. In that case, the pure-altruist – whose motivations are driven by the outcome – will reduce their own contribution by the same amount as the Government has pledged, as the outcome can now be achieved through external funding. Their contribution will no longer make a difference to the outcome. The pure-altruist’s contribution is crowded out. On the other hand, for the warm-glow giver, charitable behaviour should not be crowded out as they are motivated by the act of giving itself and not the outcome per se. So even in the presence of an external source crowding out their contribution, the warm-glow giver will continue to donate. This idea of crowding out is the basis of behavioural warm-glow. This is operationalized experimentally as follows. Participants play a charity dictator game. Here the participant can choose a charity of their choice and decide to allocate some resources (monetary endowment) to that charity. In the warm-glow version of the charity dictator game participants are told that they can give some, none or all of their endowment to their charity. However, their donations are subject to one-to-one crowding out. That is, participants are told that their charity has, for example, an initial endowment of £10 and that for every £1 that they transfer to their charity, their charity lose £1 from that £10 initial endowment. Thus, regardless of the participant's actions, the charity always has £10. Therefore, the act of donating money does not affect the outcome. Under these circumstances, the pure-altruist should not give anything, but the warm-glow giver should continue to give. This is the basic set-up to test the theory of warm-glow developed by Crumpler and Grossman (2008) and has been the basis of many subsequent studies. The results from Crumpler and Grossman (2008) show extensive behavioural warm-glow giving, with 57% of subjects making a positive donation with a mean allocation of \$2.08 (out of a possible \$10). This has been replicated by Ferguson and Flynn (2016).

Behavioural Warm-Glow: Survey Approach

Carpenter (2021) has developed and validated a simple survey item that can be used to differentiate people who have a preference for pure-altruism or warm-glow giving. The survey item is as follow:

> "Think about the last time you gave to charity before today. What was most important to you
> (i) the total amount given by everyone,
> (ii) the amount that you personally gave or
> (iii) some other aspect of giving?"

Endorsing (i) indicates pure-altruism, (ii) warm-glow giving and (iii) another preference or reason.

Carpenter (2021) ran a field-based experiment whereby people were randomised to one of four treatments to buy a ticket to support a local charity whereby: (1) all the money given went to a charity or (2) 75% to the charity with 25% going to a lottery for all those who bought a ticket, or (3) 50% to the charity and 50% to the lottery or (4) 25% to the charity and 75% to the lottery. Carpenter shows that those classified as warm-glow givers using the behavioural survey item give more as more money goes to the charity and pure-altruists gave less as more money goes to the charity.

Experienced Warm-Glow

Evidence that people emotionally experience warm-glow from giving is supported by neuroscientific, psychometric, survey and experimental data.

Neuroscience: In terms of the neuroscience evidence, Moll et al. (2006) using a charity dictator game, showed increased activity in the ventral striatum, a reward centre associated with the control of dopamine, when subjects receive money for themselves but also similar increases when subjects give money to charity. Harbaugh et al. (2007) examined neural activity when people made charitable donations of pre-determined sizes from the subject's endowments, when they could either voluntarily choose to accept or reject various charitable donations or during mandatory trials when equivalently sized donations were imposed. Voluntary donations produced significantly higher activity in the ventral striatum than comparable mandatory donations and greater self-reported satisfaction.

Psychometric and Survey: Psychometrically, experienced warm-glow has been assessed in terms of items measuring satisfaction, reward, pleasure, feeling good, feeling emotionally positive, and feeling warm-glow having donated or helped someone (Evans & Ferguson, 2014; Ferguson & Flynn, 2016; Hartmann, Eisend, Apaolaza & D'Souza, 2017; Van Laden, 2018; Sweegers, Twisk, Quee, Ferguson & van den Hurk, 2021: see Table 1 from Hartmann et al., 2017 for a summary). Hartmann et al. (2017) provide the following definition of how to assess experienced warm-glow when referring to pro-environmental behaviour:

> "To correctly assess and differentiate warm glow experiences from other emotional responses, the measurement items composing the scale should

explicitly relate to the specific proenvironmental behavior potentially evoking warm glow. That is, the measure ought to establish a connection between the emotions elicited and the particular proenvironmental behavior in question." (p 47).

You could transpose the term pro-environmental for any other prosocial cooperative behaviour. The key take-home message is that the positive affective responses have to be directly linked to cooperative behaviour. Warm-glow can also be assessed using indices of intrinsic motivation (Hartmann et al., 2017; Van Laden, 2018; Clark, Kotchen & Moore, 2003; Taufik, Bolderdijk & L. Steg, 2015; Ferguson, Murray & O'Carroll, 2019) and the intrinsic regulation from self-determination theory (SDT) has been directly linked to assessing warm-glow (Ferguson, Murray & O'Carroll, 2019). Indeed, survey data shows that these measures of warm-glow are associated with prosociality and cooperation (e.g., Ferguson et al., 2008; Hartmann et al., 2017; Van Laden, 2018).

Psychometrics and Experimental. Some studies have examined the association between experienced and behavioural warm-glow. Konow (2010) showed that the amount of resources donated were positively associated with experienced warm-glow when the focus of a dictator games was on a charity, but not when the focus was on a stranger. Ferguson and Flynn (2016) also showed that experienced warm-glow was directly proportional to the amount of resources allocated to a charity using a standard warm-glow charity dictator game, whereby people can choose to donate some, none or all of their endowment to a charity with that donation crowded out at a ratio of one-to-one. However, Ferguson and Flynn (2016) also include a condition whereby people could take money from the charity. This take option reduced behavioural warm-glow, compared to the standard warm-glow charity dictator game, where the only option is to give. However, those in the take option reported higher levels of experienced warm-glow than those in the standard warm-glow charity dictator game. The take option highlighted that doing nothing, neither giving nor taking, meant that the charity neither gains nor loses and as such, prospers. Thus, the elevated warm-glow in the take condition shows that warm-glow is not just about the amount of help or resource given but also about doing the morally right thing. Therefore, we can argue that behavioural and experienced warm-glow are linked with experienced warm-glow associated with ensuring that the morally right thing line of action is taken.

Ferguson et al. (2012) showed that blood donors, compared to non-donors, gave more on a warm-glow charity dictator game, and less on a standard charity dictator game. Also feelings of warm-glow are associated with giving in a standard charity dictator game, especially for blood donors.

Supplementary File S2: Field-Based Experiment, Treatment Messages and Marginal Effects.

This section details supporting information for the field-based experiment.

Structure of the Treatment Messages

Supplementary Table 1 below details the structure of the treatment messages to reflect the theoretical nature of warm-glow and impure-altruism. All messages contain references to warm-glow or positive affect (warm-glow) due to donation and the expectation of a future reward by experiencing that positive affect again by donating blood again. The warm-glow messages contain no reference to the recipient of blood or the blood supply. In contrast, impure altruism-messages contain a reference to recipients of blood and how their donation has helped others. The warm-glow and impure-altruism messages that include an additional focus on the identity as donor contain an extra phrase emphasising this that is not present in the warm-glow only or impure-altruism only messages. In our pre-registration of study 1 (https://osf.io/5m69k: Field-Based Experiment in the main text) we referred to the warm-glow messages as generic warm-glow and the impure-altruism-messages as reactivated warm-glow.

We initially used the term reactivated warm-glow as the messages also included the opening phrase: “Most people can’t remember what they were doing 6 weeks ago…”. We initially thought that this would make the donor reflect more on their action as a donor and that it may enhance a sense of nostalgia for having helped others and in so doing again reactivate feelings of warm-glow. However, on reflection these messages signal impure-altruism as they afford both the warm-glow that the donor feels and have a clear focus on helping recipients, which is central to the idea of impure-altruism (Supplementary Table 1). This reconceptualization was borne out in the analyses conducted for study 6.

As part of study 6 we also assessed whether in fact there was any heightened sense of nostalgia associated with the messages using the follow question: “The message would make me feel nostalgic about how I felt about my previous blood donation”. Comparing the messages on the nostalgia question there was no significant effect ($F_{(3, 1049)} = 0.963$, $P = 0.409$, $\eta_p^2 = .003$). The mean for warm-glow was 4.48 (95% C.I. = 4.27, 4.68, n = 263), for warm-glow-plus-identity the mean was 4.43 (95% C.I. = 4.21, 4.65, n = 256), for impure-altruism the mean was 4.58 (95% C.I. = 4.38, 4.77, n = 279), and impure-altruism-plus-identity the mean was 4.66 (95% C.I. = 4.47, 4.86, n = 255). As such, there is no evidence that nostalgia varies across the messages and, therefore, would have no bearing on the effectiveness of the messages.

		**Treatment Message**			
		**Simple Warm-Glow**	**Simple Warm-Glow Plus Identity**	**Impure-Altruism**	**Impure-Altruism Plus Identity**
**Active Ingredient**	**Donor focus on warm glow**	*... the warm glow that comes with donating blood…*	*... the warm glow that comes with donating blood…*	*…you felt good as your gift of blood…*	*…you felt good as your gift of blood…*
	**Other-Regarding Recipient Focus**			*…helping to save lives and bring happiness to grateful patients and their families…*	*…helping to save lives and bring happiness to grateful patients and their families…*
	**Future Expectation of Reward from Warm-Glow**	*The good news is in a few weeks you'll be ready to donate again, so you'll be able to experience this good feeling again soon*	*The good news is in a few weeks you'll be ready to donate again, so you'll be able to experience this good feeling again soon*	*The good news is in a few weeks you'll be ready to donate again, so you'll be able to experience this good feeling again soon*	*The good news is in a few weeks you'll be ready to donate again, so you'll be able to experience this good feeling again soon*
	**Identity**		*..was the day you became a blood donor…*		*..was the day you became a blood donor…*

Supplementary Table 1: *Theoretical structure of the treatment messages*

.

Active Ingredients of Messages and the Rank Order of Effectiveness

In terms of active ingredients, we hypothesise that the warm-glow afforded by a message in conjunction with a focus on reinforcing the donor’s identity will be the main active ingredient to enhance donor return. Thus, we use identity as a super-ordinate category to rank the potential effectiveness of the messages. On that basis, the “warm-glow-plus-identity” and “impure-altruism-plus-identity” should be more effective than the “warm-glow-only” and “impure-altruism-only” messages, leading to an initial rank-ordering below:

Superordinate Rank Order: [(WG+I & IA+I) > (WG & IA)]

However, it is possible to suggest a refinement to this initial rank ordering based on sub-ordinate ranking within the super-ordinate rank. This is based on the number of active ingredients. First, messages with an identity component will outrank those without an identity component (Superordinate Rank Order). Within messages with an identity component, the “impure-altruism” version has more active components than the “warm-glow” version, as the “impure-altruism” version also contains an other-regarding component that focuses on the recipients of blood. Based on a simple dose-response analogy, the impure-altruism messages should have more informational salience, as it has two active ingredients, generating a bigger impact on the donor and therefore be more effective than the “warm-glow” version. The same internal rank ordering should be observed between the “impure-altruism” and “warm-glow” messages that do not focus on identity. Thus, we would arrive at the potential rank-ordering below. This was the basic ordering proposed in our pre-registration.

Rank Order: IA+I > WG+I > IA > WG

The actual format of the messages as Australian Red Cross Lifeblood used them is shown below.

Field-Based Experiment: Messages

Impure Altruism Only

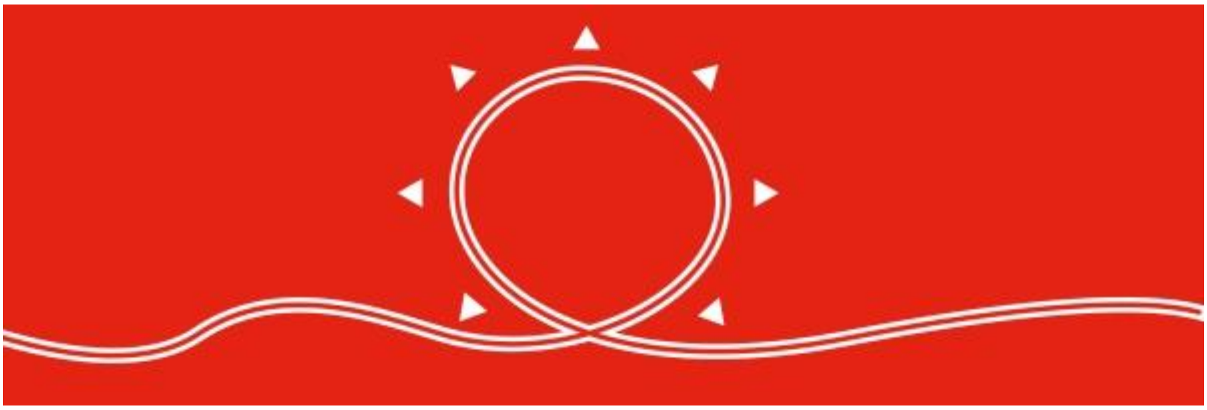

YOU'RE POPULAR!

Hi xxxx,

Most people can't remember what they were doing six weeks ago, but we hope you felt good as your gift of blood was helping to save lives and bring happiness to grateful patients and their families.

The good news is in a few weeks you'll be ready to donate again, so you'll be able to experience this good feeling again soon.

Your blood is especially needed because, like 40 per cent of Australians, you have type O positive blood. It can be given to 81 per cent of the population!

If you have any questions or would like to tell us something about your experience, we'd love to hear from you on **13 14 95** or contact us **online**.

Thanks for making a life-saving donation,

The Red Cross Blood Service Team

This image is copyright of Australian Red Cross Lifeblood ((Lifeblood) Formally known as Australian Red Cross Blood Service) and reproduced with permission.

Impure Altruism plus Identity

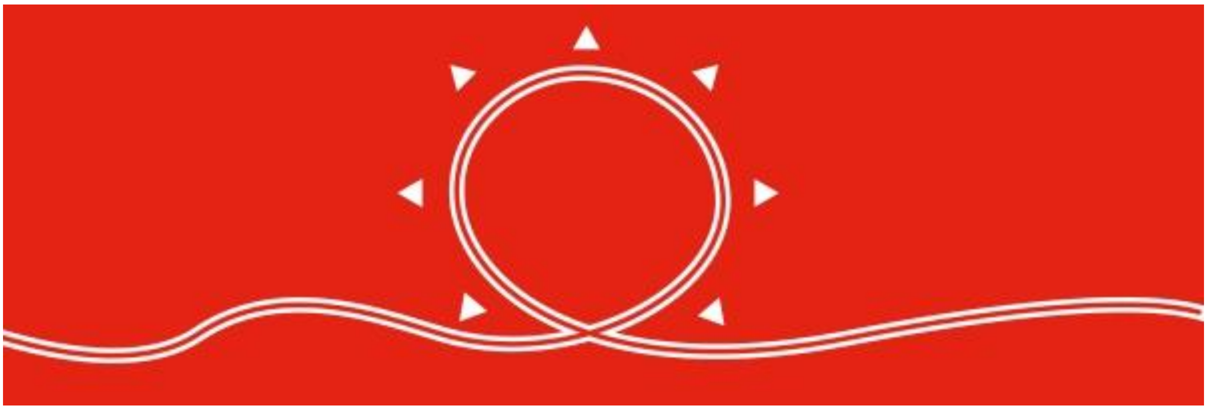

YOU'RE POPULAR!

Hi xxxx,

Most people can't remember what they were doing six weeks ago, but we wanted to remind you that this was the day you became a blood donor. We hope you felt good as your gift of blood was helping to save lives and bring happiness to grateful patients and their families.

The good news is in a few weeks you'll be ready to donate again, so you'll be able to experience this good feeling again soon.

Your blood is especially needed because, like 40 per cent of Australians, you have type O positive blood. It can be given to 81 per cent of the population!

If you have any questions or would like to tell us something about your experience, we'd love to hear from you on **13 14 95** or contact us **online**.

Thanks for making a life-saving donation,

The Red Cross Blood Service Team

This image is copyright of Australian Red Cross Lifeblood ((Lifeblood) Formally known as Australian Red Cross Blood Service) and reproduced with permission.

Warm-Glow Plus Identity

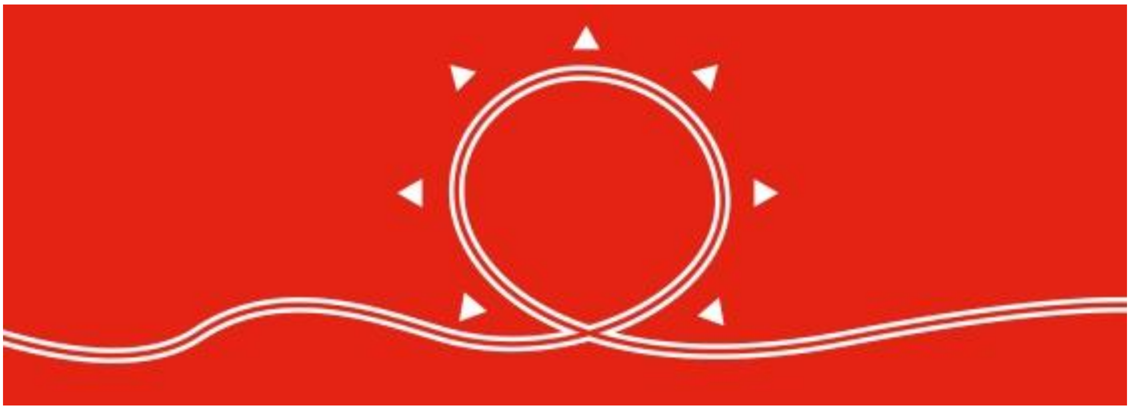

YOU'RE POPULAR!

Hi xxxx,

We hope you've been basking in the warm glow that comes with donating blood, because six weeks ago was the day you became a blood donor.

The good news is in a few weeks you'll be ready to donate again, so you'll be able to experience this good feeling again soon.

Your blood is especially needed because, like 40 per cent of Australians, you have type O positive blood. It can be given to 81 per cent of the population!

If you have any questions or would like to tell us something about your experience, we'd love to hear from you on 13 14 95 or contact us **online**.

Thanks for making a life-saving donation,

The Red Cross Blood Service Team

This image is copyright of Australian Red Cross Lifeblood ((Lifeblood) Formally known as Australian Red Cross Blood Service) and reproduced with permission.

Warm-Glow Only

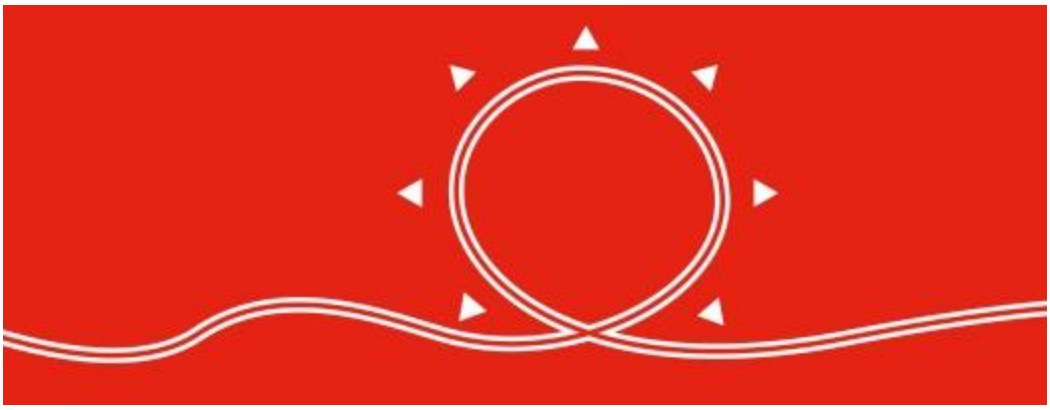

YOU'RE POPULAR!

Hi xxxx,

We hope you've been basking in the warm glow that comes with donating blood!

The good news is in a few weeks you'll be ready to donate again, so you'll be able to experience this good feeling again soon.

Your blood is especially needed because, like 40 per cent of Australians, you have type O positive blood. It can be given to 81 per cent of the population!

If you have any questions or would like to tell us something about your experience, we'd love to hear from you on **13 14 95** or contact us **online**.

Thanks for making a life-saving donation,

The Red Cross Blood Service Team

This image is copyright of Australian Red Cross Lifeblood ((Lifeblood) Formally known as Australian Red Cross Blood Service) and reproduced with permission.

Univariate Comparisons for Study 1

Supplementary Table 2 below provides the univariate comparisons for the four active treatment messages in Study 1 to the BAU control separately for those who booked another appointment and those who did not. As can be seen the only significant effect was for the warm-glow-plus-identity message among those who had not booked another appointment.

Treatment Comparisons	Did Not Book				Booked			
BAU	Z	P =	%Difference (95% CI)	Cohen's D	Z	P =	95% CI	Cohen's D
WG+I	2.111	0.0348	5.4 (10.413, 0.387)	0.112	1.827	0.0677	-6.0 (-12.386, 0.437)	0.125
WG	0.119	0.9052	-0.3 (-5.236, 4.636)	0.006	0.499	0.6179	1.6 (-4.686, 7.886)	0.034
IA+I	0.905	0.3657	2.3 (-2.684, 7.284)	0.047	1.709	0.0875	-5.6 (-12.024, 0.824)	0.117
IA	0.938	0.3483	2.4 (-2.616, 7.416)	0.049	1.547	0.1218	-5.0 (-11.334, 1.334)	0.104

Supplementary Table 2: *Individual Comparisons Across the Treatment Condition for those who did not book another appointment and those who booked from Study 1, relative to BAU condition.. These were Z tests for difference between proportion implemented in Zumastat 4.0. All analyses were two-tailed and no adjustments were made for multiple comparisons.*

Margins analysis for Study 1

Supplementary Table 3 below provides the full margin analysis for the interaction between treatment messages and booking status for Study 1 reported in Table 3 in the main text.

	Delta Method *dy/dx (std. err.)*	*z*	*P =*	*95% C.I.* *Lower*	*Upper*
**Treatment Message**					
Base: BAU					
**Warm-Glow-plus Identity**					
Did not Book	0.058 (0.025)	2.28	0.023	0.008	0.108
Booked	-0.060 (0.033)	-1.81	0.070	-0.124	0.005
**Warm-Glow-Only**					
Did not Book	-0.002 (0.025)	-0.09	0.928	-0.051	0.047
Booked	0.019 (0.032)	0.59	0.555	-0.044	0.083
**Impure-Altruism-plus-Identity**					
Did not Book	0.024 (0.025)	0.97	0.334	-0.025	0.074
Booked	-0.052 (0.033)	-1.60	0.110	-0.117	0.012
**Impure-Altruism-Only**					
Did not Book	0.026 (0.025)	1.02	0.308	-0.024	0.076
Booked	-0.042 (0.032)	-1.30	0.192	-0.106	0.021

Supplementary Table 3. *Margins for the intervention between booking status and Treatment Message Relative to the BAU control for Study 1. These were implemented using the margins command in Stata 17. All analyses were two-tailed and no adjustments were made for multiple comparisons*

Supplementary Table 4 below provides the margin analysis for the remaining effects reported in Table 3 in the main text for Study 1.

	Delta-method				***95% C.I.***	
	Margin	***std. err.***	***z***	***P =***	***Lower.***	***Upper***
**Sex**						
Male	0.489	0.011	44.76	<0.001	0.468	0.511
Female	0.482	0.009	54.77	<0.001	0.065	0.499
Treatment Message						
BUA	0.482	0.015	31.17	<0.001	0.452	0.512
WG+I	0.496	0.015	32.90	<0.001	0.466	0.525
WG	0.489	0.016	31.34	<0.001	0.458	0.519
IA+I	0.477	0.015	31.47	<0.001	0.447	0.506
IA	0.482	0.015	31.83	<0.001	0.452	0.511
Booking Status						
Did not Book	0.395	0.008	47.96	<0.001	0.379	0.411
Booked	0.632	0.010	60.30	<0.001	0.612	0.653
Blood Group						
A-	0.502	0.027	18.61	<0.001	0.449	0.555
A+	0.475	0.011	42.31	<0.001	0.453	0.497
O-	0.543	0.020	26.13	<0.001	0.503	0.584
O+	0.477	0.010	47.41	<0.001	0.457	0.497

Supplementary Table 4: *Margins for Sex, Treatments, Booking Status and Blood Group on Return Behaviour. BAU = Business as Usual, WG+I = Warm-Glow-Plus-Identity; WG = Warm-Glow-Only; IA+I = Impure-Altruism-Plus-Identity; IA = Impure-Altruism-Only. B Coefficients are Unstandardized Coefficients. These were implemented using the margins command in Stata 17. All analyses were two-tailed and no adjustments were made for multiple comparisons.*

Supplementary File S3: Implementation Analyses

Supplementary Figure 1 below plots the data showing the change in first-time donor returns for those who booked and those who had not booked another appointment across the three pre-implementation time window and the two post-implementation time windows.

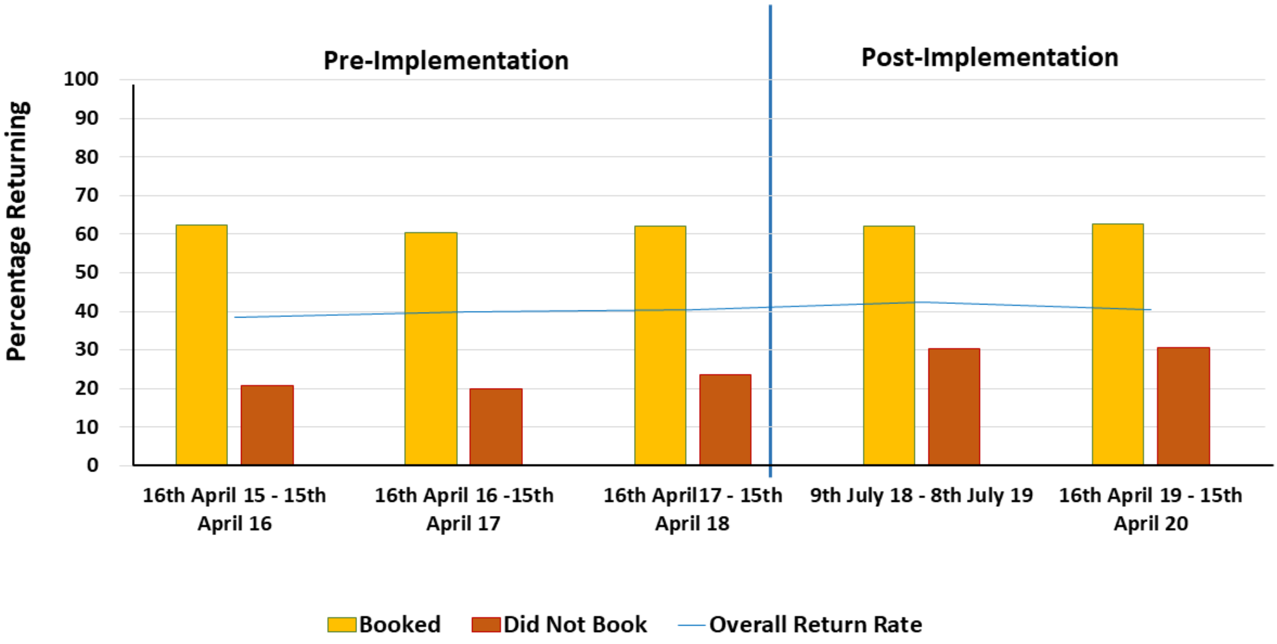

Supplementary Figure 1: ***Donor Return Rates Pre- and Post-Implementation For Those who Booked and Those Who Did Not.*** *The point estimate for the percentage return rate for those who booked was 62.23 (95%CI = 61.75, 62.71) for pre-implementation for April 15 to April 16 (n = 24086/38677), was 60.45 (95% CI = 60.00, 60.89) for pre-implementation for April 16 to April 17 (n =27670/45777), was 61.94 (95% CI = 61.47, 62.45) for pre-implementation for April 17 to April 18 (n = 23676/38227), was 62.19 (95% CI = 61.65, 62.73) for Immediate Post July18 to July 19 (n = 19341/31098), and 62.52 (95% CI = 61.95, 63.10) for 2nd Post Implementation April 19 to April 20 (n = 17131/27402). The point estimate for the percentage return rate for those who did not booked was 20.85 (95% CI = 20.50, 21.20) for pre-implementation for April 15 to April 16 (n = 10769/51640), was 20.00 (95% CI = 19.64, 20.36) for pre-implementation for April 16 to April 17 (n = 9529/47653), was 23.65 (95% CI = 23.27, 24.03) for pre-implementation for April 17 to April 18 (n = 11440/48379), was 30.38 (95% CI = 29.98, 30.78) for Immediate Post July 18 to July 19 (n = 15392/50668), and 30.69 (95% CI = 30.32, 31.06) for 2nd Post Implementation April 19 to April 20 (n = 18764/61149). The point estimate for the percentage overall return rate was 38.57 (95% CI = 38.25, 38.89) pre-implementation for April 15 to April 16 (n = 34837/90317), was 39.81 (95% CI = 39.50, 40.12) for pre-implementation mean for April 16 to April 17 (n = 37199/93430), was 40.55 (95% CI = 40.22, 40.88) for pre-implementation for April 17 to April 18 (n = 35116/86606), was 42.48 (95% CI = 42.14, 42.82) for Immediate Post July 18 to July 19 (n = 34733/81766), and 40.54 (95% CI = 40.22,40.86) for 2nd Post Implementation April 19 to April 20 (n = 35895/88551). Analyses were conducted using* procedures detailed in [96-97] *and implemented in ZumaStat 4.0. All analyses were two-tailed and no adjustments were made for multiple comparisons.*

The results in Supplementary Figure 1 reveal a significant (Z = 8.52, *P* < 0.001, D_{Cohen} = 0.04) increase from pre- (time window 16-17) to post-implementation (time window 19-20) in overall return rate. However, as clearly shown in Figures 1, the effect was largely due to the post-implementation increase in return rates among those who had not booked initially.

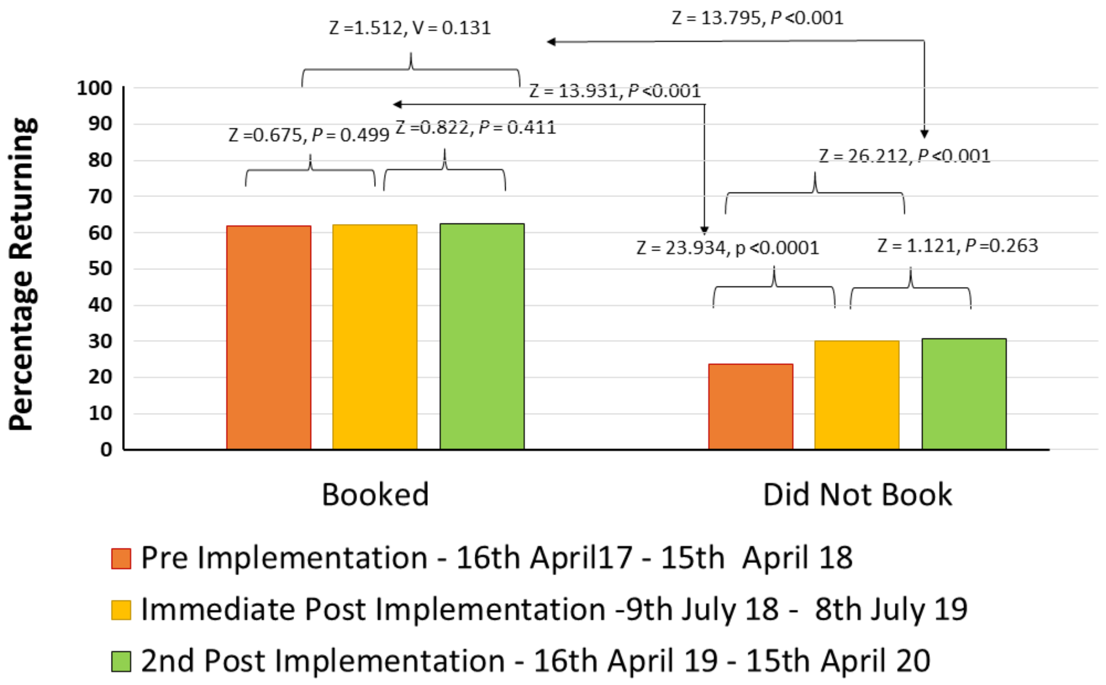

Supplementary Figure 2: ***Donor Return Rates Immediately Pre- and Post-Implementation For Those who Booked and Those Who Did Not***. *The point estimate was 61.94 (95% CI = 61.45, 62.43) for pre-implementation for April 17 to April 18 (n = 23676/38227), was 62.19 (95% CI = 61.65, 62.73) for Immediate Post July18 to July 19 (n = 19341/31098), and 62.52 (95% CI = 61.95, 63.10) for 2nd Post Implementation April 19 to April 20 (n = 17131/27402). The point estimate was 23.65 (95% CI = 23.27, 24.03) for pre-implementation for April 17 to April 18 (n = 11440/48379), was 30.38 (95% CI = 29.98, 30.78) for Immediate* Post July 18 to July 19 (n = 15392/50668), and 30.69 (95% CI = 30.32, 31.06) for 2nd Post Implementation April 19 to April 20 (n = 18764/61149). There was no statistically significant effect on return rates among those who had booked ($Z_{\text{pre vs 1st post}}$ = 0.675, *P* = 0.499, % difference 0.250% [95%CI = -0.476, 0.976]; and $Z_{\text{pre vs 2nd post}}$ = 1.512, *P* = 0.131, % difference 0.580% [95% CI = -0.172, 1.332]). There was a significant increase in return among those who had not booked ($Z_{\text{pre vs 1st post}}$ = 23.934 *P* < 0.001; % difference 6.73% [95%CI = 6.179, 7.281]; and $Z_{\text{pre vs 2nd post}}$ = 26.212, *P* < 0.001, % difference 7.04% [95%CI = 6.514, 7.566]). There is a significant interaction between booking status (booked, did not book) and pre- and post-implementation time windows with the increase in donor returns significantly greater for those who had not booked, compared to those who had booked. This was seen for the window immediately prior to implementation with (i) the 1st post-implementation time window (Z = 13.931. *P* < 0.001, % difference 6.48 [95% CI = 5.568, 7.392]) and (ii) and the 2nd post-implementation time window (Z = 13.795, P < 0.001, % difference 6.46 [95% CI = 5.542, 7.378]).Analyses were conducted using Z tests for proportion following procedures detailed in the main text [98-99] and implemented in ZumaStat 4.0. All analyses were two-tailed and no adjustments were made for multiple comparisons.

Supplementary Figure 2 compares the time-window just prior to implementation (16th April 2017 - 15th April 2018) to each of the two post-implementation windows (1st post: 9th July 2018 - 8th July 2019; and 2nd post: 16th April 2019 - 15th April 2020). The results presented in Supplementary Figure 2 show no statistically significant effect on return rates among those who had booked ($Z_{\text{pre vs 1st post}} = 0.675$, $P = 0.499$,% difference 0.250% [95%CI = -0.476, 0.976] $D_{Cohen} = 0.005$; and $Z_{\text{pre vs 2nd post}} = 1.512$, $P = 0.131$, % difference 0.580% [95% CI = -0.172, 1.332]; $D_{Cohen} = 0.012$), but a significant increase in return among those who had not booked ($Z_{\text{pre vs 1st post}} = 23.934$ $P < 0.001$; % difference 6.73% [95%CI = 6.179, 7.281]; $D_{Cohen} = 0.153$ and $Z_{\text{pre vs 2nd post}} = 26.212$, $P < 0.001$. % difference 7.04% [95%CI = 6.514, 7.566]; $D_{Cohen} = 0.159$). We also observed significant interactions between booking status (booked, did not book) and pre- and post-implementation time windows (Supplementary Figure 2). Such that, the increase in donor returns was significantly greater for those who had not booked, compared to those who had booked, when comparing the time window immediately prior to the implementation with (i) the 1st post-implementation time window ($Z = 13.931$. $P < 0.001$, % difference 6.48 [95% CI = 5.568, 7.392]; $D_{Cohen} = 0.065$) and (ii) and the 2nd post-implementation time window ($Z = 13.795$, $P < 0.001$, % difference 6.46 [95% CI = 5.542, 7.378]; $D_{Cohen} = 0.066$: Supplementary Figure 2).

Thus, exposure to the warm-glow-plus-identity message significantly increased return rates among those who had not booked another appointment but had no significant effect on return rates among those who had booked. This resulted in an extra 3,409 donations from those who had not booked from the time window immediately before implementation to the first post-implementation time window and 4,304 in comparison to the second post-implementation time window.

Implementation analyses sensitivity analysis: Adjusted aggregate rates adjusted for aggregate age and sex.

To examine the effects of age and sex on the aggregate results presented in Figure 3 we re-ran the aggregated analysis on percentage return rates adjusted for aggregate age and sex (percentage female). We obtained estimates of age- and sex-adjusted percentage return rates by applying 2 (Implementation: pre-implementation [3 periods] vs post-implementation [3 periods] by 2 (Booking status: booked vs did not book) general linear model on aggregate return rates with aggregate age and sex (percentage female) as covariates. The observed percentage return rates for the ten cells are detailed in the legend of Supplementary Figure 1 and the age-adjusted and sex-adjusted percentage return rates for those who booked were 59.488 in the pre-implementation period and 57.500 in the post-implementation period. For those who had not booked they were 27.113 in the pre-implementation period and 30.078 in the post-implementation period. Analyses showed that there was a significant effect for those who booked with a -1.98% decrease ([95% CI = -2.474, -1.502], $Z = 8.022$, $P < 0.001$), a significant 2.93% increase for those who did not booked ([95% CI = 2.586, 3.290], $Z = 16.374$, $P < 0.001$) and a significant interaction between booking status and implementation period ($Z = 16.100$, $P < 0.001$) such that the increase in those who booked was significantly greater than the small decrease in those who did not. Thus, this simple adjustment for age and sex does not alter the pattern of results in terms of the key interaction and the effect of the warm-glow-plus-identity message on return rates for those who do not book. We report the raw non-adjusted values in the main paper as the effect of these covariates does not alter the key hypothesised effects.

Calculation of Extra Donors Due to Implementation of the “Warm-Glow-plus-Identity” Message: On average, there was a 6.705% increase in donations from those who did not book from the immediate pre-implementation period to the two post-implementation periods (6.73% [95% CI 6.179, 7.281] and 7.04% [95% CI 6.515, 7.566]), which is set against 1.93% and a -0.01% change in overall return rates. The 6.73% increase from the immediate pre-intervention window to the final post-intervention window among donors who had not re-booked (the change observed from 16th April 2017 - 15th April 2018 to 9th July 2018 - 8th July 2019) equated to an extra 3,409 donations attributable to the warm-glow intervention. This gave a number needed to treat (NNT) of 15, indicating that for every 15 donors who had not booked and were not expected to return, exposure to the warm-glow message encouraged one to return. The 7.04% increase in returns among those who had not booked (the change from 16th April 2017 - 15th April 2018 versus 16th April 2019 - 15th April 2020) equated to an extra 4,304 donations attributable to the warm-glow intervention. This gave an NNT of 14. Among donors who booked, the increase in the number of donors returning between immediate pre-intervention and first post-intervention windows attributable to the warm-glow intervention was 78, with an increase of 159 in total donations between the immediate pre-intervention and second post-intervention windows.

Supplementary Table 5 below provides the details of the number of first-time donors and those who booked or did not book that underlie the implementation study (Study 2).

	Pre-Implementation			Post-Implementation	
	16th April 2015 - 15th April 2016.	16th April 2016 - 15th April 2017.	16th April 2017 - 15th April 2018.	9th July 2018 - 8th July 2019	16th April 2019 - 15th April 2020.
Number of first-time donors	90,317	93,430	86,606	81,766	88,551
Booked	38, 677	45,777	38,227	31,098	27, 402
Did not Book	51, 640	47,653	48,379	50,668	61,149
**Follow Up Data**					
N who returned at 3 months	34,837	37,199	35,116	34,733	35,895
N, who returned at 3 months who had booked	24,068	27,670	23,676	19,341	17,131
N who returned at 3 months who had not booked	10,769	9,529	11,440	15,392	18,764

Supplementary Table 5. *Numbers of Donors in the Implementation Analysis Study 2 Aggregates: N booked pre-implementation 122681, N booked and donated pre-implementation = 75,414 (% donated = 61.47%), N did not book pre-implementation 147672, N did not book and donated pre-implementation 31,738 (% donated = 21.49%), N booked post-implementation 58500, N booked and donate post-implementation = 36472 (% donated = 62.35%), N did not book post-implementation 111817, N did not book and donated post-implementation 34,156 (% donated = 30.55%).*

Supplementary Files S4 for Studies 3 to 5: Experienced Warm-Glow and Booking Status

This section provides additional supporting data for studies 3 to 5 on the links between booking status, self-reported warm-glow and return behaviour.

Study 3: Association between warm-glow and booking status

This study examines the association between warm-glow, pure-altruism and booking status during the COVID-19 pandemic.

Study 3: Identifying markers of anticipated warm-glow

We applied principle-axis-factor analysis (PAF) with oblique rotation to the 12 adjectives that focused on the donor's next donation during the COVID-19 pandemic in the sample of first-time donors (Supplementary Table 6). All donors provided complete data (n = 716). There were 3 Eigenvalues greater than unity (3.179, 1.859, 1.161), accounting for 56.161% of the variance. The Scree test and a Parallel Analysis indicated that a 3 -factor solution was the optimal solution (Supplementary Table 6). Adjectives with loadings greater than 0.400 indicated the specific factor that the adjective contributed to. A good simple structure was achieved with no substantive cross-loadings (Ferguson & Cox, 1993).

	Factor		
	Anticipated Warm-Glow	Anticipated Negative Affect	Anticipated Calm
Satisfying	**.743**	.055	-.096
Rewarding	**.714**	-.023	.017
Pleasing	**.574**	.070	-.306
Positive	**.569**	-.191	-.040
Worthwhile	**.542**	-.071	.134
Pointless	.006	**.812**	-.098
Negative	.007	**.624**	.131
Unrewarding	-.051	**.498**	-.122
Unsatisfying	-.017	**.493**	.067
Displeasing	-.095	**.456**	.101
Relaxing	.235	.121	**-.612**
Stressful	.063	.282	**.507**
Coefficient α	***.77***	***.68***	***.50***
Mean (*Sd*)	18.65 (1.95)	5.33 (1.33)	6.32 (1.28)

Supplementary Table 6. *Oblique rotated factor matrix for Study 3. Coefficients in bold indicates adjective loading above .400 on its target factor (n = 716)*

The first factor contains the positive adjectives that reflect the anticipated positive feelings, personal reward, and worth that represent ‘anticipated warm-glow’ as defined in other studies (van der Linden, 2018). The second factor represents anticipated negative emotions (‘Anticipated negative affect) and the third factor ‘Anticipated calm’. The 5 ‘anticipated warm-glow’ adjective were linearly combined to create a scale where high scores equate to greater ‘anticipated warm-glow’, as were the 5 adjectives for ‘anticipated negative affect’ with a high score equating to greater negative perceptions, and the two adjectives for ‘anticipated calm’ were a high score equates to perceptions of greater relaxation. The coefficient alphas for anticipated warm-glow and anticipated negative emotions indicated good reliability. However, the coefficient alpha for anticipated calm was low. As this scale has only 2 items the mean-inter-item correlation is a better index of reliability and should be between .2 and .4 (Ferguson & Cox, 1993) and for anticipated calm, it was .34

Pure-Altruism: The mean for the pure-altruism measure was 9.26 (*Sd* = 1.06), with a mean inter-item correlation of .35

Study 3: Sample Selection Effects

Supplementary Table 7 below contains the results from a Probit model and a Heckman Probit Selection Model. The Probit model replicated the findings in Panel A of Table 4 in the main text. In terms of the selection model the Likelihood Ratio test for independence of equations is non-significant (χ^2 (1) = 0.45, *P* = 0.504). This indicates that while the Rho is positive it is not significantly different from zero (indeed the 95% C.I.s cross zero) and as such the selection corrected estimates are no better an estimation of the associations of age, gender, anticipated negative-affect, anticipated calmness, anticipated warm-glow and pure-altruism with booking status than the Probit estimates.

	Probit Model					Probit Heckman Selection Model				
	Coef.	***Std. Err.***	***P =***	***95% C.I.s***		***Coef.***	***Std. Err.***	***P =***	***95% C.I.s***	
Age	0.0150	0.0033	<0.001	0.0085	0.0214	0.0182	0.0034	<0.001	0.0115	0.0249
Gender (Female)	0.1435	0.1032	0.164	-0.0588	0.3457	0.1385	0.0408	0.165	-0.0571	0.3342
Anticipated Negative Affect	0.0407	0.0449	0.365	-0.0473	0.1287	0.0351	0.0430	0.390	-0.0449	0.1151
Anticipated calmness	0.0088	0.0413	0.832	-0.0722	0.0898	0.0073	0.0373	0.846	-0.2486	-0.0226
Anticipated Warm-Glow	0.0491	0.0293	0.095	-0.0084	0.1066	0.0428	0.0290	0.141	-0.0142	0.0998
Pure Altruism	-0.0575	0.0476	0.227	-0.1508	0.0358	-0.0508	0.0459	0.268	-0.1408	0.0391
Constant	-1.5991	0.6922	0.021	-2.9559	-0.2423	-1.8335	0.6266	0.003	-3.0617	-0.6054
Selection										
Age						0.0179	0.0033	<0.001	0.0115	0.0244
Gender (Female)						0.0197	0.1027	0.848	-0.1817	0.2211
Covid Test						0.2245	0.1096	0.041	0.0096	0.4393
Constant						0.0723	0.1564	0.643	-0.2342	0.3789
/athrho						0.9581	2.1361	0.656	-3.2359	5.1377
rho						0.7402	09658		-0.9969	0.9999
n	716					716				
R^2	.03									

Supplementary Table 7. *Covid Test (0 = no test taken 1 = test taken), Gender (0 = male, 1 = female). Coefficients are Unstandardized Coefficients* (Study 3). *These were implemented using the probit and heckprobit commands in Stata 1. All analyses were two-tailed and no adjustments were made for multiple comparisons.*

Study 4: Associations between booking status from warm-glow

Predicting Warm-Glow

Study 4 showed that warm-glow (main text Table 4, Panel B) predicted who booked, with higher levels of warm-glow associated with a greater likelihood of booking independent of donor status or donation type. The analyses in Supplementary Table 8 show that warm-glow is associated only with gender (women report higher levels of warm-glow) and not with donor type, donor status, or interaction.

	B (Se)	***P=***	***95% C.I.***	
			Lower	***Upper***
Gender	1.532 (0.263)	<0.001	1.015	2.048
Age	0.019 (0.011)	0.094	-0.003	0.041
Donor Status	0.320 (0.480)	0.506	-0.623	1.262
Donation Type	0.391 (0.372)	0.294	-0.339	1.122
Donor Status x Donation Type	-0.040 (0.573)	0.944	-1.165	1.084
Constant	12.831 (0.490)	<0.001	11.868	13.793
R^2	.034			
n	1124			

Supplementary Table 8: *OLS Regression for Experienced Warm-Glow on Donor Status, Donor Type and Demographics Predicting Warm-Glow: Gender (male = 0, female = 1); Donor Status (first time = 0, novice = 1); Donation Type (Plasma = 0, Whole blood = 1); Booked (did not book another appointment = 0, booked = 1)* (Study 4). *These were implemented using the regress command in Stata 17. All analyses were two-tailed and no adjustments were made for multiple comparisons.*

Controlling for Sample Clustering

Study 4 had a sampling structure whereby donors were selected in person across three donor centres. To confirm the results presented in Table 4 (Panel B) in the main text, we re-ran those analyses using clustered standard errors to account for the sampling structure. The results predicting booking status are shown in Supplementary Tables 9 and 10 below. The results replicate those in Table 4 (panel B) in the main text, with a higher level of warm-glow predicting booking. There is an additional effect that plasma donors are more likely to book.

	B (Robust Se)	*P=*	*OR*	*D_{Cohen}*	*95% C.I.*	
Warm-Glow	0.066 (0.012)	<0.001	1.068	0.036	1.043	1.094
Gender	0.108 (0.157)	0.490	1.114	0.060	0.819	1.515
Age	0.001(0.006)	0.826	1.001	0.001	0.989	1.014
Donor Status	0.087 (0.23)	0.709	1.090	0.048	0.692	1.718
Donation Type	-0.216 (0.054)	<0.001	0.806	-0.119	0.725	0.895
Donor Status x Donation Type	0.273 (0.328)	0.405	1.314	0.151	0.691	2.497
Constant	-0.206 (0.382)	0.589	0.813	-0.114	0.385	1.719
R^2	.020					
n	1,124					

Supplementary Table 9: *Logistic Regression for Booking Status on Donor Status, Donor Type and Warm-Glow to Predict Booking. Gender (male = 0, female = 1); Donor Status (first time = 0, novice = 1); Donation Type (Plasma = 0, Whole blood = 1); Booked (did not book an appointment = 0, booked = 1). Clustered Standard Errors*. (Study 4). *Exact p-value for warm-glow is* P = *$3.593e^{-08}$ and for Donation type P = .00006. These were implemented using the regress command in Stata 17. All analyses were two-tailed and no adjustments were made for multiple comparisons.*

	B (Robust Se)	*P=*	*95% C.I.*	
			Lower	*Upper*
Gender	1.532 (0.021)	<0.001	1.440	1.623
Age	0.019 (0.018)	0.403	-0.057	0.097
Donor Status	0.320 (0.140)	0.148	-0.288	0.918
Donation Type	0.391(0.313)	0.338	-0.955	1.737
Donor Status x Donation Type	-0.040 (0.333)	0.915	-1.471	1.391
Constant	12.831 (0.311)	0.001	11.490	14.171
R^2	.035			
n	1,124			

Supplementary Table 10: *OLS Regression on Donor Status, Donor Type and Demographics Predicting Warm-Glow: Gender (male = 0, female = 1); Donor Status (first time = 0, novice = 1); Donor Type (Plasma = 0, Whole blood = 1); Booked (did not book an appointment = 0, booked = 1). Clustered Standard Errors.* (Study 4). *These were implemented using the regress command in Stata 17. All analyses were two-tailed, and no adjustments were made for multiple comparisons*

Meta-Analysis of the Association Between Warm-Glow and Booking Status Across Studies 3, 4 and 5

Using the measures of warm-glow as the outcome we extracted the means, SDs and Ns for those who booked and those who did not for studies 3 and 4, and the means, SDs, Ns for experienced warm-glow at wave 1 and wave 2 for those who had booked and those who had not for study 5, as well as the association between warm-glow and waves 1 and waves 2 and the F ratio for the main effect of wave. Based on these, we conducted a meta-analysis across studies 3, 4, and 5 using Comprehensive Meta-Analysis Version 2, estimating both random and fixed effects models. The results of the random effects model showed that there was an overall significant positive effect across the three studies (standard difference in means = 0.209, standard error = 0.052, 95%CI = 0.106, 0.312, $P < 0.001$Cohen's $d = 0.201$, $r = 0.100$), the same pattern of results was observed for the fixed effects model standard difference in means = 0.212, standard error = 0.040, 95%CI = 0.134, 0.291, $P < 0.001$ Cohen's $d = 0.221$, $r = 0.103$). The Q of 3.387 (df = 2), $P = 0.184$ indicated no significant variation across the three studies and the I^2 of 40.94% indicated a moderate amount of heterogeneity not due to sampling error. The Forest plot is presented below (Supplementary Figure 3). Thus, there is a consistent positive association between reported warm-glow and booking-status"

Meta-Analysis and Forest Plot for Studies 3, 4 and 5

Model	Study name	Outcome	Std diff in means	Standard error	Variance	Lower limit	Upper limit	Z-Value	p-Value
			Statistics for each study						
	Study3	Booked vs Did Not Book	0.140	0.076	0.006	-0.009	0.289	1.844	0.065
	Study4	Booked vs Did Not Book	0.306	0.065	0.004	0.178	0.433	4.696	0.000
	Study5	Booked vs Did Not Book	0.167	0.069	0.005	0.032	0.302	2.424	0.015
Fixed			0.212	0.040	0.002	0.134	0.291	5.287	0.000

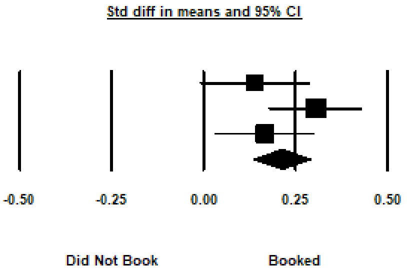

Supplementary Figure 3. *Forest Plot for the Association Between Warm-Glow and Booking Status Across Studies 3, 4 and* 5. For study 3, 294 booked and 422 did not book. For study 4, 781 booked and 343 did not book. For study 5, 612 booked and 320 did not book. Error bars are 95% C.I.s

Supplementary Files S5: Predicting Blood Donor Behaviour Across Studies 4 and 5

The section below details the results from Studies 4 and 5 predicting return attendance to donate as a function of booking status, warm-glow, demographics and donor characteristics.

Predicting Blood Donor Behaviour Study 4

The results presented in Supplementary Table 11 below show that both warm-glow and booking predict donor return behaviour.

					95% C.I.	
	B(SE)	***P=***	***OR***	***D_{Cohen}***	***Lower***	***Upper***
Warm-Glow	0.041 (0.016)	0.008	1.042	0.024	1.011	1.074
Gender	0.0003 (0.138)	0.998	1.000	0.000	0.764	1.310
Donor Age	0.020 (0.006)	0.001	1.020	0.011	1.008	1.032
Donor Status	0.133 (0.283)	0.639	1.142	0.073	0.656	1.987
Donation Type	-1.466 (0.206)	<0.001	0.231	-0.808	0.154	0.345
Donor Status x Donation Type	0.231 (0.322)	0.472	1.260	0.127	0.671	2.368
Booking Status	1.408 (0.147)	<0.001	4.088	0.776	3.068	5.448
Constant	-0.841 (0.346)	0.012	0.431	-0.464	0.223	0.832
R^2	.141					
n	1,124					

Supplementary Table 11: *Logistic Regression of Donor Status, Donation Type, Warm-Glow and booking to predict Donor Behaviour (male = 0, female = 1); Donor Status (first time = 0, novice = 1); Donation Type (Plasma = 0, Whole blood = 1); Booking Status (did not book an appointment = 0, booked an appointment= 1). Standard errors are standard. When robust standard errors are included to account for clustering both higher levels of warm-glow (B =0.041, Se = 0.02, P = 0.029, 95% CI = 0.004, 0.078) and having rebooked (B =1.4081, Se = 0.130, P <0.001 95% CI = 1.154, 1.662) remain significant predictors (Study 4). These analyses were conducted using logistic regression in SPSS 27. All analyses were two-tailed and no adjustments were made for multiple comparisons.*

Predicting Blood Donor Behaviour Study 5

Supplementary Table 12 shows the logistic GEE model predicting a return to donate within 6 months (73% returned to donate). The results show that both warm-glow and booking positively predicted return to donate.

Parameter	***B (se)***	***P =***	***OR***	***D_{Cohen}***	***95% C.I. for OR***	
					Lower	Upper
Constant	-1.464 (0.389)	<0. 001	0.232	-0.805	0.135	0.359
Gender	0.016(0.172)	0.892	1.017	0.009	0.800	1.291
Booking Status	0.803 (0.111)	<0.001	2.232	0.443	1.795	2.775
Warm-Glow	0.048 (0.013)	0.0003	1.049	0.026	1.022	1.076
Age	0.036 (0.004)	< 0.001	1.037	0.020	1.028	1.045
N (observations)	923 (1864)					

Supplementary Table S12. *Predicting Return Behaviour (Study 3). Booking Status (did not book an appointment = 0, booked = 1), Gender (male = 0, female = 1). B Coefficients are Unstandardized Coefficients.* (Study 5). Exact p-values for constant, $P = 2.647e^{-08}$, for booking status, $P = 4.971e^{-13}$, for age, $P = 2.382e^{-16}$. *These were implemented using the xtgee command in Stata 17. All analyses were two-tailed and no adjustments were made for multiple comparisons.*

Warm-Glow Influences Return Behaviour Via Commitment (Study 4)

To explore the hypothesis that one route through which warm-glow influences repeat high-cost cooperation is via facilitating booking, we ran a path model (Supplementary Figure 4) using data from Study 4. The model was specified in M*Plus* 8.4 using a diagonally weighted least squares estimator adjusted for means and variance to explore the potential indirect path from warm-glow to return behaviour via booking. While the relationship between warm-glow and booking is associational in these data and not directional, theoretically, we hypothesised that warm-glow drives booking. As such, we gave warm-glow causal precedence in the model. The results show direct effects for warm-glow and booking on return behaviour but also an indirect effect of warm-glow on return behaviour via booking ($B = 0.022$, Se = 0.006, $P<0.001$. 95%C.I. = 0.013, 0.031).

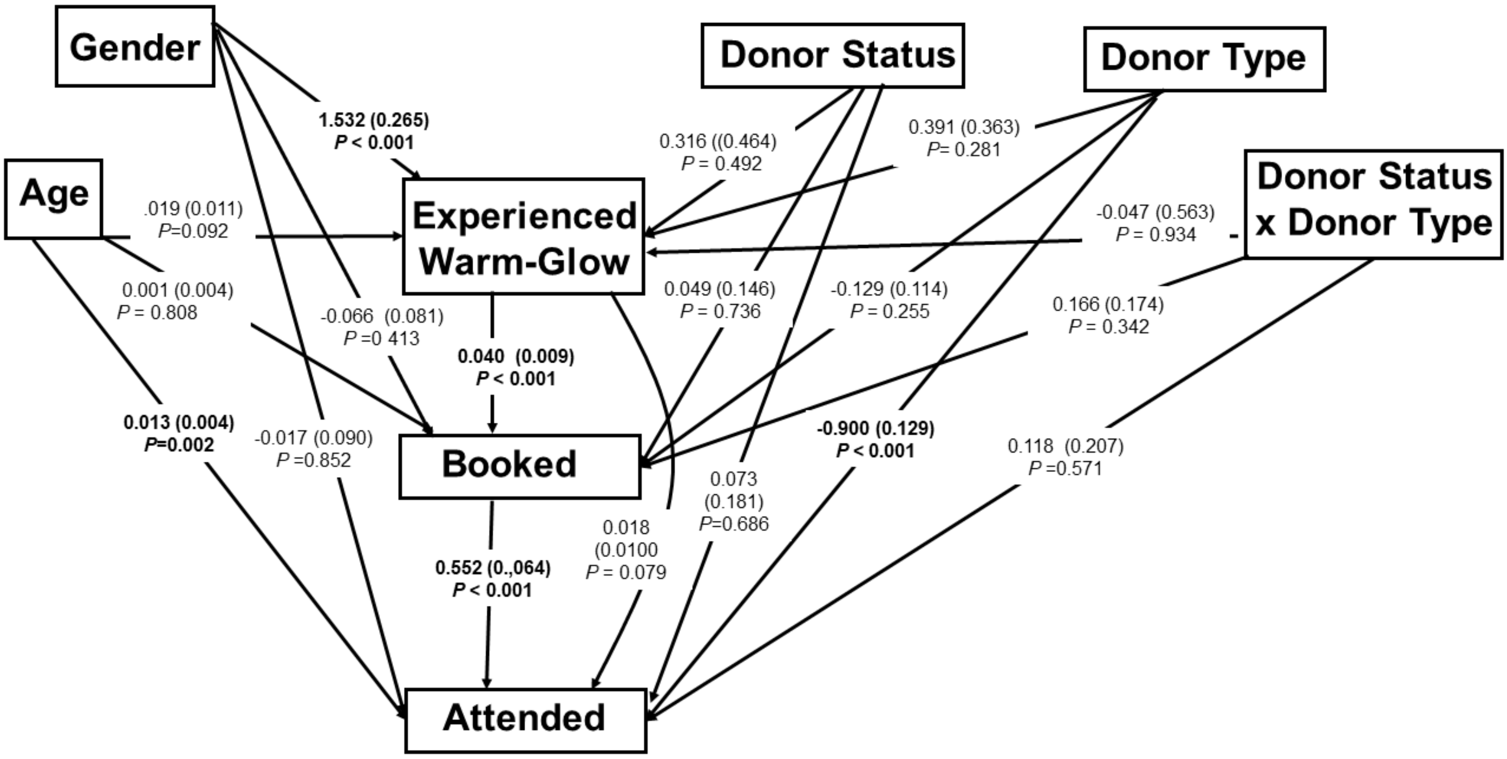

Supplementary Figure 4: *Path model of the process of book to attendance via warm-glow and the influence of age, sex, donor status and donor type. Coefficients are unstandardized B. Coefficients in parentheses are standard errors. There is a significant indirect effect of warm-glow via booking status on attendance (B = 0.022, Se = 0.006, P <,0.001 95%C.I. = 0.013, 0.031)). Gender (male = 0, female = 1); Donor Status (first time = 0, novice = 1); Donation Type (Plasma = 0, Whole blood = 1); Booking Status (did not book another appointment= 0, booked = 1). n =1124. All analyses were two-tailed and no adjustments were made for multiple comparisons*

Supplementary Files S6 for Study 6: Treatment Message Active Ingredients

This section provides additional supporting data for Study 6 on message evaluation.

Summary of Key Findings

The main findings are summarized here and detailed below. Warm-glow messages afforded a greater relative donor focus than the impure-altruism messages ($M_{\text{warm-glow}}$ = -29.299, 95% C.I. -31.277, -27. 372 vs $M_{\text{impure-altruism}}$ = -15.959, 95% C.I. -17.881, -14.038) with the "warm-glow-plus-identity" message having the strongest donor focus. The impure-altruism messages afforded a higher rating for donating blood to maintain the blood supply ($M_{\text{warm-glow}}$ = 3.917, 95% C.I. 3.772, 4.062 vs $M_{\text{impure-altruism}}$ = 4.988, 95% C.I. 4.846, 5.131). The warm-glow messages afforded slightly lower warm-glow than (M = 11.211, 95% C.I. 11.009 11.413) than the impure-altruism messages (M = 11.921, 95% C.I. 11.722, 12.120). However, it should be noted that for both the warm-glow and impure-altruism messages, the levels of warm-glow were very high and near the maximum of 14. The slightly higher level observed for the impure-altruism messages may reflect that warm-glow has three sources (the donor, recipient and maintaining the blood supply) and only one in the warm-glow messages (the donor). This prediction is supported with exposure to an identity prime resulted in higher levels of warm-glow (M_{identity} = 11.714, 95% C.I. 11.510 vs $M_{\text{no-identity}}$ = 11.418, 95% C.I. 11.220, 11.615), confirming that identity as a donor is a part of what determines warm-glow.

Box-Plots for the Six Experimental Conditions

Below are the Box-Plots for the six experimental conditions (BAU/control, Identity-only, Impure-Altruism-Only, Impure-Altruism-plus-Identity, Warm-Glow-Only, Warm-Glow-plus-Identity), for (1) afforded feelings of warm-glow (Supplementary Figure 5), (2) afforded focus on the donor, both the donor and recipient or recipient (Supplementary Figure 6), and (3) afforded importance to maintain the blood supply (Supplementary Figure 7).

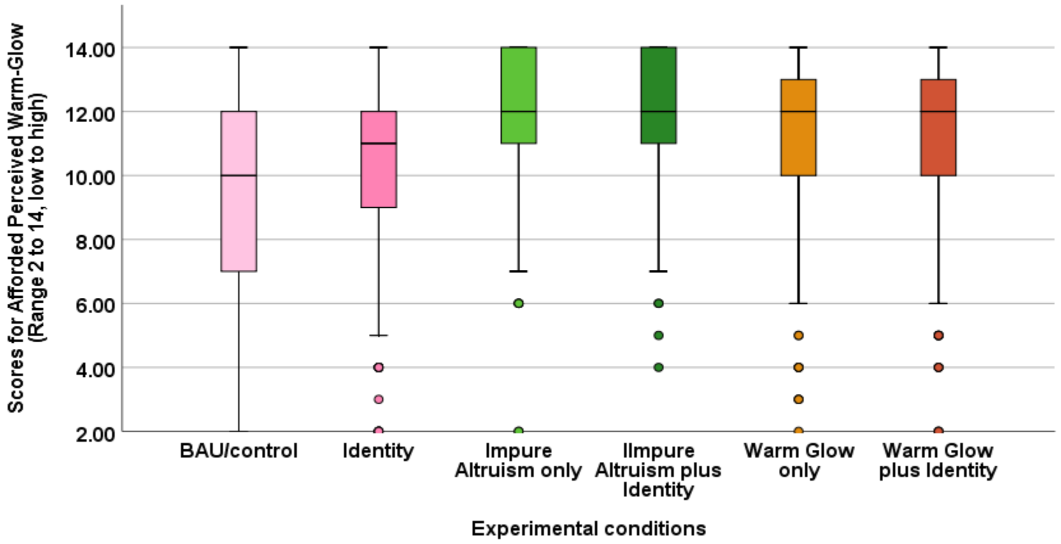

Supplementary Figure 5: *Box Plot for afforded feelings of warm-glow. (i) BAU (M = 9.5660; min = 2.0, max = 14.0; 95%CI = 9.1550, 9.9771; Sd = 3.39847; n = 265); (ii) Identity (M = 10.3296; min = 2.0, max = 14.0; 95%CI = 9.9621, 10.6972; Sd = 3.06780; n = 270), (iii) Warm-Glow-Only (M = 11.0798; min = 2.0 max = 14.0; 95%CI = 10.7622, 11.3975; Sd = 2.61625; n = 263), (iv) Warm-Glow-Plus-Identity (M = 11.3412, min = 2.0 max = 14.0 , 95%CI = 11.0175, 11.6648; Sd = 2.62432, n = 255), (v) Impure-Altruism-Only (M = 11.7554; min = 2.0 max = 14.0; 95%CI = 11.5047, 12.0061; Sd = 2.12376; n = 278), (vi) Impure-Altruism-Plus-Identity (M = 12.0863; min = 4.0 max = 14.0; 95%CI = 11.8473, 12.3253; Sd = 1.93812; n = 255). Central line of the box is the median, and the box indicates the middle of the distribution bounded by the 1st and 3rd quartiles. The whiskers indicate the minimum and maximum in these data. Where there are suspected outliers, these whiskers are defined as 1,5*IQR (inter-quartile range) and the circles indicate suspected outliers (removal of outliers made no difference to results reported in this paper and as such all analyses were based on the full dataset).*

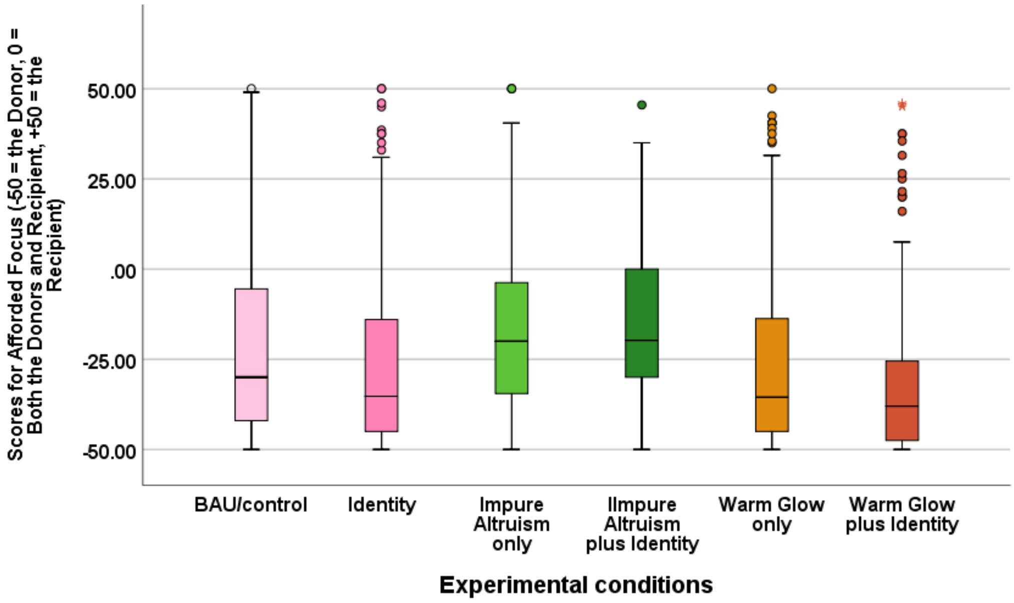

Supplementary Figure 6: *Box Plot for afforded focus on the donor, both the donor and recipient or recipient. (i) BAU (M = -22.9291, min = -50.0 max = 50.0; 95%CI = -25.9997, -19.8586; Sd = 24.84885; n = 254); (ii) Identity (M = -26.5150; min = -50.0 max = 50.0, 95%CI = -29.4251, -23.6050; Sd = 24.10468; n = 266), (iii) Warm-Glow-Only (M = -27.1451; min = -50.0 max = 50.0; 95%CI = -30.0730, -24.2172; Sd = 23.74103; n = 255), (iv) Warm-Glow-Plus-Identity (M = -31.4538, min = -50.0 max = 46.0; 95%CI = -34.1054, -28.8022; Sd = 21.24376; n = 249), (v) Impure-Altruism-Only (M = -16.8674; min = -50.0 max = 50.0; 95%CI = -19.5044, -14.2304; Sd = 2.12376; n = 264), (vi) Impure-Altruism-Plus-Identity (M = -15.0512; min = -50.0 max = 45.0; 95%CI = -17.7414, -12.3610; Sd = 21.33368; n = 244). Central line of the box is the median, and the box indicates the middle of the distribution bounded by the 1st and 3rd quartiles. The whiskers indicate the minimum and maximum in these data. Where there are suspected outliers, these whiskers are defined as 1,5*IQR (inter-quartile range) and the circles indicate suspected outliers and a * (star) any potential extreme outlier (removal of outliers made no difference to results reported in this paper and as such all analyses were based on the full dataset).*

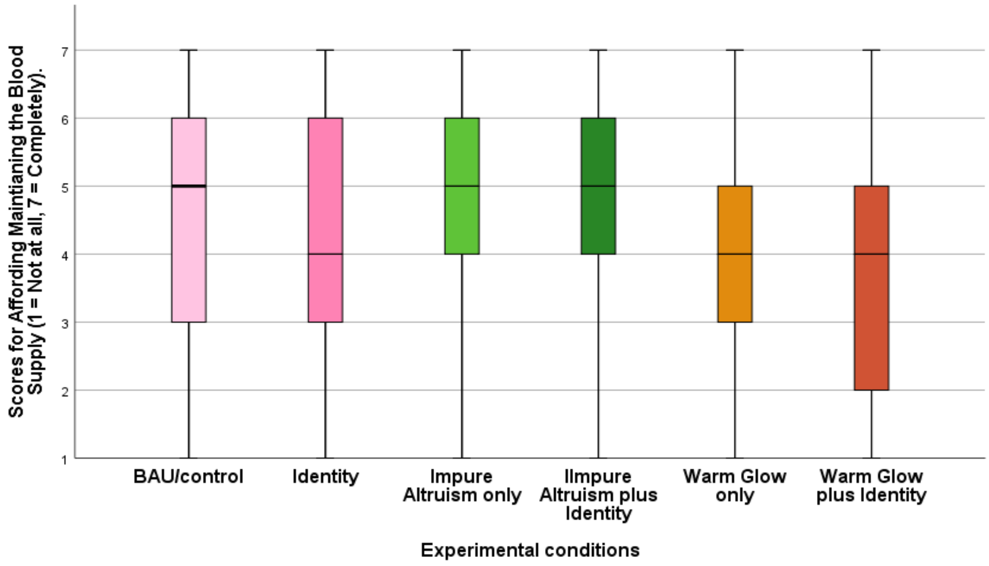

Supplementary Figure 7: *Box Plot for afforded importance to maintain the blood supply. (i) BAU (M = 4.38; min = 1 max = 7; 95%CI = 3.4.16, 4.59; Sd = 1.760; n = 266); (ii) Identity (M = 4.16; min = 1 max = 7; 95%CI = 3.96, 4.37; Sd = 1.691; n = 273), (iii) Warm-Glow-Only (M = 3.90; min = 1 max = 7; 95%CI = 3.69, 4.12; Sd = 1.768; n = 263), (iv) Warm-Glow-Plus-Identity (M = 3.93; min = 1 max = 7; 95%CI = 3.69, 4.17; Sd = 1.924; n = 255), (v) Impure-Altruism-Only (M = 4.96; min = 1 max = 7; 95%CI = 4.78, -5.14; Sd = 1.519; n = 279), (vi) Impure-Altruism-Plus-Identity (M = 5.02; min = 1 max =7; 95%CI = 4.84, 5.20; Sd = 1.478; n = 255). Central line of the box is the median, and the box indicates the middle of the distribution bounded by the 1st and 3rd quartiles.*

Warm-Glow By Identity Interactions

Afforded Levels of Perceived Warm-Glow

A 2 (warm-glow: warm-glow vs impure altruism) by 2 (identity: present vs absent) between-subjects ANOVA on the sum of the two items (possible range 2 to 14) was used to assess feelings of warm-glow. The analyses showed a significant main effect for warm-glow ($F_{(1, 1047)} = 24.126$, $P < 0.001$, $\eta_p^2 = .023$), such that the warm-glow messages afforded slightly lower warm-glow than the impure altruism message ($M_{\text{warm-glow}} = 11.211$, 95% C.I. 11.009 11.413 vs $M_{\text{impure-altruism}} = 11.921$ 95% C.I. 11.722, 12.120). However, it should be noted that for both the warm-glow and impure-altruism messages, the levels of perceived warm-glow are very high and near the maximum of 14. There was a significant effect for identity ($F_{(1, 1047)} = 4.192$, $P = 0.041$, $\eta_p^2 = .004$: $M_{\text{identity}} = 11.714$ 95% C.I. 11.510, 11.917 vs $M_{\text{no-identity}} = 11.418$, 95% C.I. 11.220, 11.615). This confirms our prediction that identity as a donor is a part of what determines warm-glow. The was no significant interaction between warm-glow and identity ($F_{(1, 1047)} = 0.058$, $P = 0.810$, $\eta_p^2 = .000$). The means for all cells can be found in Supplementary Table 13.

Warm-Glow manipulation	Identity manipulation	Mean	95% Confidence Interval	
			Lower Bound	Upper Bound
Impure Altruism	Absent	11.755	11.480	12.031
	Present	12.086	11.798	12.374
Warm-Glow	Absent	11.080	10.796	11.363
	Present	11.341	11.053	11.629

Supplementary Table 13: *Means for warm-glow (feeling good plus rewarding) as a function of the warm-glow and identity manipulations.*

Affording Maintain the Blood Supply

A 2 (warm-glow: warm-glow vs impure altruism) by 2 (identity: present vs absent) between-subjects ANOVA on a question asking participants indicate the extent (1 = not at all, to 7 = completely) that the "Message makes me feel that that donating blood would ensure that there is enough blood for all who need it." There was a significant main effect for warm-glow ($F_{(1, 1048)}$ = 106.842, P < 0.001, η_p^2 = .093), with warm-glow messages affording a lower importance than the impure altruism message that donating blood was important to maintain the blood supply ($M_{\text{warm-glow}}$ = 3.917, 95% C.I. 3.772, 4.062 vs $M_{\text{impure-altruism}}$ = 4.988, 95% C.I. 4.846, 5.131). There was no significant effect for identity ($F_{(1, 1048)}$ = 0.177, P = 0.674, η_p^2 = .000: M_{identity} = 4.475, 95% C.I. 4.329, 4.620 vs $M_{\text{no-identity}}$ = 4.431, 95% C.I. 4.289, 4.573). The was no significant interaction between warm-glow and identity ($F_{(1, 1048)}$ = 0.034, P = 0.852, η_p^2 = .000). The means for all cells are in Supplementary Table 14.

Warm-Glow manipulation	Identity manipulation	Mean	95% Confidence Interval	
			Lower Bound	Upper Bound
Impure Altruism	Absent	4.957	4.760	5.154
	Present	5.020	4.813	5.226
Warm-Glow	Absent	3.905	3.702	4.108
	Present	3.929	3.723	4.136

Supplementary Table 14: *Interaction of Warm-Glow and Identity on Perceptions of Maintaining the Blood Supply. This was assessed with a single item (1 = not at all, to 7 = completely: "The message makes me feel that that donating blood would ensure that there is enough blood for all who need it.").*

The Afforded Focus of Donation: The Donor, the Recipient or Both.

A 2 (warm-glow: warm-glow vs impure altruism) by 2 (identity: present vs absent) between-subjects ANOVA on the focus of donation (primarily on the blood donor [-50], equally focuses on both the donor and recipient [0], focuses primarily on the recipient of blood [+50]), showed a significant main effect for warm-glow ($F_{(1, 1008)}$ = 92.500, P < 0.001 η_p^2 = .084), such that the warm-glow messages afforded a greater relative donor focus than the impure-altruism messages ($M_{\text{warm-glow}}$ = -29.299, 95% C.I. -31.277, -27. 372 vs $M_{\text{impure-altruism}}$ = -15.959, 95% C.I. -17.881, -14.038). There was no significant effect for identity ($F_{(1, 1008)}$ = 0.807, P = 0.369, η_p^2 = .001: M_{identity} = -23.253, 95% C.I. -25.202, -21.303 vs $M_{\text{no-identity}}$ = -22.006, 95% C.I. -23.906, -20.106). The was a significant interaction between warm-glow and identity ($F_{(1, 1008)}$ = 4.875, P = 0.027, η_p^2 = .005) which is shown in Supplementary Figure 8 as a clustered box-plot with the means and 95% C.I.s are reported in Supplementary

Table 15. This shows that the warm-glow-plus-identity message had the strongest donor focus.

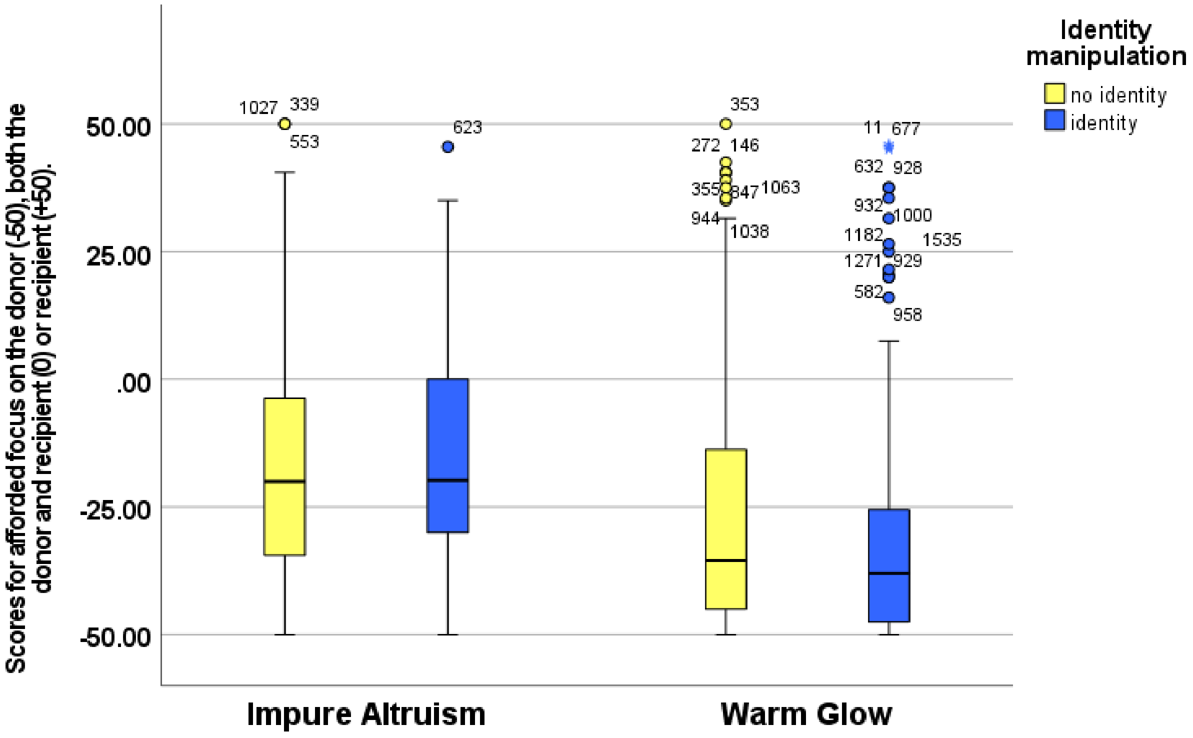

Supplementary Figure 8. *Cluster Box Plot for the Interaction of Warm-Glow by Identity Messages on Donor and Recipient Focus. Central line of the box is the median, and the box indicates the middle of the distribution bounded by the 1st and 3rd quartiles. The whiskers indicate the minimum and maximum in these data. Where there are suspected outliers these whiskers are defined as 1,5*IQR (inter-quartile range) and the circles indicate suspected outliers and a * (star) any potential extreme outlier. The numbers around a circle or star indicate for that score there are more than one participant with that scores. In terms of means the following values were observed. For exposure to an impure altruism message with no identity prime the mean was -16.867 (95% CI = -19.531, -14.204: n = 264) and exposure to an impure altruism message with an identity prime the mean was -15.051 (95% CI = -17.882, -12.281: n = 244). For exposure to a warm-glow message with no identity prime the mean was -27.145 (95% CI = -29.855, -24.435: n = 255) and exposure to a warm-glow message with an identity prime the mean was -31.454 (95% CI =-34.196, -28.711: n = 249).*

**Warm-Glow manipulation**	**Identity manipulation**	**Mean**	**95% Confidence Interval**	
			Lower Bound	Upper Bound
Impure Altruism	Absent	-16.867	-19.531	-14.204
	Present	-15.051	-17.822	-12.281
Warm-Glow	Absent	-27.145	-29.855	-24.435
	Present	-31.454	-34.196	-28.711

Supplementary Table 15: *Warm-Glow and Impure Altruism Message on Focus. The average of two items to assess whether the focus afforded is primarily on the blood donor (-50) equally focuses on both the donor and recipient (0), focuses primarily on the recipient of blood (+50).*

Moderation by Donor Status

Below are the ANOVA tables that include donor status in terms of whether or not the participants had ever donated blood to give in a 2 (warm-glow: warm-glow; impure-altruism) by 2 (identity: present; absent) by 2 (donor status: ever donated; never donated) between-subjects ANOVA. Supplementary Table 16 contains the results for the focus of a donation being on the donor, the recipient or both, Supplementary Table 17 for the maintenance of the blood supply and Supplementary Table 18 for perceived warm-glow. In all cases, the results replicate those in the main text with no significant effects for blood donor status.

Source	***Type III Sum of Squares***	***df***	***Mean Square***	***F***	***P =***	η_p^2
Corrected Model	49499.929	7	7071.418	14.633	< 0.001	.093
Intercept	469379.482	1	469379.482	971.311	< 0.001	.494
Warm-Glow	35704.859	1	35704.859	73.886	< 0.001	.069
Identity	21.855	1	21.855	.045	0.832	.000
Ever Donated Blood	78.992	1	78.992	.163	0.686	.000
Warm-Glow * Identity	1853.870	1	1853.870	3.836	0.050	.004
Warm-Glow * Ever Donated Blood	1252.463	1	1252.463	2.592	0.108	.003
Identity * Ever Donated Blood	755.156	1	755.156	1.563	0.212	.002
Warm-Glow * Ever Donated Blood * Identity *	87.054	1	87.054	.180	0.671	.000
Error	481310.443	996	483.243			
Total	1049556.250	1004				
Corrected Total	350810/371	1003				

Supplementary Table 16. *Focus on donor, recipient or both as a function of warm-glow (warm-glow; impure-altruism), identity (present; absent) and donor status (ever donated; never donated). These were conducted using the univariate ANOVA command in SPSS 27. All analyses were two-tailed, and no adjustments were made for multiple comparisons.*

Source	*Type III Sum of Squares*	*df*	*Mean Square*	*F*	*P =*	η_p^2
Corrected Model	306.290	7	43.756	15.535	< 0.001	.095
Intercept	18700.501	1	18700.501	6639.545	< 0.001	.865
Warm-Glow	287.180	1	287.180	101.962	< 0.001	.090
Identity	.944	1	.944	.335	0.563	.000
Ever Donated Blood	.107	1	.107	.038	0.846	.000
Warm-Glow * Identity	.087	1	.087	.031	0.861	.000
Warm-Glow * Ever Donated Blood	4.252	1	4.252	1.510	0.219	.001
Identity * Ever Donated Blood	3.118	1	3.118	1.107	0.293	.001
Warm-Glow * Ever Donated Blood * Identity *	.733	1	.733	.260	0.610	.000
Error	2915.112	1035	2.817			
Total	24006.000	1043				
Corrected Total	3221.402	1042				

Supplementary Table 17. *Maintaining the blood supply as a function of warm-glow (warm-glow; impure-altruism), identity (present; absent) and donor status (ever donated; never donated). These were conducted using the univariate ANOVA command in SPSS 27. All analyses were two-tailed and no adjustments were made for multiple comparisons.*

Source	*Type III Sum of Squares*	*df*	*Mean Square*	*F*	*p =*	η_p^2
Corrected Model	161.209	7	23.030	4.282	< 0.001	.028
Intercept	126366.270	1	126366.270	23497.828	< 0.001	.958
Warm-Glow	130.454	1	130.454	24.258	< 0.001	.023
Identity	18.952	1	18.952	3.524	0.061	.003
Ever Donated Blood	.329	1	.329	.061	0.805	.000
Warm-Glow * Identity	.629	1	.629	.117	0.732	.000
Warm-Glow * Ever Donated Blood	1.882	1	1.882	.350	0.554	.000
Identity * Ever Donated Blood	1.127	1	1.127	.210	0.647	.000
Warm-Glow * Ever Donated Blood * Identity *	4.904	1	4.904	.912	0.340	.001
Error	5560.630	1034	5.378			
Total	145720.000	1042				
Corrected Total	5721.839	1041				

Supplementary Table 18. *Perceived warm-glow as a function of warm-glow (warm-glow; impure-altruism), identity (present; absent) and donor status (ever donated; never donated). These were conducted using the univariate ANOVA command in SPSS 27. All analyses were two-tailed and no adjustments were made for multiple comparisons.*

Planned Contrasts

Below are reported a series of planned comparisons comparing the warm-glow and impure-altruism messages (with and without identity primes) to the pure control (a simple reminder message) and an identity-only message. The means and standard deviations are presented in Supplementary Table 19.

Donor vs Patient Focus: A one-way between-subjects ANOVA at 6 levels ($F_{(1, 1526)} = 19.307$, $P < 0.001$, $\eta_p^2 = .059$) showed a significant difference across the 6 messages. A planned pairwise comparison of the warm-glow message to the impure-altruism message was significant ($t_{(1526)} = 9.36$, $P < 0.001$,: Cohen's d = 0.479) showing that the warm-glow message (*M* = -29.299; *95% C.I.* = -31.227, -27.372) was perceived as more *donor-focused* than the impure-altruism message (*M* = -15.959 *95% C.I.* = -17.881, -14.038), which was perceived to focus on both donor and recipient. A second planned pairwise comparison of the warm-glow message with the combined impure-altruism, BAU, and identity message conditions was significant ($t_{(1526)} = 7.192$, $P < 0.001$,: Cohen's d = 0.471), indicating that the warm-glow message was perceived to be more donor-focused than the combined conditions.

Perceived Warm-Glow: A one-way between-subjects ANOVA on perceived warm-glow ($F_{(5, 1580)} = 32.26$, $P < 0.001$, $\eta_p^2 = .093$) was significant. A planned pairwise comparison of the warm-glow message and impure-altruism messages to the BAU and identity-only messages was significant ($t_{(1580)} = 11.374$, $P < 0.001$,: Cohen's d = 0.572) indicating that the warm-glow and impure-altruism messages (*M* = 11.566; *95% C.I.* = 11.403, 11.730) were perceived as engendering higher levels of experienced warm-glow than the BAU and identity-only conditions (*M* = 9.951; *95% C.I.* = 9.722, 10.180). A second planned contrast comparing the warm-glow messages to the impure-altruism messages was also significant ($t_{(1580)} = 4.298$, $P < 0.001$,: Cohen's d = 0.216), indicating that the warm-glow messages (*M* = 11.211; *95% C.I.* = 11.009, 11.413) were perceived as having engendered lower warm-glow than the impure-altruism messages (*M* = 11.921; *95% C.I.* = 11.722, 12.120).

Focus on Maintaining the Blood Supply: A one-way between-subjects ANOVA ($F_{(5, 1585)} = 22.318$, $P < 0.001$, $\eta_p^2 = .066$) revealed a significant difference across the 6 conditions. A planned comparison of the warm-glow message with the impure-altruism message showed a significantly higher perceived focus on maintaining the blood supply for the impure-altruism message ($t_{(1585)} = 10.241$, $P < 0.001$,: Cohen's d = 0.514); *M* = 4.988; *95% C.I.* = 4.846, 5.131) than the warm-glow message (*M* = 3.917; *95% C.I.* = 3.772, 4.062). A second planned comparison of the warm-glow messages to the combined BAU and identity-only condition ($t_{(1585)} = 3.386$, $P = 0.001$: Cohen's d = 0.170) showed that the warm-glow messages (*M* = 3.917; *95% C.I.* = 3.763, 4.071) had a significantly lower focus on maintaining the blood supply than the BAU and identity-only message (*M* = 4.269; *95% C.I.* = 4.118, 4.420).

	Message Validation Focus					
	Donor-Recipient Focus		Perceived Warm-Glow		Blood Supply	
Condition	Mean (n)	SD	Mean (n)	SD	Mean (n)	SD
BAU/control	-22.929 (254)	24.848	9.566 (265)	3.39847	4.38 (266)	4.38
Identity only	-26.515 (266)	24.104	10.329 (270)	3.06780	4.16 (273)	4.16
Warm-Glow only	-27.145 (255)	23.741	11.079 (263)	2.61625	3.90 (263)	3.90
Warm-Glow plus Identity	-31.454 (249)	21.244	11.341 (255)	2.62432	3.93 (255)	3.93
Impure Altruism only	-16.867 (264)	21.760	11.755 (278)	2.12376	4.96 (279)	4.38
Impure Altruism plus Identity	-15.051 (244)	21.334	12.086 (255)	1.93812	5.02 (266)	4.16

Supplementary Table 19: *Warm-Glow and Impure Altruism Message Validation.* ***Donor-Recipient Focus:*** *The average of two items to assess whether the focus is primarily on the blood donor (-50), equally focuses on both the donor and recipient (0), and focuses primarily on the recipient of blood (+50).* ***Perceived Warm-Glow****: The sum of two items (possible range 2 to 14) was used to assess feelings of warm-glow.* ***Blood Supply****: This was assessed with a single item (1 = not at all, to 7 = completely: "The message makes me feel that donating blood would ensure that there is enough blood for all who need it.").*

Supplementary Files S7: Sampling Strategies and Sample Bias

This section provides the details of the sampling strategies used and documents any sample biases.

Sample Bias and Randomization Checks for Study 1: Field-Based Experiment

Field-Based Experiment Treatment Allocations: First-time donors were randomized to one of the five experimental treatments, the Business as Usual (BAU) control, and four active message treatments (warm-glow-only warm-glow-plus-identity, impure-altruism-only, and impure-altruism-plus-identity). This is shown in Supplementary Figure 9 below.

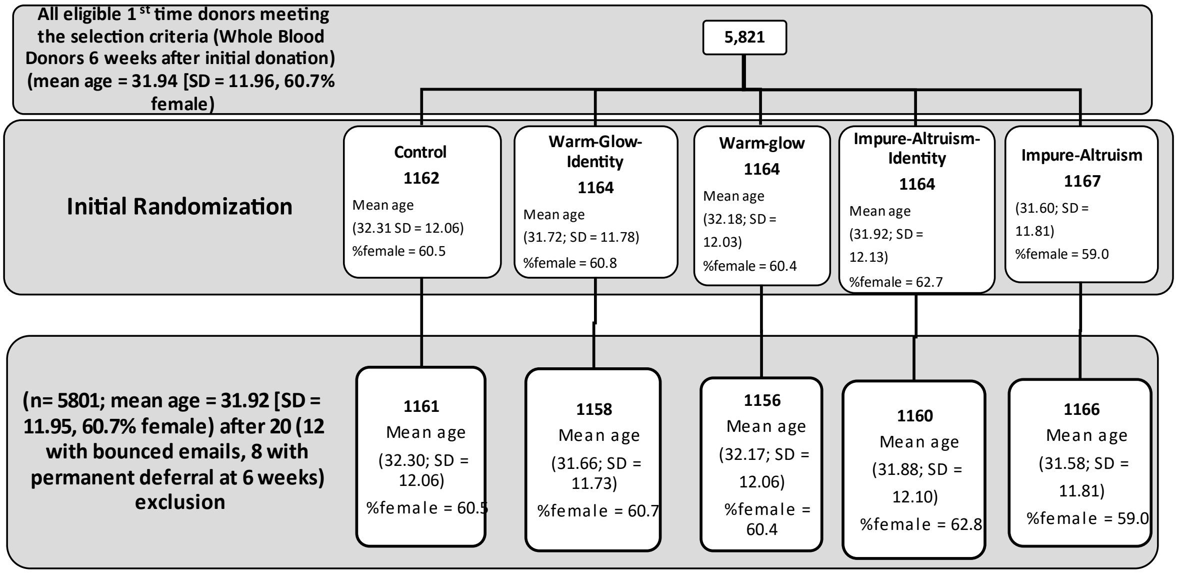

Supplementary Figure 9. *Randomization to Treatments, detailing n, age and gender*

Randomization Checks. With respect to the initial randomization of 5,821 donors, age did not vary significantly across treatments ($F_{(4, 5816)} = 0.725$, $P = 0.575$: $D_{Cohen} = 0.059$), nor did sex (χ^2 (4) = 3.542, $P = 0.472$: $D_{Cohen} = 0.050$). The majority of the sample had blood type O+ (45.9%), followed by A+ (36.9%), and O- (10.7%), A- (6.4%) and O (1%). The distribution of blood type did not vary by treatment (χ^2 (16) = 18.16, $P = 0.314$: D_{Cohen} = 0.112). The percentage who booked immediately post-donation was 37.7%, with 62.3% not booking while in the donor centre, and this did not vary by condition (χ^2 (4) = 4.163, P = 0.384: $D_{Cohen} = 0.054$).

After the 20 donors were excluded (Supplementary Figure 8), donor age remained nonsignificant by condition ($F_{(4, 796)} = 0.796$, $P = 0.527$: $D_{Cohen} = 0.059$), as did sex (χ^2 (4) = 3.536, $p = 0.472$: $D_{Cohen} = 0.054$). The majority of the sample had blood group O+ (46%), followed by A+ (36.9%), and O- (10.7%), A- (6.4%). The distribution of blood type did not

vary by condition (χ^2 (12) = 14.62, P = 0.263; D_{Cohen} = 0.100). The percentage who booked was 37.7% with 62.3% not initially booking and this did not vary by condition (χ^2 (4) = 4.198, P = 0.380: D_{Cohen} = 0.054). Thus, the randomization by arm remained intact after the exclusions.

Sampling Strategy and Sample Bias Study 3: Booking Another Appointment, Warm-Glow and Pure Altruism

Sampling and sample: Soft quotas for participant demographic and donation characteristics were applied to gender, type of blood, donation frequency, first-time or returning donor as applicable. First-time donors were over-sampled. The a-priori aim was to collect responses from 2,000-3,000. With an anticipated response rate of 10-15%, based on previous similar studies, 20,000 donors were contacted with an even split across the 3 groups; (1) first-time donors (first donated after February 2020), (2) existing eligible donors who had donated after February 2020, and (3) existing eligible donors who had not donated since February 2020. Sampling was targeted to ensure that the gender balance and age (18-69: donors over 70 were excluded) distribution were representative of the donor population.

Nine-hundred and sixty-six first-time donors were recruited (mean age = 39.37 years, *Sd* = 15.03 years; 66% female with 23.7% having had a COVID-19 test). The low number of COVID-19 tests reflects the low rate of COVID-19 infection in Australia at the time. Of these 250 first-time donors started the survey but did not complete the majority of measures including the measure of adjectives used to assess warm-glow. As 250 did not complete the survey (non-completers) but started it, and 716 did (completers), there is a potential for selection bias. Indeed, completer status is predicted by having had a COVID-19 test (χ^2 (1) = 3.373, P = 0.066, φ = .061) with those who had a COVID-19 test more likely to be completers, and being older (M = 41.20, Sd = 14.85 vs M = 34.14, Sd = 14.33; $t_{(964)}$ = 6.531, P < 0.001; D_{Cohen} = 0.421). Completer status was not predicted by gender (χ^2 (1) = 0.020, P = 0.887, φ = -.005). Booking status was not predicted by being a non-completer or completer (χ^2 (1) = 1.151, P = 0.283, φ = .035) nor as a function of having had a COVID-19 test (χ^2 (1) = 3.315, P = 0.069, φ = .061). To account for any potential selection bias we completed a sensitivity analysis using a Heckman Probit Selection model with COVID-19 test, gender and age as predictors of selection bias.

Sampling Strategy and Sample Bias Study 4: Warm-Glow, Booking Another Appointment and Donor Status and Type

Sampling and sample: New (1st donation) and novice (1 or 2 previous donations and no plasma donations for whole blood donors, and 1 or 2 previous plasma donations and fewer than 3 whole blood donations for plasma donors) donors who were either whole blood or plasma donors were recruited by convenience sampling at three large urban Australian Red Cross Lifeblood (Lifeblood) donation centres (Brisbane Donor Centre, Sydney Town Hall and Melbourne Mt Waverley) from April 2016 to October 2017.

A total sample of 1,153 donors were recruited. Of these, 441 were first-time whole blood donors (M age = 30.47, Sd = 11.86, n = 413; % female = 55.6%, n = 412), 218 first-time plasma donors (M age = 30.95, Sd = 11.30, n = 218; % female 50.5%, n = 218), 385 were novice whole blood donors (M age = 29.52, Sd 11.47, n = 385; % female = 60.8%, n = 385) and 136 novice plasma donors (M age = 30.5, Sd = 10.75 n = 136; % female = 49.3%, n = 136;). Age did not vary significantly across these groups ($F_{(3, 1148)}$, = 0.860, P = 0.461, η_p^2

= .002) but gender did (χ^2 (3) = 8.73, *P* = 0.033, φ = .087). Overall, there were 354 plasma donors (*M* age = 30.79, *Sd* = 11.19, *n* = 345, % female = 50.0%, *n* = 354) and 799 whole blood donors (*M* age = 30.01, *Sd* = 11.64, *n* = 798; % female = 57.4%, *n* = 797) who did not differ by age ($t_{(1150)}$ = 1.06, *P* = 0.289; D_{Cohen} = 0.063) but did by gender (χ^2 (1) = 6.50, *P* = 0.011, φ = -.075). There were 632 first-time donors (*M* age = 30.64, *Sd* = 11.67, *n* = 631; % female = 53.8%, *n* = 630) and 521 novice donors (*M* age = 29.79, *Sd* = 11.26, *n* = 521; % female = 57.5%, *n* = 521) who did not differ by age ($t_{(1150)}$ = 1.25, *P* = 0.212; D_{Cohen} = 0.074) or gender (χ^2 (1) = 1.81, *P* = 0.178, φ = -.040).

There were missing data on warm-glow (n = 26), age (n = 1) and gender (n = 2). Once these were removed the final sample size was 1,124. Of these, 401 were first-time whole blood donors (*M* age = 30.38, *Sd* = 11.879; % female = 54.6%, *n* = 219 women), 208 first-time plasma donors (*M* age = 30.07, *Sd* = 11.50, % female 50.5%, *n* = 106 women), 379 were novice whole blood donors (*M* age = 29.61, *Sd* 11.49, % female = 60.4%, *n* = 229 women) and 136 novice plasma donors (*M* age = 30.53, *Sd* = 10.75; % female = 49.3%, *n* = 67 women). Neither age ($F_{(3, 1120)}$, = 0.787, *P* = 0.501, η_p^2 = .002) nor gender = (χ^2 (3) = 7.68, *P* = 0.053, φ = .083) significant varied across these groups. Overall, there were 344 plasma donors (*M* age = 30.85, *Sd* = 11.19, % female = 50.3%, *n* = 173 women) and 780 whole blood donors (*M* age = 30.01, *Sd* = 11.65, % female = 57.4%, *n* = 797) who did not differ by age ($t_{(1122)}$ = 1.14, *P* = 0.254; D_{Cohen} = 0.068) but did by gender (χ^2 (1) = 4.93, *P* = 0.026, φ = -.066). There were 609 first-time donors (*M* age = 30.62, *Sd* = 11.69; % female = 53.4%, *n* = 325 women) and 515 novice donors (*M* age = 29.85, *Sd* = 11.30; % female = 57.5%, *n* = 296 women) who did not differ by age ($t_{(1122)}$ = 1.11, *P* = 0.267; D_{Cohen} = 0.066) or gender (χ^2 (1) = 1.81, *P* = 0.178, φ = -.040).

Sampling Strategy and Sample Bias Study 5: Temporal Stability of Warm-Glow Following Booking Another Appointment

Sampling and Sample. All 25,428 first-time whole blood donors were invited to participate in the study between January 2017 and September 2017. 4,472 first-time donors were recruited (*M* age = 35.16, *Sd* = 13.55; % female = 68%). Of these, data were missing from 500 participants on intrinsic regulation (warm-glow) at wave 1, leaving complete data on 3,972 participants. We did not impute these missing data as data were missing on all items pertaining to warm-glow and the missing percentage was high (11%). Of the 4,472 recruited donors, 136 donors were actively deferred from donating for medical and life-style reasons. Those deferred were more likely to be women (χ^2 (1) = 18.296, *P* < 0.001, φ = .064) but did not vary by age (M_{deferred} = 33.88, *Sd* = 12.83 vs $M_{\text{not deferred}}$ = 35.20, *Sd* = 13.57: $t_{(4470)}$ = 1.119, *P* = 0.263; D_{Cohen} = 0.033). Those deferred were not significantly different from those not-deferred on warm-glow at wave 1 (M_{deferred} = 15.06, *Sd* = 4.34 vs $M_{\text{not deferred}}$ = 15.18, *Sd* = 4.09: $t_{(3970)}$ = 0.315, *P* = 0.753; D_{Cohen} = 0.009) or wave 4 (M_{deferred} = 15.48, *Sd* = 4.09 vs $M_{\text{not deferred}}$ = 15.21, *Sd* = 4.07: $t_{(955)}$ = -0.329, *P* = 0.743; D_{Cohen} = -0.021). However, as deferral status influenced the decision to book (χ^2 (1) = 5.571, *P* = 0.016, φ = -.036), with those deferred less likely to book, we excluded those who were actively deferred from the analyses.

After removal of those actively deferred there were 4,336 first-time donors (*M* age = 35.20 years, *Sd* = 15.57 years; % female = 67%) at wave 1. At wave 4 a total of 932 completed the survey providing data on warm-glow. Those who completed the surveys providing data on warm-glow at waves 1 and 4 were significantly older ($M_{\text{Wave 1 only}}$ = 34.88, *Sd* = 13.39 vs $M_{\text{Waves 1 \& 2}}$ = 36.27, *Sd* = 14.09: $t_{(4334)}$ = -2.863 *P* = 0.004; D_{Cohen} = -0.087) and less likely to be male (χ^2 (1) = 8.231, *P* = 0.004, φ = -.044) than those who completed only wave 1, however, they did not significantly vary on warm-glow at wave 1 ($M_{\text{Time 1 only}}$ =

15.25, *Sd* = 4.06 vs $M_{\text{time1 \& 2}}$ = 14.97, *Sd* = 4.18: $t_{(3849)}$ = 1.892, *P* = 0.059; D_{Cohen} = 0.061). Furthermore, warm-glow at wave 4, for those who completed both surveys (*M* = 15.21, *Sd* = 4.06) was not significantly different from warm-glow at wave 1 (*M* = 15.25, *Sd* = 4.06) in those who just completed the survey at time 1 ($t_{\text{(one-sample (931)}}$ = -0.320, *P* = 0.749; D_{Cohen} = -0.021). As such, there is no evidence that initial levels of warm-glow influenced who remained in the study from waves 1 to waves 4. However, we controlled for age and gender in these analyses.

Sampling Strategy and Sample Bias for Study 6: Message Validation

Sampling Strategy. Participants (ages 18 and 80 from the UK) were recruited through Prolific (https://www.prolific.co/) to an experiment hosted on Qualtrics (https://www.qualtrics.com/uk/). All data were collected on the 24th of March 2021. Participants were block-randomized by sex to each of the 6 conditions. A total of 1,592 were recruited (M_{age} = 36.47, *Sd* = 13.00, % female = 52%). 34% (*n* = 538) indicated that they had ever donated blood and 12.1% (*n* = 194) were current donors (donated within the last 2 years). There were 266 participants in the BAU/Control, 273 in the ‘identity-only’, 263 in the ‘warm-glow-only’, 256 in the ‘warm-glow-plus-identity’, 279 in the ‘impure-altruism-only’, and 255 in the ‘impure-altruism-plus-identity’ conditions. Neither age ($F_{(5, 1583)}$ = 1.770, *P* = 0.116, η_p^2 = .006), gender (χ^2 (5) = 0.863, *P* = 0.973, φ = .023), ever donated blood (χ^2 (5) = 8.918, *P* = 0.112, φ = .075) nor being a current blood donor (χ^2 (5) = 1,221,*P* = 0.943, φ = .028) significantly varied by condition.

Supplementary Files S8: Timeline for Field-Based Experiment (Study 1) and the Implementation Analysis (Study 2)

Timeline: Supplementary Figure 10 details the dates of the field-based experiment, the implementation of the best warm-glow message, and the dates for the pre-post implementation analysis. The methodology of the field-based experiment and implementation trial is detailed below.

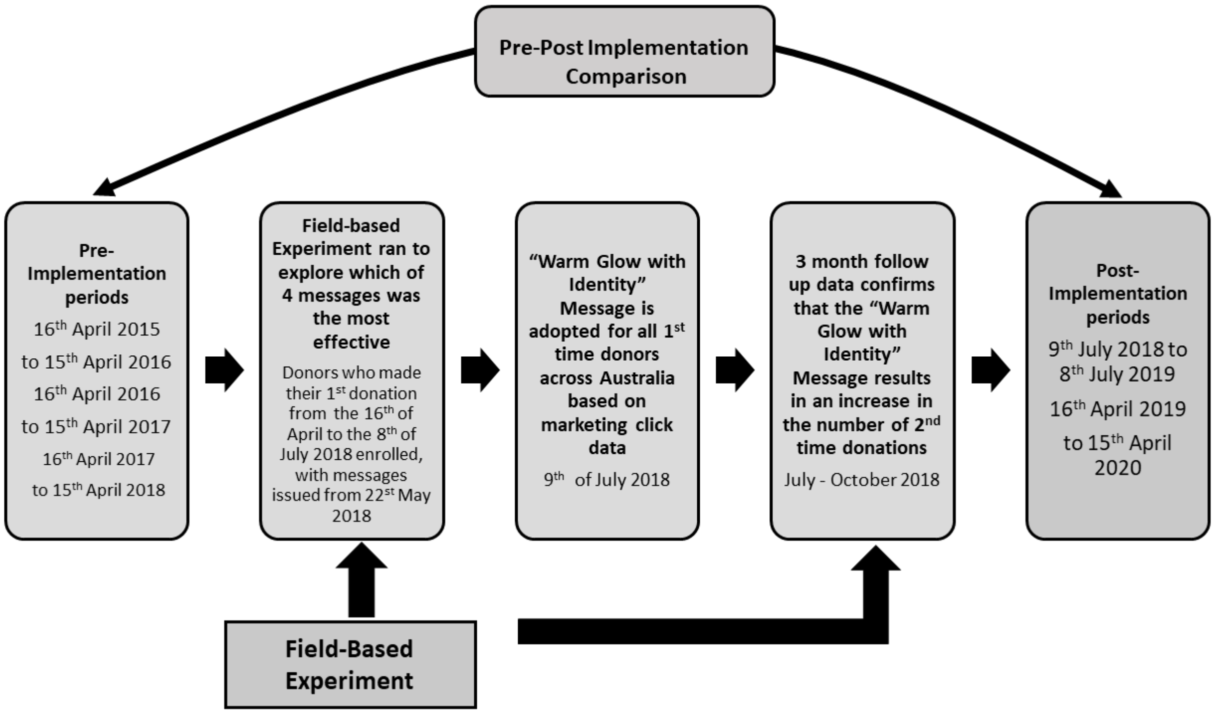

Supplementary Figure 10: *Timelines for Field Experiment and Implementation Analysis*

Supplementary Files S9: Q-Q Plots for Study 5

Supplementary Figures 11a (time 1) and Supplementary 11b (time 2) below provide the Q-Q plots for experienced warm-glow from study 5. There is some evidence of non-normality.

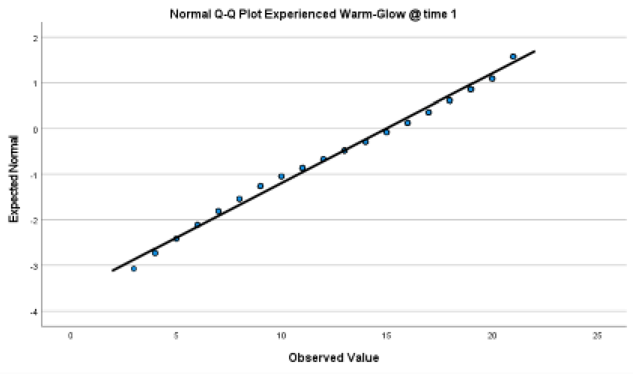

Supplementary Figure 11a

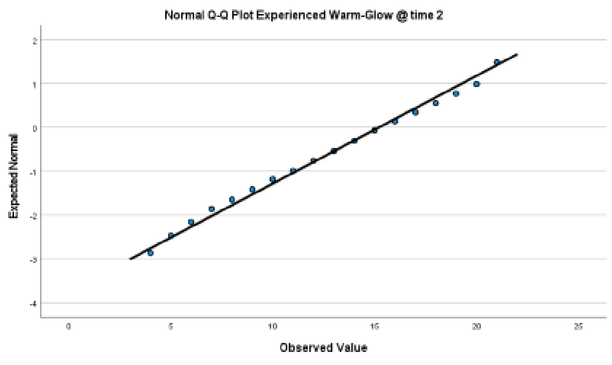

Supplementary Figure 11b

References

Carpenter, J. The shape of warm glow: Field experimental evidence from a fundraiser *J Econ Behav Org* 191 555-574 (2021).

Clark, C. F., Kotchen, M. J. & Moore, M. R. Internal and external influences on pro-environmental behavior: Participation in a green electricity program. *J. Environ. Psychol*, ***23***, 237–246 (2003).

Crumpler, H. & Grossman, P. J. An experimental test of warm glow giving. *J Pub Econ.* **92**, 1011-1021. (2008).

Evans, R. & Ferguson, E. Defining and measuring blood donor altruism: a theoretical approach from biology, economics and psychology. *Vox Sang.* **106**, *118-12* (2014).

Ferguson, E., & Cox, T. (1993). Exploratory factor analysis A user's guide. *Int J Select Assess **1***, 84 – 94 (1993)

Ferguson, E. & Lawrence, C. Altruistic and warm-glow motivations: variation by blood donor career. *Testing, Psychomets., Methodol. Appl. Psychol.* **29**, 639-651 (2019).

Ferguson, E. & Flynn, N. (2016). Moral relativism as a disconnect between behavioural and experienced warm glow. *J. Econ. Psychol.* **56**, 163-175 (2016).

Ferguson, E., Atsma, F., de Kort, W. & Veldhuizen, I. Exploring the pattern of blood donor beliefs in first time, novice and experienced donors: differentiating reluctant altruism, pure altruism, impure altruism and warm-glow *Transfusion*, **52**, 343-355**.** (2012).

Ferguson, E., Farrell, K. & Lawrence, C. Blood donation is an act of benevolence than altruism. *Health Psychol.* **27**, 327-336 (2008).

Ferguson, E., Murray, C. & O'Carroll, R. E. Blood and organ donation: Health impact, prevalence, correlates and interventions. *Psychol Health*, **34**, 1073-1104 (2019).

Ferguson, E., Taylor, M., Keatley, D., Flynn, N. & Lawrence, C. Blood donors' helping behavior is driven by warm glow more evidence for the blood donor benevolence hypothesis. *Transfusion*, **52**, 2189-2200 (2012).

Hartmann, P, Eisend, M., Apaolaza, V. & D'Souza, C. Warm glow vs. altruistic values: How important is intrinsic emotional reward in proenvironmental behavior? *J. Environ. Psychol.* **52**:43-55 (2017).

Konow, X. Mixed feelings: Theories of and evidence on giving. *J. Pub. Econ.* 94, 3279-297 (2010).

Sweegers, M. G. Twisk, J. W. R., Quee, F. A., Ferguson, E. & van den Hurk, K. Whole blood donors' post-donation symptoms diminish quickly but are discouraging: results from 6-day symptom diaries *Transfusion.* **61**, 811-821 (2021).

Taufik, D., Bolderdijk, J. & Steg L. Acting green elicits a literal warm glow. *Nat. Clim. Chang.* **5**, 37-40. (2015).

van der Linden., S. Warm glow is associated with low- but not high-cost sustainable behaviour. *Nat. Sustain.* **1,** 28–30 (2018).
