# Supplementary material for: Warming up cool cooperators
Source: Nat Hum Behav. 2023 Sep 14;7(11):1917–32. doi: 10.1038/s41562-023-01687-6 (PMC10663147; doi:10.1038/s41562-023-01687-6)
Supplement: Supplementary file 2 — Reporting Summary [file 41562_2023_1687_MOESM2_ESM.pdf]

nature research

Corresponding author(s): Eamonn Ferguson

Last updated by author(s): Jun 21, 2023

Reporting Summary

Nature Research wishes to improve the reproducibility of the work that we publish. This form provides structure for consistency and transparency in reporting. For further information on Nature Research policies, see our Editorial Policies and the Editorial Policy Checklist.

Statistics

For all statistical analyses, confirm that the following items are present in the figure legend, table legend, main text, or Methods section.

n/a	Confirmed	
☐	☒	The exact sample size (*n*) for each experimental group/condition, given as a discrete number and unit of measurement
☐	☒	A statement on whether measurements were taken from distinct samples or whether the same sample was measured repeatedly
☐	☒	The statistical test(s) used AND whether they are one- or two-sided<br>*Only common tests should be described solely by name; describe more complex techniques in the Methods section.*
☐	☒	A description of all covariates tested
☐	☒	A description of any assumptions or corrections, such as tests of normality and adjustment for multiple comparisons
☐	☒	A full description of the statistical parameters including central tendency (e.g. means) or other basic estimates (e.g. regression coefficient) AND variation (e.g. standard deviation) or associated estimates of uncertainty (e.g. confidence intervals)
☐	☒	For null hypothesis testing, the test statistic (e.g. F, t, r) with confidence intervals, effect sizes, degrees of freedom and P value noted<br>*Give P values as exact values whenever suitable.*
☒	☐	For Bayesian analysis, information on the choice of priors and Markov chain Monte Carlo settings
☒	☐	For hierarchical and complex designs, identification of the appropriate level for tests and full reporting of outcomes
☐	☒	Estimates of effect sizes (e.g. Cohen's *d*, Pearson's *r*), indicating how they were calculated

Our web collection on statistics for biologists contains articles on many of the points above.

Software and code

Policy information about availability of computer code

Data collection	eProgesia 5.03 (http://www.mak-system.com/blood-centers/), Prolific UK, (https://www.prolific.co/), Qualtrics (https://www.qualtrics.com)
Data analysis	IBM SPSS version 26 & 27, Psychometrica, Zumastat 4.0, MPlus_8.4, Stata SE 17, PROCESS 4.0, Comprehensive Meta Analysis v2

For manuscripts utilizing custom algorithms or software that are central to the research but not yet described in published literature, software must be made available to editors and reviewers. We strongly encourage code deposition in a community repository (e.g. GitHub). See the Nature Research guidelines for submitting code & software for further information.

Data

Policy information about availability of data

All manuscripts must include a data availability statement. This statement should provide the following information, where applicable:

- Accession codes, unique identifiers, or web links for publicly available datasets
- A list of figures that have associated raw data
- A description of any restrictions on data availability

The authors declare that all data supporting the findings of this study are available within the paper, supplementary files and at Ferguson, Eamonn, Barbara Masser, Carley N Gemelli, Lisa A Williams, Anne van Dongen, Amy Rozsa, Claire Lawrence, et al. 2022. “Warming Up Cool Cooperators.” OSF. July 27. osf.io/ps5kb.

Field-specific reporting

Please select the one below that is the best fit for your research. If you are not sure, read the appropriate sections before making your selection.

☐ Life sciences ☒ Behavioural & social sciences ☐ Ecological, evolutionary & environmental sciences

For a reference copy of the document with all sections, see nature.com/documents/nr-reporting-summary-flat.pdf

Behavioural & social sciences study design

All studies must disclose on these points even when the disclosure is negative.

Study description

We report results from six studies.

Study 1:We report a field-based experiment where we randomly allocate 5,821 1st time blood donors to one of four active message conditions based on warm-glow or impure altruism messages and a pure no-message control (n 5,801 took part after exclusions). We explored the moderation of messages by booking or return attendance. We collected quantitative data on the number who returned and actually attend to make a donation 3 months later.

Study 2: We confirmed the field-based experimental findings in a large-scale implementation trial where we compared the return rate to attend for a 2nd donation in the population of all new donors in Australia who had made their first donation in one of three one-year time windows prior to the warm-glow message being implemented (Ns = 90,317, 93,430, & 89,606), with the same return rates made by all new donors in Australia in one of two one-year time windows after the warm-glow message had been implemented (Ns = 81,766 & 88,551). We collected quantitative data of the number who returned and actually attend to make a donation 3 months later.

Study 3: A quantitative cross-sectional survey to evaluate whether booking was associated with self-reported warm-glow or pure-altruism. (n=716 first-time donors). We used self-reported survey items to assess anticipated warm-glow, negative affect, calmness and pure-altruism, booking status was recorded from donor records.

Study 4: Quantitative survey of n=1,124 donors. We examined 4 groups of donors by crossing donor status (first-time, novice) with donor type (plasma, blood) and explored the association between booking, self-reported warm-glow, and return behaviour. We collected quantitative data of the number who returned and actually attend to make a donation 3 months later, we assessed warm-glow by self-reports and booking status was recorded from donor records.

Study 5: Longitudinal survey at two-time points. We investigated the temporal stability of warm-glow among n=932 first-time donors by measuring warm-glow at two-time points (baseline; 12-week follow-up) and the effects of warm-glow and booking on return behaviour. We collected quantitative data of the number who returned and actually attend to make a donation 3 months later, we assessed booking status from donor records..

Study 6 was an online quantitative experiment (n = 1, 592) to validate the warm-glow and impure-altruism messages to be used in the field-based experiment. We collected quantitative data from self-reported data.

Research sample

Study 1: The field-based experiment was conducted on a sample of 5,821 1st time blood donors with 5,801 taking part after deferral criteria were applied. The initial sample of 5,821 (Mage = 31.98, [Sd = 11.96], % female = 60.7%) were new whole-blood donors from across Australia. After twenty donors were excluded (twelve due to email bounces and 8 due to being permanently deferred from donating blood) after their initial donation, the final sample was n=5,801 (Mage = 31.92, [Sd = 11.95], % female = 60.7%). This sample was chosen as our study focus was on increasing the conversion rate to make a second donation in first-time donors. Compared to the average age (32.5 years) and percentage of women (56%) for all 1st time whole blood donors in the three pre-implementation windows in study 2, this sample was generally representative.

Study 2: For the implementation trial we compared the return rate for a 2nd donation in the population of all new donors in Australia who had made their first donation in one of three one-year time windows prior to the warm-glow message being implemented (Ns = 90,317, 93,430, & 86,606), with the same return rates made by all new donors in Australia in one of two one-year time windows after the warm-glow message had been implemented (Ns = 81,766 & 88,551).The 3-year pre-implementation period covered 3 twelve-month time windows: beginning on the 16th of April to the 15th of April for years (1) 2015-2016 (n = 90,317; Mage = 32.0, [Sd = 13.6], % women = 55%, n = 49,778), (2) 2016-2017 (n = 93,430; Mage = 32.5, [Sd = 13.4], % women = 56%, n = 52,539), and (3) 2017-2018 (n = 86,606; Mage = 33.1, [Sd = 13.0], % women = 57%, n = 49,531), and the post-implementation period covered two slightly overlapping time windows: (1) 9th of July 2018 - 8th of July 2019 (n = 81,766; Mage = 34.9, [Sd = 13.5], % female = 57%, n = 46,689) and (2) 16th of April 2019 - 15th of April 2020 (n = 85,551; Mage = 34.3, [Sd = 13.2], % women = 57%, n = 50,480). This sample was chosen as our study focus was on increasing the conversion rate to make a second donation in first-time donors. This is the full population of all 1st-time donors

Study 3: Seven-hundred and sixteen first-time donors took part (Mage = 41.20, Sd = 14.85, % women = 65.6%, n women = 634). Sampling was targeted to ensure that the gender balance and age (18-69: donors over 70 were excluded) distribution was representative of the donor population. This sample was chosen as our study focused on exploring the links between self-reported warm-glow and booking status in first-time donors and this sample had items that matched the type of items that had been used previously to assess self-reported warm-glow and also sample first-time donors. In the nearest post-implementation time window (2019-2020) the average age was 34.3 with 57% women. Thus, the current sample was older and had a higher percentage of women.

Study 4: In total, 1,124 donors (Mage = 30.27, Sd = 11.51, % women = 55.2%, n = 621 women) participated. Of these, 401 were first-time whole-blood donors (Mage = 30.38, Sd = 11.879; % women = 54.6%, n = 219 women), 208 first-time plasma donors (Mage = 30.07, Sd = 11.50, % women 50.5%, n = 106 women), 379 were novice whole-blood donors (Mage = 29.61, Sd 11.49, % women = 60.4%, n = 229 women) and 136 novice plasma donors (Mage = 30.53, Sd = 10.75; % women = 49.3%, n = 67 women). This sample was chosen as our study focused on exploring the links between self-reported warm-glow, booking status and return behaviour and this study contained assessments of all of these. We also wished to explore the effects of first-time vs novice (more experienced donors) and type of donation (whole blood vs plasma) and this study contained data on all of these. The sample was representative in terms of the percentage of women but slightly younger than the average age (32.5 years) for 1st time whole blood donors in the three pre-implementation windows in study 2.

Study 5: Nine-hundred and thirty-two first-time donors who completed the survey provided warm-glow responses at waves 1 and 4 (Mage = 36.36, Sd = 14.02, % women = 70.3%, n = 655 women). This sample was chosen as our study focused on exploring the temporal stability of warm-glow in first-time donors, and in this study, warm-glow was assessed at two-time points. The study also had data on booking status and behaviour and this study also addressed the effects of booking on the stability of warm-glow and explored the prediction of behaviour from warm-glow and booking status. Compared to the average age (32.5 years) and percentage of women (56%) for 1st time whole blood donors in the three pre-implementation windows in study 2, this sample was slightly older and had a higher percentage of women.

Study 6: A total of 1,592 participants were recruited through Prolific (https://www.prolific.co/) (Mage = 36.47, Sd = 13.00, % women = 50.2%, n women = 795). This sample was chosen as our study was designed to explore the validity of the messages used in studies 1 and 2 in the general population. The sample is representative of 50.2% women compared to 51% of the population, but slightly younger at 36.47 years compared to the UK population average of 40.4 years.

Sampling strategy

Study 1: For the field-based experiment a sample of 5,821 new whole blood donors with A-, A+, O- and O+ blood types across Australia, who made their first whole blood donation six weeks previously, were recruited using the following criteria: (1) made their first whole blood donation six weeks ago, and (2) had not donated previously. For each week of the field-based experiment, all donors who met these eligibility criteria were selected and randomly allocated to conditions. The whole population meeting the sampling eligibility criteria were included. This process continued until the pre-determined numbers per condition were achieved. Sample size was determined as follows. As warm-glow has a small effect on predicted blood donor attendance, to achieve 80% power to detect a small effect with an alpha of .05 in a simple regression model with 8 predictors (e.g., message type and identity prime conditions, age, sex, blood group, booking status, and terms for the interaction of booking status with experimental conditions), 757 donors per arm are needed. Thus, we aimed for 1,000 new donors per arm to allow for ad-hoc exploratory analyses.

Study 2 For the implementation study all new donors in Australia across three pre- implementation time windows and 2 post-implementation time windows. We sampled the whole target population of first-time donors in Austrailia across three pre-implementation periods and two pots-implementtaion periods.

Study 3: 20,000 donors were contacted with an even split across the 3 groups; (1) first-time donors (first donated after February 2020), (2) existing eligible donors who had donated after February 2020, and (3) existing eligible donors who had not donated since February 2020. Sampling was targeted to ensure that the gender balance and age (18-69: donor over 70 were excluded) distribution was representative of the donor population. Nine-hundred and sixty-six first-time donors were recruited. Of these 250 1st-time donors started the survey but did not complete the majority of measures including the measure of adjectives used to assess warm-glow. As 250 did not complete the survey (non-completers) but started it, and 716 did (completers), there is a potential for selection bias. Indeed, completer status is predicted by having had a COVID-19 test ($\chi2$ (1) = 3.373, p = .066, ϕ = .061) with those who had a COVID-19 test more likely to be completers, and being older (M = 41.20, Sd = 14.85 vs M = 34.14, Sd = 14.33; t (964) = 6.531, p =.000; DCohen = 0.421). Completer status was not predicted by gender ($\chi2$ (1) = 0.020, p = .887, ϕ = -.005). Rebooking status was not predicted by being a non-completer or completer ($\chi2$ (1) = 1.151, p = .283, ϕ = .035) nor as a function of having had a COVID-19 test ($\chi2$ (1) = 3.315, p = .069, ϕ = .061). To account for any potential selection bias we completed a sensitivity analysis using a Heckman Probit Selection model with COVID-19 test, gender and age as predictors f selection bias. There was no evidence of selection bias. This study constituted a secondary analysis of these data. There was no data on which to base a power calculation. However, if we assume a small effect size then we would need 363 donors who booked and 363 who did not (n = 726) with a power of .80 and α = .05. In our sample 398 booked and 329 did not. As such, we had a sufficient sample size to detect a small effect.

Study 4: New (1st donation) and novice (1 or 2 previous donations and no plasma donations for whole blood donors, and 1 or 2 previous plasma donations and less than 3 whole blood donations for plasma donors) donors who were either whole blood or plasma donors were recruited by convenience sampling in donor centres, at three large urban Australian Red Cross Lifeblood (Lifeblood) centres (Brisbane Donor Centre, Sydney Town Hall and Melbourne Mt Waverley) from April 2016 to October 2017. A total sample of 1,153 donors were recruited. Of these, 441 were 1st time whole blood donors, 218 1st time plasma donors, 385 were novice whole blood donors and 136 novice plasma donors. Overall, there were 354 plasma donors and 799 whole blood donors There were missing data on warm-glow (n = 26), age (n = 1) and gender (n = 2). Once these were removed the final sample size was 1,124. Of these, 401 were 1st time whole blood donors, 208 1st time plasma donors, 379 were novice whole blood donors and 136 novice plasma donors. Overall, there were 344 plasma donors and 780 whole blood donors. There was no data on which to base a power calculation. However, if we assume a small effect size then we would need 363 donors who booked and 363 who did not (n = 726) with a power of .80 and α = .05. In our sample 781 booked and 329 did not. As such, we had a sufficient sample size to detect a small effect.

Study 5: All 25,428 first-time whole blood donors were invited to participate in the study between January 2017 and September 2017. 4,472 first-time donors were recruited of these data were missing from 500 participants on intrinsic regulation (warm-glow) at wave 1, leaving complete data on 3,972. Of the 4,472, 136 donors were actively deferred from donating for medical and life-style reasons. Those deferred were more likely to be women ($\chi2$ (1) = 18.296, p = .000, ϕ = .064) but did not vary by age (M deferred = 33.88, Sd = 12.83 vs M not deferred = 35.20, Sd = 13.57: t (4470) = 1.119, p =.263; DCohen = 0.033). Those deferred were not significantly different from those not-deferred on warm-glow at wave 1 (M deferred = 15.06, Sd = 4.34 vs M not deferred = 15.18, Sd

= 4.09: t (3970) = 0.315, p =.753; DCohen = 0.009) or wave 4 (M deferred = 15.48, Sd = 4.09 vs M not deferred = 15.21, Sd = 4.07: t (955) = -0.329, p =.743; DCohen = -0.021). However, as deferral status influenced the decision to re-book (χ2 (1) = 5.571, p = .016, ϕ = -.036), with those deferred less likely to rebook, we excluded those who were actively deferred from the analyses. After removal of those actively deferred there were 4336 1st-time donors at wave 1. At wave 4 a total of 932 completed the survey providing data on warm-glow. Those who completed the surveys providing data on warm-glow at waves 1 and 4 were significantly older (M Wave 1 only = 34.88, Sd = 13.39 vs M Waves 1 & 2 = 36.27, Sd = 14.09: t (4334) = -2.863 p =.004; DCohen = -0.087) and less likely to be male (χ2 (1) = 8.231, p = .004, ϕ = -.044) than those who completed only wave 1, however, they did not significantly vary on warm-glow at wave 1 (M Time 1 only = 15.25, Sd = 4.06 vs M time1 & 2 = 14.97, Sd = 4.18: t (3849) = 1.892, p =.059; DCohen = 0.061). Furthermore, warm-glow at wave 4, for those who completed both surveys (M = 15.21, Sd = 4.06) was not significantly different from warm-glow at wave 1 (M = 15.25, Sd = 4.06) in those who just completed the survey at time 1 (t (one-sample (931) = -0.320 p =.749; DCohen = -0.021). As such, there is no evidence that initial levels of warm-glow influenced who remained in the study from waves 1 to waves 4. However, we controlled for age and gender in these analyses. There was no data on which to base a power calculation. However, if we assume a small effect size then we would need 363 donors who booked and 363 who did not (n = 726) with a power of .80 and α = .05. In our sample 612 booked and 320 did not. As such, we had a sufficient sample size to detect a small effect

Study 6: Participants (aged 18 to 80 from the UK) were recruited through Prolific (https://www.prolific.co/) to an experiment hosted on Qualtrics (https://www.qualtrics.com/uk/). A total of 1,592 were recruited. There were 266 participants in the BAU/Control, 273 in the 'identity-only', 263 in the 'warm-glow-only', 256 in the 'warm-glow-plus-identity', 279 in the 'impure-altruism-only', and 255 in the 'impure-altruism-plus-identity' conditions. Ferguson, Farrell, and Lawrence 64 report a Cohen's d of 0.344 for positive ratings of warm-glow vs altruistic messages for blood donation. Achieving 80% power to detect a small effect with an alpha of .05 for a 2 (warm-glow: yes; no) ⍰ 2 (identity: yes; no) design requires 265 per condition. For a series of pairwise comparisons of experimental groups, detecting a small effect with a Cohen's d of 0.344 requires 135 participants per condition.

Data collection

We collected data on verified donor attendance for studies 1, 2, 4 and 5. This was achieved in a blinded manner, with data analysts who extracted the attendance data blind to the experimental condition that the donor was assigned to. We collected verified data on booking status in studies 1, 2, 3, 4, and 5.

We collected survey data on experienced warm-glow in studies 3, 4, 5, and afforded warm-glow in 6, and pure-altruism in study 3.

In study 3 we assessed 'anticipated warm-glow' using adjectives of the type that have been used to assess warm-glow in previous research. Specifically, donors were asked: "What do you think donating blood in the future during the coronavirus pandemic would be like?" to which donors responded using 12 adjectives [Unrewarding, Pointless, Displeasing, Negative, Stressful, Unsatisfying, Rewarding, Worthwhile, Pleasing, Positive, Relaxing, Satisfying], each measured on a 4-point scale (1 = Not at all, 2 = Slightly, 3 = Moderately and 4 = Extremely). Of these adjectives, 'Rewarding', 'Worthwhile', 'Pleasing', 'Positive', and 'Satisfying' represent the type of adjectives that have been used to assess warm-glow in previous research [e.g., 24]. 'Pure Altruism' was assessed with two items concerning the donor's motivation for their last donation ("To help someone in need" and "It's important for society"), each measured on a 5-point scale (1 = Not important at all, 2 = Slightly Important, 3 = Moderately Important, 4 = Very Important, 5 = Extremely Important).

In studies 4 and 5 experienced warm-glow was assessed using the intrinsic regulation subscale the Blood Donor Identity Survey (BDIS) which comprises 3 items ("I enjoy donating blood", "For me, being a blood donor means more than just donating blood", and "Blood donation is an important part of who I am") with each item measured on a 7-point scale (1 = Not at all True, 7 = Very True).

We collected data, in study 6, on the perception of the messages to be used in the field-based experiment (study 1) with respect to afforded warm-glow, recipient vs donor focus and provision of the public good (blood supply). 'Afforded Donor vs Recipient Focus' was calculated as the average of 2 items ("To what extent do you think that the message focuses on the blood donor, the recipient of blood or both?" and "To what extent do you think that the message focuses on the emotions of the blood donor, the recipient of blood or both?") measured on a scale from -50 (primarily on the blood donor) to +50 s (primarily on the recipient of blood) with a midpoint of 0 (equally on both the donor and recipient). 'Afforded Warm-Glow' was calculated as the sum of 2 items ("The message makes me feel that donating blood is personally rewarding in itself" and "The message makes me feel that donating blood would make me feel like a good person) measured on a 7-point scale (1 = "not at all" to 7 = "completely"). 'Afforded Focus on Maintaining the Blood Supply' was assessed with a single item ("The message makes me feel that donating blood would ensure that there is enough blood for all who need it.") measured on a 7-point scale (1 = "not at all" or 7 = "completely.").

Others present when data was collected: For study 1 donors received a message there may have been other present when they read it but we do not have data on this. For study 2 this was aggregated population data on booking status and return behaviour other donors and staff may have been present when donors booked. Study 3 was an online survey (Qualtrics) and it is assumed that donors complete this independently. For study 4 data was collected using an electronic tablet in a donor centre with other blood donors and blood collection staff present. For study 5 data was collected using an online questionnaire (Qualtrics) and it is assumed that donors complete this independently. For study 6 data was collected using an online questionnaire (Qualtrics) and it is assumed that donors complete this independently.

Timing

Study 1: The field experiment ran from the 22nd of May 2018 to the 8th of July with the 3 month follow up on attendance completed by the end of Nov 2018.

Study 2: For the implementation study the following time windows were analysed. Pre-implementation period covered three time windows consisting of the 16th of April to the 15th of April for years (1) 2015-2016, (2) 2016-2017 and (3) 2017-2018 and the post-implementation period covered two slightly overlapping time windows (1) the 9th of July 2018 to the 8th of July 2019 and (2) the 16th of April 2019 to the 15th of April 2020.

Study 3:Donors were sent a de-identified online survey invitation between the 12th of August and the 24th of September 2020. A reminder was sent on the 27th of August 2020. Data were extracted on the 24th of September 2020

Study 4: The survey took place between April 2016 to October 2017.

Study 5: The survey took place between January 2017 and September 2017.

Study 6: The online survey took place on the 24th of March 2021, and all data were collected on that day.

Data exclusions

Study 1: For the field experiment 20 participants were excluded, of which 12 had bounced emails and 8 with permanent deferrals (evidence of blood-borne infection). These were not pre-determined and analyses showed that they were evenly distributed across the study conditions.

Study 2: There were no exclusions in the implementation trial.

Study 3: Nine-hundred and sixty-six first-time donors were recruited. Of these 250 1st-time donors started the survey but did not complete the majority of measures including the measure of adjectives used to assess warm-glow. As 250 did not complete the survey (non-completers) but started it, and 716 did (completers), there is a potential for selection bias. To account for any potential selection bias we completed a sensitivity analysis using a Heckman Probit Selection model with COVID-19 test, gender and age as predictors f selection bias. There was no evidence of selection bias.

Study 4: A total sample of 1,153 donors were recruited. There were missing data on warm-glow (n = 26), age (n = 1) and gender (n = 2). Once these were removed the final sample size was 1,124. Of these, 401 were 1st time whole blood donors, 208 1st time plasma donors, 379 were novice whole blood donors and 136 novice plasma donors. Overall, there were 344 plasma donors and 780 whole blood donors.

Study 5: Of the 4,472, 136 donors were actively deferred from donating for medical and life-style reasons.. After removal of those actively deferred there were 4336 1st-time donors at wave 1. At wave 4 a total of 932 completed the survey providing data on warm-glow. There is no evidence that initial levels of warm-glow influenced who remained in the study from waves 1 to waves 4. However, we controlled for age and gender in these analyses.

Study 6: There were no exclusions.

Non-participation

Study 1: 20 donors were excluded (12 due to bounced emails, and 8 due to permanent deferrals) With respect to the initial randomization of 5,821 donors dropping to 5801 after the 20 exclusions. There was no evidence that exclusion systematically varied across arms or affect distributions of age and sex. Thus, there was no evidence for bias.

Study 2: There were no dropouts.

Study 3: Nine-hundred and sixty-six first-time donors were recruited. Of these 250 first-time donors started the survey but did not complete the majority of measures including the measure of adjectives used to assess warm-glow. As 250 did not complete the survey (non-completers) but started it, and 716 did (completers), there is a potential for selection bias. To account for any potential selection bias we completed a sensitivity analysis using a Heckman Probit Selection model and there was no evidence for bias.

Study 4: A total sample of 1,153 donors were recruited. Of these, 441 were first-time whole blood donors, ,218 first-time plasma donors, 385 were novice whole blood donors and 136 novice plasma donors. There were missing data on warm-glow (n = 26), age (n = 1) and gender (n = 2). Once these were removed the final sample size was 1,124.
.
Study 5: Of the 4,472 recruited donors, 136 donors were actively deferred from donating for medical and life-style reasons. After removal of those actively deferred there were 4,336 first-time donors at wave 1. At wave 4 a total of 932 completed the survey providing data on warm-glow. The other data were excluded doe to survey non-completion.

Study 6: There were 266 participants in the BAU/Control, 273 in the 'identity-only', 263 in the 'warm-glow-only', 256 in the 'warm-glow-plus-identity', 279 in the 'impure-altruism-only', and 255 in the 'impure-altruism-plus-identity' conditions. There were no dropouts.

Randomization

In study 6 participants were block-randomized by sex to each of the 6 conditions to ensure equal numbers of males and females For the field-based experiment (study 1), this was simple randomization. There was no randomization for the implementation study where we compared pre-and post-implantation time periods or for studies 3, 4 and 5 which were observational. Study 2 (implementation study) was based on aggregate data and we could not control for age and sex. However, these were consistent across the time window and by booking status so unlikely to have influence the pattern of results observed.

Reporting for specific materials, systems and methods

We require information from authors about some types of materials, experimental systems and methods used in many studies. Here, indicate whether each material, system or method listed is relevant to your study. If you are not sure if a list item applies to your research, read the appropriate section before selecting a response.

Materials & experimental systems

n/a	Involved in the study
☒	☐ Antibodies
☒	☐ Eukaryotic cell lines
☒	☐ Palaeontology and archaeology
☒	☐ Animals and other organisms
☐	☒ Human research participants
☒	☐ Clinical data
☒	☐ Dual use research of concern

Methods

n/a	Involved in the study
☒	☐ ChIP-seq
☒	☐ Flow cytometry
☒	☐ MRI-based neuroimaging

Human research participants

Policy information about studies involving human research participants

Population characteristics

Study 1: For the field-based experiment we have the following characteristics. In the control condition we had an n of 1162, with a mean age 32.31 (SD = 12.06) with a %female of 60.5. In the warm-glow-identity condition we had an n of 1164 with a mean age of 31.72 (SD = 11.78) and a %female of 60.8. In the warm-glow condition we had an n of 1164 with a mean age of 32.82 (SD = 12.03) and a %female = 60.4. In the positive affect identity condition we had an n of 1164 with a mean age of 31.92 (SD = 12.12) with a %female of 62.7. In the positive affect condition we had an n of 1167 with a mean age of 31.66 (SD = 11.81) with a %female of 59.0. The majority of the sample had blood type O+ (45.9%), followed by A+ (36.9%), and O- (10.7%), A- (6.4%) and O (1%).

Study 2: For the implementation study the % female at each time window was as follows: Pre-implementation time window one (2015-2016: n = 90,317) the mean age was 32.0 (Sd = 13.6) and % female was 55%, time window two (2016-2017: n = 93,430) the mean age was 35.5 (Sd = 13.4) and the %female was 56%, and time window 3 (2017-2018: n = 86,606) the mean age was 33.1 (Sd = 13.0) and the %female was 57%. In the post-implementation period for time windows one (9th of July 2018 to the 8th of July 2019: n = 81,766) the mean age was 34.9 (Sd = 1354) and the %female was 56%, and for time window two (6th of April 2019 to the 15th of April 2020: n = 88.551) the mean age was 34.3 (Sd = 13.2) and the %female was 57%. For the aggregate pre-implementation mean age Ws 35.5 (Sd = 13.3: n = 270,353) and the post-implementation period the mean age was 34.6 (Sd = 13.3: n = 170, 317). As these are aggregate data to examine the effects of age and sex on the aggregate results we re-ran the aggregated analysis on percentage return rates adjusted for aggregate age and sex (percentage women). We obtained estimates of age- and sex-adjusted percentage return rates from a simple general linear model (n = 10). This simple adjustment for age and sex does not alter the pattern of results in terms of the key interaction and the effect of the warm-glow-plus-identity message on return rates for those who do not book.

Study 3: Nine-hundred and sixty-six first-time donors were recruited (mean age = 39.37 years, Sd = 15.03 years; 66% female with 23.7% having had a COVID-19 test). Of these 250 1st-time donors started the survey but did not complete the majority of measures including the measure of adjectives used to assess warm-glow. As 250 did not complete the survey (non-completers) but started it, and 716 did (completers), there is a potential for selection bias. Indeed, completer status is predicted by having had a COVID-19 test (χ^2 (1) = 3.373, p = .066, ϕ = .061) with those who had a COVID-19 test more likely to be completers, and being older (M = 41.20, Sd = 14.85 vs M = 34.14, Sd = 14.33; t (964) = 6.531, p =.000; DCohen = 0.421). Completer status was not predicted by gender (χ^2 (1) = 0.020, p = .887, ϕ = -.005). Rebooking status was not predicted by being a non-completer or completer (χ^2 (1) = 1.151, p = .283, ϕ = .035) nor as a function of having had a COVID-19 test (χ^2 (1) = 3.315, p = .069, ϕ = .061). To account for any potential selection bias we completed a sensitivity analysis using a Heckman Probit Selection model with COVID-19 test, gender and age as predictors of selection bias.

Study 4: A total sample of 1,153 donors were recruited. Of these, 441 were 1st time whole blood donors (M age = 30.47, Sd = 11.86, n = 413; % female = 55.6%, n = 412), 218 1st time plasma donors (M age = 30.95, Sd = 11.30, n = 218; % female 50.5%, n = 218), 385 were novice whole blood donors (M age = 29.52, Sd 11.47, n = 385; % female = 60.8%, n = 385) and 136 novice plasma donors (M age = 30.5, Sd = 10.75 n = 136; % female = 49.3%, n = 136;). Age did not vary significant across these groups (F (3, 1148) , = 0.860, p = .461, ?p2 = .002) but gender did (χ^2 (3) = 8.73, p = .033, ϕ = .087). Overall, there were 354 plasma donors (M age = 30.79, Sd = 11.19, n = 345, % female = 50.0%, n = 354) and 799 whole blood donors (M age = 30.01, Sd = 11.64, n = 798; % female = 57.4%, n = 797) who did not differ by age (t (1150) = 1.06, p =.289; DCohen = 0.063) but did by gender (χ^2 (1) = 6.50, p = .011, ϕ = -.075). There were 632 1st-time donors (M age = 30.64, Sd = 11.67, n = 631; % female = 53.8%, n = 630) and 521 novice donors (M age = 29.79, Sd = 11.26, n = 521; % female = 57.5%, n = 521) who did not differ by age (t (1150) = 1.25, p =.212; DCohen = 0.074) or gender (χ^2 (1) = 1.81, p = .178, ϕ = -.040). There were missing data on warm-glow (n = 26), age (n = 1) and gender (n = 2). Once these were removed the final sample size was 1,124. Of these, 401 were 1st time whole blood donors (M age = 30.38, Sd = 11.879; % female = 54.6%, n = 219 women), 208 1st time plasma donors (M age = 30.07, Sd = 11.50, % female 50.5%, n = 106 women), 379 were novice whole blood donors (M age = 29.61, Sd 11.49, % female = 60.4%, n = 229 women) and 136 novice plasma donors (M age = 30.53, Sd = 10.75; % female = 49.3%, n = 67 women). Neither age (F (3, 1120) , = 0.787, p = .501, ?p2 = .002) nor gender = (χ^2 (3) = 7.68, p = .053, ϕ = .083) significant varied across these groups. Overall, there were 344 plasma donors (M age = 30.85, Sd = 11.19, % female = 50.3%, n = 173 women) and 780 whole blood donors (M age = 30.01, Sd = 11.65, % female = 57.4%, n = 797) who did not differ by age (t (1122) = 1.14, p =.254; DCohen = 0.068) but did by gender (χ^2 (1) = 4.93, p = .026, ϕ = -.066). There were 609 1st-time donors (M age = 30.62, Sd = 11.69; % female = 53.4%, n = 325 women) and 515 novice donors (M age = 29.85, Sd = 11.30; % female = 57.5%, n = 296 women) who did not differ by age (t (1122) = 1.11, p =.267; DCohen = 0.066) or gender (χ^2 (1) = 1.81, p = .178, ϕ = -.040).

Study 5: 4,472 first-time donors were recruited (mean age = 35.16 years, SD = 13.55 years; 68% female), of these data were missing from 500 participants on intrinsic regulation (warm-glow) at wave 1, leaving complete data on 3,972. We did not

impute these missing data as data were missing on all items pertaining to warm-glow and the missing percentage was high (11%). Of the 4,472, 136 donors were actively deferred from donating for medical and life-style reasons. Those deferred were more likely to be women (χ2 (1) = 18.296, p = .000, ϕ = .064) but did not vary by age (M deferred = 33.88, Sd = 12.83 vs M not deferred = 35.20, Sd = 13.57: t (4470) = 1.119, p =.263; DCohen = 0.033). Those deferred were not significantly different from those not-deferred on warm-glow at wave 1 (M deferred = 15.06, Sd = 4.34 vs M not deferred = 15.18, Sd = 4.09: t (3970) = 0.315, p =.753; DCohen = 0.009) or wave 4 (M deferred = 15.48, Sd = 4.09 vs M not deferred = 15.21, Sd = 4.07: t (955) = -0.329, p =.743; DCohen = -0.021). However, as deferral status influenced the decision to re-book (χ2 (1) = 5.571, p = .016, ϕ = -.036), with those deferred less likely to rebook, we excluded those who were actively deferred from the analyses. After removal of those actively deferred there were 4336 1st-time donors (M age = 35.20 years, Sd = 15.57 years; % female = 67%) at wave 1. At wave 4 a total of 932 completed the survey providing data on warm-glow. Those who completed the surveys providing data on warm-glow at waves 1 and 4 were significantly older (M Wave 1 only = 34.88, Sd = 13.39 vs M Waves 1 & 2 = 36.27, Sd = 14.09: t (4334) = -2.863 p =.004; DCohen = -0.087) and less likely to be male (χ2 (1) = 8.231, p = .004, ϕ = -.044) than those who completed only wave 1, however, they did not significantly vary on warm-glow at wave 1 (M Time 1 only = 15.25, Sd = 4.06 vs M time1 & 2 = 14.97, Sd = 4.18: t (3849) = 1.892, p =.059; DCohen = 0.061). Furthermore, warm-glow at wave 4, for those who completed both surveys (M = 15.21, Sd = 4.06) was not significantly different from warm-glow at wave 1 (M = 15.25, Sd = 4.06) in those who just completed the survey at time 1 (t (one-sample (931) = -0.320 p =.749; DCohen = -0.021). As such, there is no evidence that initial levels of warm-glow influenced who remained in the study from waves 1 to waves 4. However, we controlled for age and gender in these analyses.

Study 6: A total of 1,592 were recruited (mean age = 36.47, Sd = 13.00 female % = 52). 34% (n = 538) indicated that they had ever donated blood and 12.1% (n = 194) were current donors (donated within the last 2 years). There were 266 participants in the BAU/Control, 273 in the 'identity-only', 263 in the 'warm-glow-only', 256 in the 'warm-glow-plus-identity', 279 in the 'impure-altruism-only', and 255 in the 'impure-altruism-plus-identity' conditions. Neither age (F (5, 1583) , = 1.770, p = .116, ?p2 = .006), gender (χ2 (5) = 0.863, p = .973, ϕ = .023), ever donated blood (χ2 (5) = 8.918, p = .112, ϕ = .075) nor being a current blood donor (χ2 (5) = 1,221, p = .943, ϕ = .028) significantly varied by condition.

Recruitment

Study 1: For the field-based experiment the whole population of all 1st time donors who met the inclusion criteria were sampled for each week of the field-based experiment and randomly allocated to conditions. The whole population meeting the sampling eligibility criteria were included. This process continued until the pre-determined numbers per condition were achieved. As such, there is little scope for bias donor were sample and allocated to recieve a message based o the randomization schedule.A sample of 5,821 (Mage = 31.98, [Sd = 11.96], % female = 60.7%) new whole-blood donors from across Australia with A-, A+, O- and O+ blood types (this was based on the Australian Red Cross Lifeblood donor segmentation policy at that time. However, A and O positive and negative groups are the most prevalent, accounting for 86% of Australian blood donors) who had made their first whole-blood donation six weeks prior and had not donated previously in Australia were recruited. Twenty donors were excluded: twelve due to email bounces and 8 due to being permanently deferred from donating blood after their initial donation, leaving a final sample of n=5,801 (Mage = 31.92, [Sd = 11.95], % female = 60.7%.With respect to the initial randomization of 5,821 donors, age did not vary significantly across treatments (F (4, 5816) = 0.725, p = 0.575: DCohen = 0.059), nor did sex (⍰2 (4) = 3.542, p = 0.472: DCohen = 0.050). The majority of the sample had blood type O+ (45.9%), followed by A+ (36.9%), and O- (10.7%), A- (6.4%) and O (1%). The distribution of blood type did not vary by treatment (⍰2 (16) = 18.16, p = 0.314: DCohen = 0.112). The percentage who booked immediately post-donation was 37.7%, with 62.3% not booking while in the donor centre, and this did not vary by condition (⍰2 (4) = 4.163, p = 0.384: DCohen = 0.054). After the 20 donors were excluded (Supplementary Figure 8), donor age remained nonsignificant by condition (F (4, 796) = 0.796, p = 0.527: DCohen = 0.059), as did sex (⍰2 (4) = 3.536, p = 0.472: DCohen = 0.054). The majority of the sample had blood group O+ (46%), followed by A+ (36.9%), and O- (10.7%), A- (6.4%). The distribution of blood type did not vary by condition (⍰2 (12) = 14.62, p = 0.263; DCohen = 0.100). The percentage who booked was 37.7% with 62.3% not initially booking and this did not vary by condition (⍰2 (4) = 4.198, p = 0.380: DCohen = 0.054). Thus, the randomization by arm remained intact after the exclusions, as such there is no evidence of bias due to exclusions.

Study 2: For the implementation study all new donors in Australia across three pre implementation time windows and 2 post implementation time windows were selected. As the whole populations are sampled for the field-based experiment and the implementation study we feel there is little to no bias in the results especially as the findings from the field-based experiment are confirmed in the implementation study.

Study 3: 20,000 Australian donors were contacted with an even split across the 3 groups; (1) first-time donors (first donated after February 2020), (2) existing eligible donors who had donated after February 2020, and (3) existing eligible donors who had not donated since February 2020. Sampling was targeted to ensure that the gender balance and age (18-69: donor over 70 were excluded) distribution was representative of the donor population. A 10-15% response rate was expected and as such some self-selection bias is possible., however, the attained sample was representative. Donors were sent a de-identified online survey invitation between the 12th of August and the 24th of September 2020. A reminder was sent on the 27th of August 2020. Data were extracted on the 24th of September 2020. The survey was hosted on Qualtrics (https://www.qualtrics.com/). Nine-hundred and sixty-six first-time donors were recruited (mean age = 39.37 years, Sd = 15.03 years; 66% female with 23.7% having had a COVID-19 test). The low number of COVID-19 tests reflect the low rate of COVID-19 infection in Australia at the time. Of these 250 first-time donors started the survey but did not complete the majority of measures including the measure of adjectives used to assess warm-glow. As 250 did not complete the survey (non-completers) but started it, and 716 did (completers), there is a potential for selection bias. Indeed, completer status is predicted by having had a COVID-19 test (χ2 (1) = 3.373, p = .066, ϕ = .061) with those who had a COVID-19 test more likely to be completers, and being older (M = 41.20, Sd = 14.85 vs M = 34.14, Sd = 14.33; t (964) = 6.531, p = .000; DCohen = 0.421). Completer status was not predicted by gender (χ2 (1) = 0.020, p = .887, ϕ = -.005). Booking status was not predicted by being a non-completer or completer (χ2 (1) = 1.151, p = .283, ϕ = .035) nor as a function of having had a COVID-19 test (χ2 (1) =

3.315, p = .069, ϕ = .061). To account for any potential selection bias we completed a sensitivity analysis using a Heckman Probit Selection model with COVID-19 test, gender and age as predictor of selection bias.

Study 4: New (1st donation) and novice (1 or 2 previous donations and no plasma donations for whole blood donors, and 1 or 2 previous plasma donations and less than 3 whole blood donations for plasma donors) donors who were either whole blood or plasma donors were recruited by convenience sampling in donor centres, at three large urban Australian Red Cross Lifeblood (Lifeblood) centres (Brisbane Donor Centre, Sydney Town Hall and Melbourne Mt Waverley) from April 2016 to October 2017. There is room for self-selection bias due to the convenience sampling procedure. In total, 1,124 donors (Mage = 30.27, Sd = 11.51, % women = 55.2%, n = 621 women) participated. Of these, 401 were first-time whole-blood donors (Mage = 30.38, Sd = 11.879; % women = 54.6%, n = 219 women), 208 first-time plasma donors (Mage = 30.07, Sd = 11.50, % women 50.5%, n = 106 women), 379 were novice whole-blood donors (Mage = 29.61, Sd 11.49, % women = 60.4%, n = 229 women) and 136 novice plasma donors (Mage = 30.53, Sd = 10.75; % women = 49.3%, n = 67 women). In total, 1,124 donors (Mage = 30.27, Sd = 11.51, % women = 55.2%, n = 621 women) participated. Of these, 401 were first-time whole-blood donors (Mage = 30.38, Sd = 11.879; % women = 54.6%, n = 219 women), 208 first-time plasma donors (Mage = 30.07, Sd = 11.50, % women 50.5%, n = 106 women), 379 were novice whole-blood donors (Mage = 29.61, Sd 11.49, % women = 60.4%, n = 229 women) and 136 novice plasma donors (Mage = 30.53, Sd = 10.75; % women = 49.3%, n = 67 women). Thus there was some variability by age and sex, also the sampling design involved clustering. To account for this we controlled for age and sex and ran the statistical model with both standard error and error clustered within sample region. The results show that the results did not differ for the models with standard errors or clustered errors.

Study 5: All 25,428 first-time whole blood donors were invited to participate in the study between January 2017 and September 2017. All eligible donors were sent an email invitation asking them to participate in the study. A reminder email was sent after 5 days. Nine-hundred and thirty-two first-time donors who completed the survey provided warm-glow responses at waves 1 and 4 (Mage = 36.36, Sd = 14.02, % women = 70.3%, n = 655 women). All 25,428 first-time whole blood donors were invited to participate in the study between January 2017 and September 2017. 4,472 first-time donors were recruited (M age = 35.16, Sd = 13.55; % female = 68%). Thus there is a potential selection bias here. However, the sample was representative. Of these, data were missing from 500 participants on intrinsic regulation (warm-glow) at wave 1, leaving complete data on 3,972 participants. We did not impute these missing data as data were missing on all items pertaining to warm-glow and the missing percentage was high (11%). Of the 4,472 recruited donors, 136 donors were actively deferred from donating for medical and life-style reasons. Those deferred were more likely to be women ($\chi2$ (1) = 18.296, p = .000, ϕ = .064) but did not vary by age (M deferred = 33.88, Sd = 12.83 vs M not deferred = 35.20, Sd = 13.57: t (4470) = 1.119, p = .263; DCohen = 0.033). Those deferred were not significantly different from those not-deferred on warm-glow at wave 1 (M deferred = 15.06, Sd = 4.34 vs M not deferred = 15.18, Sd = 4.09: t (3970) = 0.315, p = .753; DCohen = 0.009) or wave 4 (M deferred = 15.48, Sd = 4.09 vs M not deferred = 15.21, Sd = 4.07: t (955) = -0.329, p = .743; DCohen = -0.021). However, as deferral status influenced the decision to book ($\chi2$ (1) = 5.571, p = .016, ϕ = -.036), with those deferred less likely to book, we excluded those who were actively deferred from the analyses. After removal of those actively deferred there were 4,336 first-time donors (M age = 35.20 years, Sd = 15.57 years; % female = 67%) at wave 1. At wave 4 a total of 932 completed the survey providing data on warm-glow. Those who completed the surveys providing data on warm-glow at waves 1 and 4 were significantly older (M Wave 1 only = 34.88, Sd = 13.39 vs M Waves 1 & 2 = 36.27, Sd = 14.09: t (4334) = -2.863 p = .004; DCohen = -0.087) and less likely to be male ($\chi2$ (1) = 8.231, p = .004, ϕ = -.044) than those who completed only wave 1, however, they did not significantly vary on warm-glow at wave 1 (M Time 1 only = 15.25, Sd = 4.06 vs M time1 & 2 = 14.97, Sd = 4.18: t (3849) = 1.892, p = .059; DCohen = 0.061). Furthermore, warm-glow at wave 4, for those who completed both surveys (M = 15.21, Sd = 4.06) was not significantly different from warm-glow at wave 1 (M = 15.25, Sd = 4.06) in those who just completed the survey at time 1 (t (one-sample (931) = -0.320, p = .749; DCohen = -0.021). As such, there is no evidence that initial levels of warm-glow influenced who remained in the study from waves 1 to waves 4. However, we controlled for age and gender in these analyses as these demonstrated systematic variation and potential for bias.

Study 6: Participants who lived in England and were aged between 18 and 80 and registered on Prolific we recruited until the target sample size was achieved. A total of 1,592 participants were recruited through Prolific (https://www.prolific.co/) (Mage = 36.47, Sd = 13.00, % women = 50.2%, n women = 795) with 34% (n = 538) indicating that they had ever donated blood and 12.1% (n = 194) being current donors (donated within the last 2 years). There were 266 participants in the BAU/Control, 273 in the 'identity-prime-only', 263 in the 'warm-glow-only', 256 in the 'warm-glow-plus-identity', 279 in the 'impure-altruism-only', and 255 in the 'impure-altruism-plus-identity' conditions. Recruitment was through Prolific (https://www.prolific.co/) to an experiment hosted on Qualtrics (https://www.qualtrics.com/uk/). All data were collected on the 24th of March 2021. Participants were block-randomized by sex to each of the 6 conditions. A total of 1,592 were recruited (Mage = 36.47, Sd = 13.00, % female = 52%). 34% (n = 538) indicated that they had ever donated blood and 12.1% (n = 194) were current donors (donated within the last 2 years). There were 266 participants in the BAU/Control, 273 in the 'identity-only', 263 in the 'warm-glow-only', 256 in the 'warm-glow-plus-identity', 279 in the 'impure-altruism-only', and 255 in the 'impure-altruism-plus-identity' conditions. Neither age (F (5, 1583) = 1.770, p = .116, ?p2 = .006), gender ($\chi2$ (5) = 0.863, p = .973, ϕ = .023), ever donated blood ($\chi2$ (5) = 8.918, p = .112, ϕ = .075) nor being a current blood donor ($\chi2$ (5) = 1,221, p = .943, ϕ = .028) significantly varied by condition. Thus there was no evidence of bias across conditions. The sample was also representative with 50.2% women compared to 51% of the population, but slightly younger at 36.47 years compared to the UK population average of 40.4 years. Age and sex were controlled in our mediation models.

.

Ethics oversight

Study 1: The field-based experiment was approved by The Australian Red Cross Lifeblood Ethics Committee on 7th May 2018 with the first randomized message sent out on the 22nd of May for those who had made a donation on the 16th of April (Reference: Davison 04052018).

Study 2: The implementation study is based on aggregate data at the population level and as such no individual are identifiable and donors consent for their data to be used for research when they sign up as a blood donor.

Study 3: The study was approved by the Australian Red Cross Lifeblood Human Research Ethics Committee on 31st July 2020

(Ethical approval number Guerin31072020).

Study 4: The study protocol was approved by Lifeblood's Human Research Ethics Committee on 18th September 2015 with reference number 2015#07.

Study 5: he study protocol was approved by Lifeblood's Human Research Ethics Committee on 15th November 2016 (Reference: 2016#24).

Study 6: The experiment was approved by the University of Nottingham, School of Psychology Ethics committee (ref: S1309).

Note that full information on the approval of the study protocol must also be provided in the manuscript.
